# Osteometric distinctions between domestic reindeer (*Rangifer tarandus tarandus*), wild mountain reindeer (*R.t.t.*), wild forest reindeer (*R.t. fennicus*), and the identification of castrated reindeer bones: Biometric explorations and archaeological methods

**DOI:** 10.1007/s12520-025-02198-3

**Published:** 2025-04-01

**Authors:** Mathilde van den Berg, Henri Wallen

**Affiliations:** 1 Archaeology, History, Culture and Communication Studies, Faculty of Humanities, University of Oulu, Oulu, Finland; 2 Arctic Centre, University of Lapland, Rovaniemi, Finland

**Keywords:** Domestication, Osteometry, Human-Reindeer Relationships, Sápmi, Cervidae, Reindeer Herding, Zooarchaeology, Arctic

## Abstract

The types of reindeer hunting, keeping, and herding in Fennoscandia have seen different periods of transformations and have found unique side by side expressions through time. To refine zooarchaeological analysis and scrutinize reindeer domestication and other past ancient human-reindeer relationships in the North, we propose methods for identifying sex, castration status, and ecotype/variety from complete and fragmented reindeer bones. This study examines the leg bones and pelvises of 161 reindeer from the Fennoscandian domestic reindeer (*Rangifer tarandus tarandus*), Norwegian wild mountain reindeer (*Rangifer tarandus tarandus*) and Finnish wild forest reindeer (*Rangifer tarandus fennicus*). We include intact males, castrated males, and females in our study. Ecotype (fennicus versus tarandus), variety (wild versus domestic tarandus), sex, and castration status are shown to influence bone growth in often element- and dimension-dependent ways. We demonstrate that metric variance is highest in fennicus and castrated domestic tarandus. Slenderness as expressed by diaphysis breadth–length index is sex and (albeit less) ecotype dependent, while distal breadth–bone length indices are mostly ecotype dependent. Scatterplots that combine slenderness with other measurement variables result in independent clustering between groups. The combination of two measurement variables facilitates ecotype/variety, sex, and castration status assignment due to independent clustering of groups. Our classification model based on isometric size and shape can be used to differentiate ecotype/variety, but not sex and castration status, due to limitation of group sizes. This study shows that reindeer ecotype, variety, sex, and castration status can be demonstrated through straightforward osteometric methods. We suggest cautious application in archaeological contexts because of (relative) changes in body size of past reindeer populations and our limited sample size, of especially wild male tarandus.

**Supplementary Information:**

The online version contains supplementary material available at 10.1007/s12520-025-02198-3.

## Introduction

This study explores the osteometric separation between modern castrated male, intact male and female domestic reindeer (*Rangifer tarandus tarandus*), male and female wild mountain reindeer (*Rangifer tarandus tarandus*), and male and female wild forest reindeer (*Rangifer tarandus fennicus*), based on postcranial skeletal measurements. Utilizing a variety of multivariable statistical methods and simple variable combinations, we introduce new biometric techniques for identifying castrated reindeer bones and determining ecotype, variety, and sex from fragmented archaeological reindeer bone assemblages in Fennoscandia. This is the first study to evaluate the osteological differences between castrated reindeer and male and female domestic, wild mountain, and wild Finnish forest reindeer. It builds upon Van den Berg et al.’s (2023) research on the identification of castrated reindeer from domestic reindeer bone assemblages but adds to it by including also wild reindeer. This paper presents methods for investigating the presence of castrated reindeer bones in the archaeological record and explores and presents novel approaches for differentiating between domestic reindeer, wild mountain reindeer, which is believed to be its progenitor (Nieminen and Helle 1980), and Finnish forest reindeer.

Reindeer have a long history of interaction with humans and can be found in association with our own species in archaeological assemblages since the Palaeolithic period onwards. Reindeer bones, teeth, and antlers are common portions of archaeological bone assemblages throughout the Northern Hemisphere. The hunting, keeping, and herding of reindeer have shaped the economic, social, and cosmological systems and modes and degree of mobility for many Indigenous communities in the circumpolar North (Skjenneberg and Slagsvold 1979, pp 1–3; Gordon 1990; Kofinas et al. 2000; Huntington and Fox 2005; Anderson et al. 2019; Salmi 2023). The reindeer’s importance comes from the animal as a source of meat, hide, milk, and transportation. The employment of reindeer has been central to the colonization of the northernmost parts of Eurasia (Helskog and Indrelid 2011; Bjørklund 2013). One of the major cultural transformations in this region has been the development of domesticating human-reindeer relationships (Krupnik 1993; Kofinas et al. 2000; Bjørklund 2013; Hansen and Olsen 2014). Researchers have debated the temporal, geographical, ideological, and contextual origins of these transformations and their subsequent transitions and geographical variation for over a century (e.g., Storli 1993; Kortesalmi 2008, pp 20–21).

Currently, reindeer continue to hold cultural significance for the Sámi, the Indigenous peoples of Fennoscandia. Outside of Fennoscandia another 23 ethnic groups live together with domestic reindeer in various forms of interdependence (Degteva et al. 2017). Recent work points out that reindeer had been harnessed for work as early as ca. 200 BC–160 AD in Siberia, as inferred by reindeer halter finds (Losey et al. 2020). The Sámi in Northeastern Fennoscandia employed working reindeer at least as early as 1300 AD, as inferred from pathological lesions and entheseal changes (Salmi et al. 2021). Indirect evidence of earlier use of draft reindeer in Fennoscandia can be seen through the identification of sleds similar to those used by the Sámi in later periods, found in graves dating from 900 AD onwards (Svestad 2018). Though direct empirical evidence is lacking, reindeer domestication is thought to have originated in Fennoscandia before the Late Iron Age (ca. 800–900 AD). The initial form of herding began with the keeping of small herds of relatively well-tamed and closely herded reindeer (Vorren 1973; Ingold 1986; Bjørklund 2013, p 177) mostly comprised of trained castrated males for transport. It is thought that domestication might have started with the taming of these castrated males (e.g., Ingold 1986; Bjørklund 2013; Van den Berg 2025, in press). Since then, the forms of reindeer herding in Fennoscandia have gone through several stages of transformations, and different types of hunting and herding have existed alongside each other in the region.

Answers to the questions on the shift from hunter-prey interactions to the origins of domestication and the variations of reindeer hunting and management systems remain obscure for both Fennoscandia and Siberia. The interpretation of reindeer bone finds in Fennoscandia is especially difficult because of three main reasons. Firstly, domestic reindeer lack clear domestication syndrome features which make domestic reindeer hardly distinguishable from wild mountain reindeer (Puputti and Niskanen 2009; Salmi et al. 2020; Pelletier et al. 2020). Secondly, the domestic and wild mountain reindeer as well as the forest reindeer are currently extant in Fennoscandia and past as well as present populations (still occasionally) interbreed and exhibit great morphological overlap (Nieminen and Helle 1980; Puputti and Niskanen 2009; Pelletier et al. 2021). Lastly, in the past, the different ecotypes occupied overlapping geographical distribution ranges (Nieminen and Helle 1980; Montonen 1974; Nieminen 1982a, b) and thus an admixture of these can be found among the bone assemblages of archaeological sites.

This study seeks, therefore, to develop novel methods to enable the osteometric separation of castrated, full male, and female members of the three ecotypes/varieties from archaeological reindeer bone finds. We consider castrated reindeer bones given the significance of identifying castrated reindeer in understanding the earliest stages of reindeer domestication. Additionally, our analysis aims to develop methods that can correctly identify the male and female remains of the domestic and wild varieties among mixed bone assemblages. In combination, these methods can help zooarchaeologists clarify the provenance and transformations of the diverse and mixed reindeer hunting, keeping, and herding cultures across past Fennoscandia. Our analyses offer perspectives to future osteometric studies aiming to differentiate between closely related reindeer types and other mammal species and subspecies. Furthermore, in considering the osteological effects of castration, it emphasizes the importance of individual life-histories of animals and how specific human-animal interactions induce archaeologically detectable physical changes during an animal’s lifetime.

## Materials and methods

### Reindeer in Fennoscandia

**Fig. 1:**
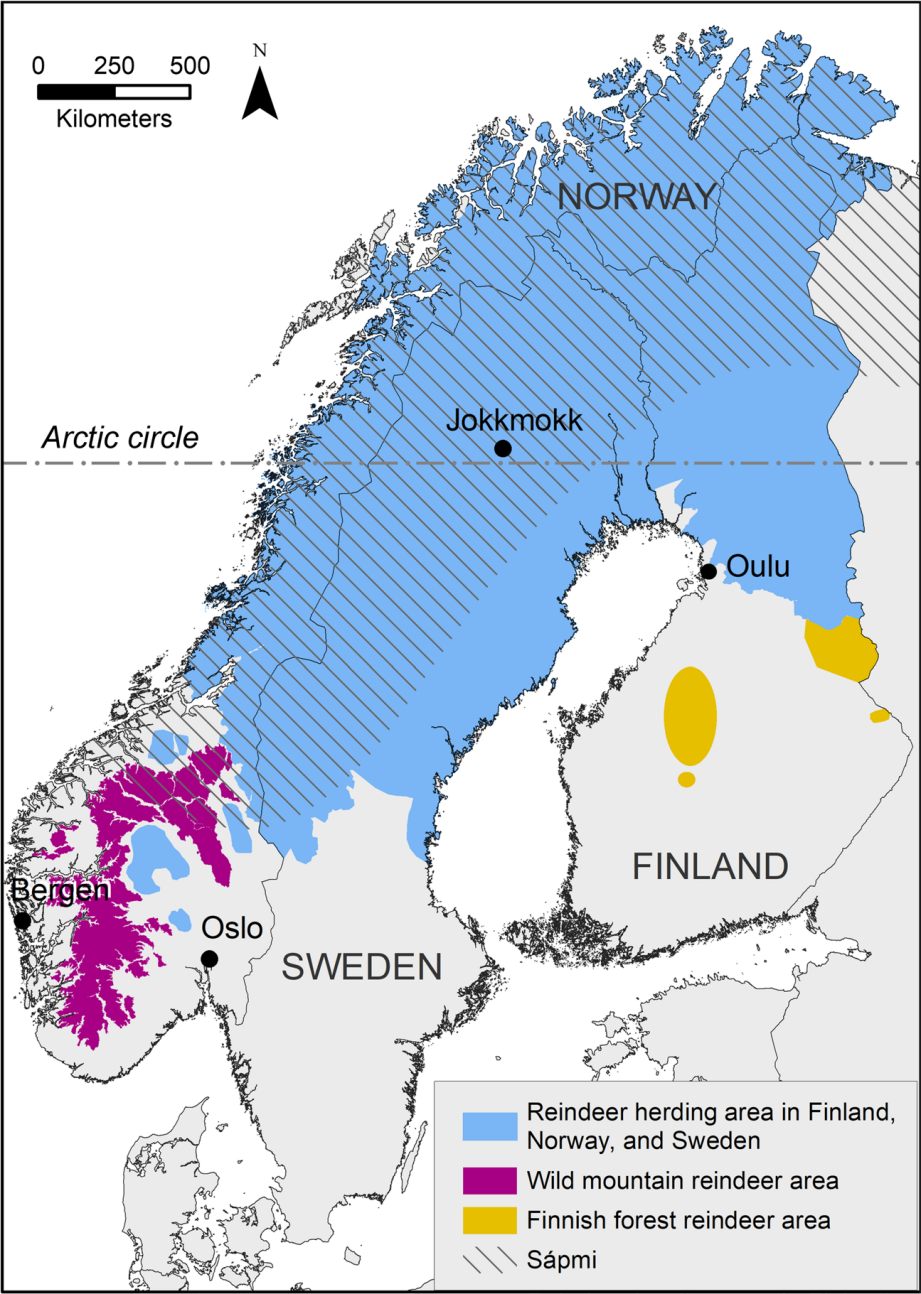
Present geographical distributions of domestic reindeer (*Rangifer tarandus tarandus*), Norwegian wild mountain reindeer (*Rangifer tarandus tarandus*) and Finnish forest reindeer (*Rangifer tarandus fennicus*) in Fennoscandia. Adapted from Ministry of Agriculture and Forestry 2007 (Finland); Villreinsenter 2024 (Norway); Paliskuntain yhdistys  2024 (Finland); Sametinget 2024 (Sweden)

Currently, there are three major types of reindeer extant in Fennoscandia (Fig. 1). Fennicus and tarandus have existed side by side in geographical Sápmi (the traditional homeland of the Sámi), Finland and Russian Karelia at various stages throughout the times. This has led to interbreeding and subsequential shared morphological features (Nieminen and Helle 1980; Hakala 1997; Hakala et al. 1996; Puputti and Niskanen 2009; Pelletier et al. 2021). It is thought that both ecotypes lived in the same areas in the forested regions of Sápmi even up until the nineteenth century (Nieminen 1982a; Heikura et al. 1985; Pulliainen and Leinonen 1990). The two ecotypes are frequently found together in archaeological assemblages, though it is usually not clear whether the tarandus are wild or domestic (e.g., Puputti and Niskanen 2009). Present populations of domestic reindeer and wild forest reindeer still occasionally mix in Finland as the tarandus reindeer herding area and the range of the wild forest reindeer are adjacent (Nieminen and Helle 1980; Ministry of Agriculture and Forestry 2007; Milla Niemi 2023, personal communication). There are currently two major forest reindeer subpopulations extant in Fennoscandia, located in southern and eastern Finland. They are managed by the Ministry of Agriculture and Forestry and are allowed to be hunted (Ministry of Agriculture and Forestry 2007).

Domestic reindeer were thought to have been domesticated from a (small) subpopulation of Norwegian wild mountain reindeer (Nieminen and Helle 1980; Røed et al. 2008). The conception of this argument was based on the morphological similarity between the two, in both post-cranial and skull measurements (Siivonen 1975; Nieminen 1977, 1980; Nieminen and Helle 1980) and the genetic compositions of current and ancient reindeer populations (Røed et al. 2008). More recently, Røed et al. (2021; 2022) have demonstrated a significant replacement of haplotype clusters during the eighteenth and nineteenth centuries. This suggests a distinct genetic shift at the advent of extensive reindeer pastoralism (Bjørnstad et al. 2012; Røed et al. 2018) and that the extant domestic reindeer populations are founded on a limited number of individuals, with maternal ancestry of partly non-native origin. Their origin suggests colonization from western Russia or the current taiga areas in Fennoscandia (Røed et al. 2022), of which the latter is currently the habitat of wild Finnish forest reindeer (Banfield 1961).

Present wild mountain reindeer populations are hunted and managed by owner organizations and state agencies and are found in 23 separated areas in the mountainous southern part of Norway (Reimers 2007) as well as on the Kola peninsula (Nieminen and Helle 1980). The reindeer herding area in Fennoscandia, excluding the Kola Peninsula, covers around 35%−50% of each countries’ land area, and is mainly practiced in the northern regions. The practice is highly integrated with the countries’ market economies and is a cultural keystone for the Sámi (Holand et al. 2022). Reindeer domestication most likely started with the taming of castrated males for transportation purposes in both Fennoscandia and Siberia (Ingold 1986; Bjørklund 2013, p 177; Van den Berg 2025, in press). Castration induces both physical and behavioural changes in the reindeer. It has played an essential and varied role in the different forms of domestic human-reindeer relationships. It is an integral component of all known past and present reindeer herding cultures around the globe and is an essential aspect of reindeer training, taming, control, herding strategies, ritual practices, and food production (Skjenneberg and Slagsvold 1979, pp 278–283; Paine 1994; Vitebsky 2005, p 279; Bjørklund 2013; Salmi et al. 2020; Soppela et al. 2022; Van den Berg 2025, in press).

### Previous research

The present study on the osteometric differences between domestic tarandus, wild tarandus and wild fennicus, as well as between the sexes and castration status, builds on our previous research (Van den Berg et al. 2023). There, we outlined the osteometric differences between females, castrated males and adult males of the Fennoscandian domestic reindeer using different approaches and developed methods to differentiate between these groups based on osteometric data. We based our analyses on the pelvis and leg bones of partial or complete skeletons of 97 adult modern domestic tarandus individuals. The (statistical) approaches that we used in our previous study and that we also used in this research are exploratory analyses using mean, range, and percent difference between groups (Sect. "Exploratory analysis"), and Mennerich's indices (1968) and simple variable combinations (Sect. "Mennerich’s indices and simple variable combinations"). In our previous study, we used penalised linear discriminant analysis (pLDA) to successfully discriminate between domestic female, full male and castrated male domestic reindeer bones. In the present study, we take a different approach to a classification model (Sect. "Statistical analysis") and instead use regularised discriminant analysis (RDA) based on Isometric Size and Shape. We use the same definitions of osteological measurements and intra-observer error data (Sect. "Osteological measurements" and SI1.1) as in our previous study. The first section of the Discussion (4.1) discusses the results of our previous study, and the following sections discuss the results of our and other previous work as integrated with the results of the present study.

### The reindeer sample

We measured 649 complete or partial humeri, radioulnae, metacarpi, femora, tibiae, metatarsi, and pelvises from 161 different reindeer individuals from the Fennoscandian domestic reindeer (Rangifer tarandus tarandus), Norwegian wild mountain reindeer (Rangifer tarandus tarandus), and Finnish wild forest reindeer (Rangifer tarandus fennicus) (Table 1; Supplementary Information Database). We included bones from the reindeer bone assemblages of the Biodiversity Unit of the University of Oulu, Finland, the Ájtte Swedish Sámi and Mountain Museum, Sweden, the Natural History Museum of Oslo, Norway, and the University Museum of the University of Bergen, Norway (Fig. 1). The collections are archived at the said institutions. We visited the collections in the period between the summer of 2019 and the autumn of 2023.

**Table 1:** We included 649 complete or partial limb bones and pelvises from the University of Oulu, the University Museum of Bergen, the Natural History Museum of Oslo, and the Ájtte Swedish Sámi and Mountain Museum

	Humerus	Radioulna	Metacarpus	Femur	Tibia	Metatarsus	Pelvis
*Rangifer tarandus tarandus* domestic	29	31	79	27	31	79	10
**Castrated male**	**9**	**9**	**25**	**8**	**9**	**25**	**1**
Ájtte Museum	0	0	3	0	0	5	0
University of Oulu	9	9	22	8	9	20	1
**Intact male**	**6**	**6**	**23**	**6**	**7**	**22**	**0**
Ájtte Museum	0	0	10	0	0	10	0
University Museum of Bergen	0	0	1	0	1	1	0
University of Oulu	6	6	12	6	6	11	0
**Female**	**14**	**16**	**31**	**13**	**15**	**32**	**9**
Ájtte Museum	0	0	14	0	0	12	0
University Museum of Bergen	3	3	3	2	3	3	2
University of Oulu	11	13	14	11	12	17	7
*Rangifer tarandus tarandus* wild	11	12	13	13	13	15	5
**Male**	**1**	**2**	**2**	**2**	**2**	**3**	**1**
Natural History Museum Oslo	1	1	2	1	1	2	1
University Museum of Bergen	0	1	0	1	1	1	0
**Female**	**10**	**10**	**11**	**11**	**11**	**12**	**4**
Natural History Museum Oslo	2	2	2	2	2	2	2
University Museum of Bergen	8	8	9	9	9	10	2
*Rangifer tarandus fennicus*	41	42	30	42	42	43	41
**Male**	**22**	**22**	**14**	**21**	**20**	**21**	**22**
University of Oulu	22	22	14	21	20	21	22
**Female**	**19**	**20**	**16**	**21**	**22**	**22**	**19**
University of Oulu	19	20	16	21	22	22	19
**Total**	**81**	**85**	**122**	**82**	**86**	**137**	**56**

We divided our sample into 7 groups based on sex, ecotype (between tarandus and fennicus), variety (between domestic and wild tarandus), and castration status (Table 2). Group 1 is castrated domestic reindeer (n = 30), group 2 is full male domestic reindeer (n = 25), group 3 is female domestic reindeer (n = 40), group 4 is male wild mountain reindeer (n = 3), group 5 is female wild mountain reindeer (n = 13), group 6 is male wild forest reindeer (n = 27), and group 7 is female wild forest reindeer (n = 23).

**Table 2:** The sample is organized into 7 groups based on ecotype, variety, sex, and castration status

	Group	N
*Rangifer tarandus tarandus* domestic		95
**Castrated male**	1	**30**
**Intact male**	2	**25**
**Female**	3	**40**
*Rangifer tarandus tarandus* wild		16
**Male**	4	**3**
**Female**	5	**13**
*Rangifer tarandus fennicus*		50
**Male**	6	**27**
**Female**	7	**23**
Total		161

Most specimens were of known age, sex, castration status and ecotype/variety. This information was provided by the curator of the collection. When age was unknown, only osteologically mature animals were used, i.e. fused epiphyses, see Hufthammer (1995) and Takken Beijersbergen and Hufthammer (2012). If the sex or ecotype was unknown, only specimens that very clearly belonged to a particular ecotype or sex were used. For example, male forest reindeer can be morphologically distinguished from male and female tarandus based on a combination of bone length/size and gracility, and female tarandus can be morphologically distinguished from male tarandus and female and male fennicus on a similar basis. Males and females of any ecotype/variety can be separated from the other sex by a trained zooarchaeologist based on gracility and long bone length if the ecotype/variety is known, the animal is osteologically mature, and complete bones are available. Sex can also be inferred from pelvic morphology. We did not distinguish between wild and domestic tarandus varieties in this way. Cases where we could not easily assign sex or ecotype morphologically were not included in our database. For example, male tarandus and female fennicus can have similar morphologies. We only applied this method to a limited number of specimens from Finland, from the University of Oulu collection.

Only animals castrated before 4, 5 years of age were included. The logical basis for this is that bone growth is linked to epiphyseal fusion (Silver 1963; Kennedy et al. 1999), and that epiphyseal fusion is influenced by castration (Popkin et al. 2012; Van den Berg et al. 2023). Castration can probably not be detected from bones when animals are castrated after epiphyseal fusion is completed. For reindeer, this means that castration is thought to affect bone growth only when performed before 4, 5 years old, which is when reindeer enter osteological adulthood (Hufthammer 1995; Takken Beijersbergen and Hufthammer 2012). It is generally thought that the osteological effects of castration are the more prominent the younger the animal is castrated (Telldahl et al. 2012; Van den Berg 2025, in press).

We treated the castrated reindeer sample as one group without differentiating castration ages because of the restricted sample size. We did not include hybrid individuals between domestic or wild mountain reindeer and forest reindeer. From a single individual either the left or the right element was used, so as not to include the same elements from one individual.

The osteological material utilized in this research study is sourced from diverse reindeer populations. The sample obtained from the Biodiversity Unit of the University of Oulu comprises individuals collected between the years 1963 and 2020, originating from Enontekiö, Hyrynsalmi, Ii, Inari, Ivalo, Kuhmo, Kuusamo, Oulu, Pudasjärvi, Simo, Suomussalmi, and Yli-Ii, all in Finland. The sample acquired from the Ájtte Museum collection consists of individuals collected between 1952 and 1955, originating from Älvsbyn, Funäsdalen, Hotagen sameby, Jänsmässholmens, Könkämä sameby, Luokta-Mavas sameby, Mittådalen, Rödingsträsk skogslappby, and Vittangi sameby, all in Sweden. The sample from the collection of the University Museum of the University of Bergen contains individuals hailed from Finnmark, Hardangervidda (Buskerud and Hordaland), and Rondane (Oppland), collected between 1869 and 2006, all in Norway. Lastly, the individuals sampled from the Natural History Museum of the University of Oslo collection originate from Oppland, Norway, and were collected between the years 1950 and 1965.

The sample size for various bone elements typically varied between 7 and 22 samples, with the majority of samples being from the female groups and the fewest from the castrated and full male groups. Unfortunately, we were unable to access more than 3 male wild mountain reindeer individuals. Due to its limited number of samples, the male wild mountain reindeer group and the pelvis bone were excluded from most statistical analyses.

### Osteological measurements

A total of 99 distinct measurements were included in this study, all of which have been previously defined by other studies. Measurements were recorded according to Schild (1962), Von den Driesch (1976), Davis (1996), Weinstock (1997), Weinstock (2000), Puputti and Niskanen (2008), Puputti and Niskanen (2009), SGWP in Popkin et al. (2012), Popkin et al. (2012), Telldahl et al. (2012), Niinimäki et al. (2021), and Van den Berg et al. (2023). The complete set of used measurements corresponds to the sets of measurements used in our previous study. See for the definitions of the measurements and visualizations on reindeer bone illustrations the Supplementary Information (Table SI1 and Figs. SI1-SI17).

The skeletal elements were measured using four distinct measuring instruments, each selected based on the specific type of measurement required. A digital caliper was employed to determine dimensions with an accuracy of one-tenth of a millimeter. A large-size caliper was utilized to obtain measurements rounded to the nearest millimeter. A measuring box was employed to record dimensions rounded to the nearest millimeter, and lastly, a tape measure was utilized to ascertain measurements also rounded to the nearest millimeter. Some bone elements could not be measured as whole because of breakage, pathological lesions, or the attachment of tissue/articular elements. In these cases, incomplete sets of measurements were used.

Intra-observer measurement error was tested for these measurements and results and calculations were published in our previous study (Van den Berg et al. 2023, Fig. 10). The findings are also presented in S1.1. The observer, the exact sets of measurements, and type of bone elements are the same for this study. These errors are taken into account when interpreting the osteometric separation between our groups.

### Analysis and statistical methods

#### Exploratory analysis

We used mean, minimum–maximum (range), and percentage difference between the different groups to examine the nature of our dataset and investigate the osteometric qualities of and between our groups per osteometric variable.

#### Mennerich’s indices and simple variable combinations

We applied Mennerich’s indices 1 and 3 (1968) to our dataset as a two-dimensional means to explore bone slenderness between our sexes and ecotype/variety. As the indices are ratios between different variables, they denote a measure of shape (morphology). This method has been used to identify different sexes and oxen in cattle bone assemblages (Albarella 1997; Telldahl et al. 2012; Rannamäe et al. 2022) and to compare dog morphotypes (Harcourt 1974; Mazzorin and Tagliacozzo 2000; Tourunen 2008). Mennerich’s indices were calculated in the following manner: Mennerich's index 1: the smallest diaphysis breadth (SD) × 100/greatest length (GL): $\frac{SD*100}{GL}$; Mennerich's index 3: distal breadth (Bd)/greatest length (GL) × 100: $\frac{Bd}{GL}*100$.

Similarly, combining measurements in scatterplots to understand bone properties between sexes, breeds, and subspecies is often employed (e.g., Boessneck et al. 1964; Guintard 1996; Guintard and Lallemand 2003; Telldahl et al. 2012). We combined different variables into scatterplots to explore osteometric properties between our groups and to explore which combinations of variables can help distinguish between our groups. These simple variable combinations are practical for their ease of use and because they can be performed on even seriously fragmented bones, i.e. a combination of only two different variables is used in each scatterplot. Clustering was assessed on a visual basis.

#### Statistical analysis

The study's analysis was organized into four main sections, employing the methodology outlined in Vigne (2015), based on the Mosimann (1970) protocol and expanded by Sampson and Siegel (1985). Following Vigne (2015, pp 135–136) we calculated Isometric Size Indices for each specimen as ${IS}_{i}= \frac{\sum_{1,n}\text{log}{V}_{i}}{n}$, where V_i_ are the values of measurements and n is the total number of variables. Then we calculated Shape indices (or Log Shape Ratios) for each specimen and variable as $LS{R}_{i,n}= log {V}_{1,n}- I{S}_{1,i}$. Isometric Size and Shape indices calculated as such, are theoretically independent from each other. These indices were then used for the statistical analysis using Regularized Discriminant Analysis (RDA). Statistical analyses were conducted in the R programming environment (R Core Team 2023). Discriminant analysis was done using R -package “discrim” (Hvitfeldt and Kuhn 2023) within the tidymodels -environment (Kuhn et al. 2020).

Initially, a classification model was used to distinguish between ecotype/variety. The second part of the analysis utilized a '1 versus rest' classification scheme, which set apart castrated specimens from the rest within the dataset. The study's final part aimed at ecotype/variety recognition through a condensed array of variables to emulate the often incomplete bone finds from archaeological contexts. These variables were methodically arranged in alignment with different bone regions: proximal, distal, and diaphysis, as detailed in the Supplementary Information Table SI2. A variable subset pertinent to intact bones was also included to gauge the effectiveness of limited variables on complete specimens.

The development of classification models for the purpose of identifying ecotype/variety and their respective group combinations encountered significant limitations within our dataset. The primary impediment stemmed from the insufficient size of the groups corresponding to each ecotype/variety and sex class, which also includes castrated specimens as a distinct group. This limitation in group size critically undermines the reliability of any analytical results and inflates the uncertainty of any parameter estimates. In statistical terms, the small sample sizes within each subgroup fail to provide the robustness needed for the models to effectively differentiate and classify with a high degree of accuracy.

Our methodological approach began by training multiple models on the allocated training dataset with 80/20 train/test split stratified by ‘ecotype/variety’ and ‘sex’. This was followed by a validation phase using tenfold cross-validation, a well-regarded method for minimizing both bias and variance in the assessment of model performance. After the validation phase, we selected the most effective model for further evaluation, using the test dataset.

## Biometric and statistical results

### Descriptive statistics of size

**Fig. 2:**
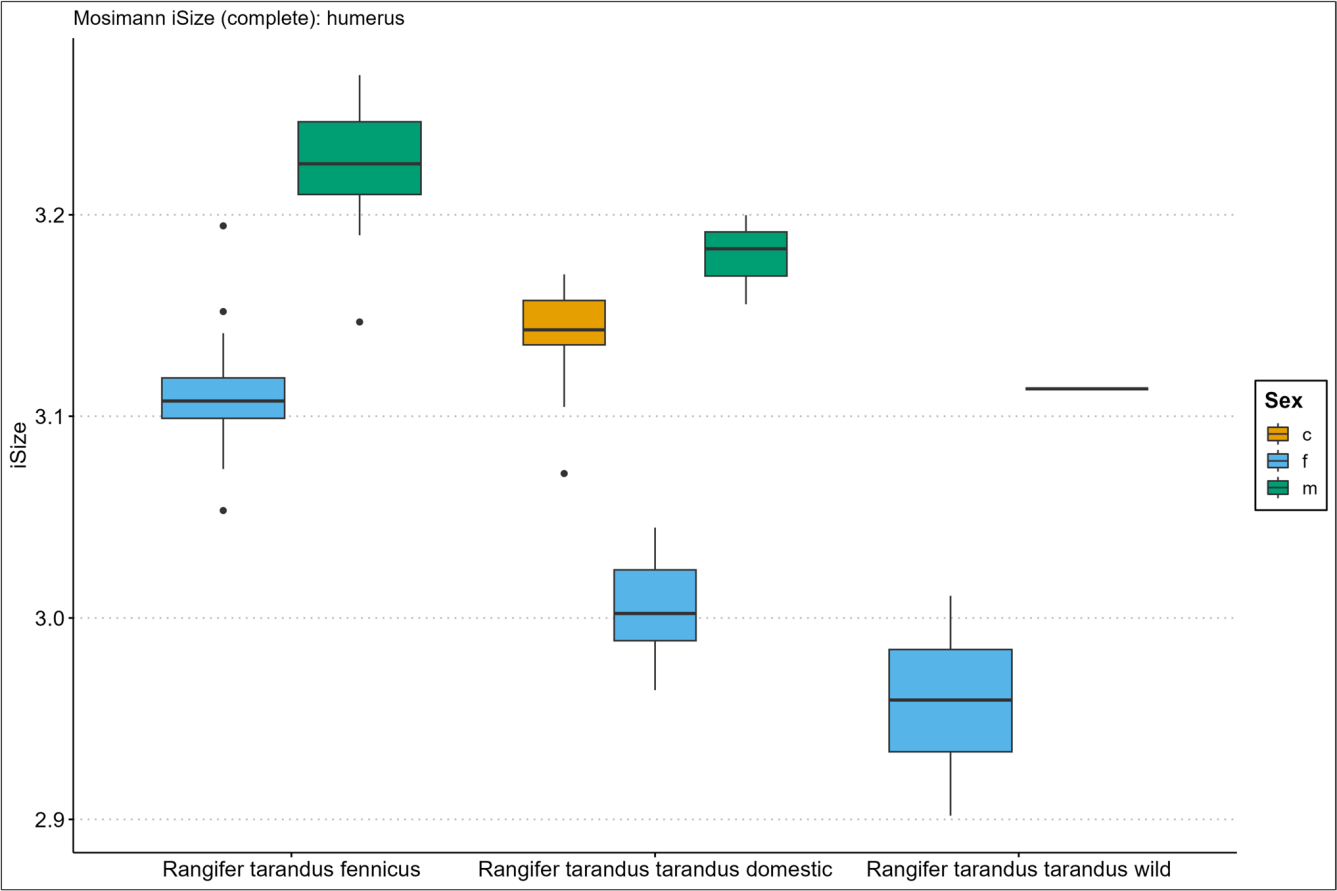
Boxplot results of the isometric size (iSize) analysis based on Mosimann’s (1970) protocol on the complete humerus bone for *Rangifer tarandus fennicus*, domestic *Rangifer tarandus tarandus*, and wild *Rangifer tarandus tarandus* (c = castrated, f = female, m = male)

**Fig. 3:**
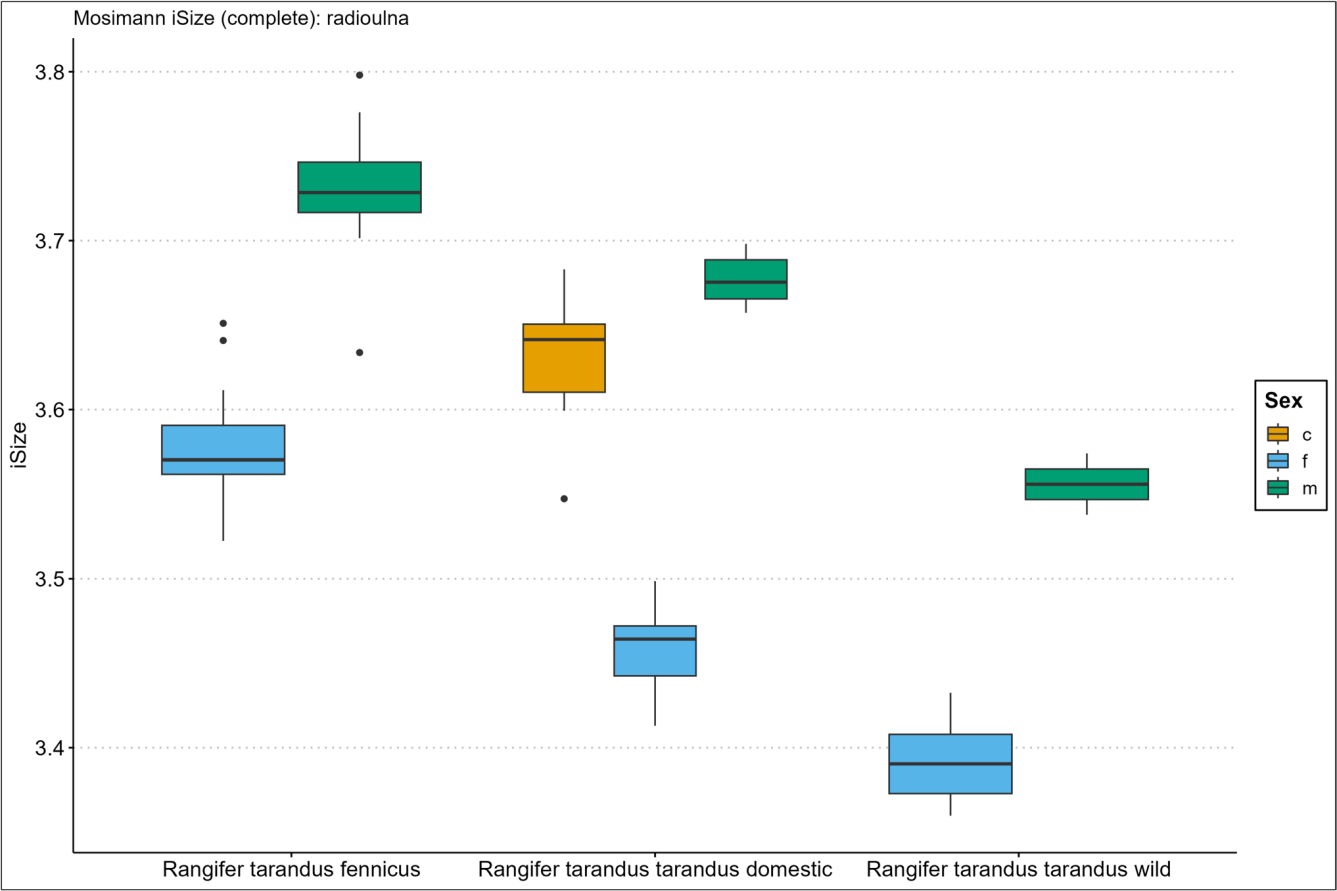
Boxplot results of the isometric size (iSize) analysis based on Mosimann’s (1970) protocol on the complete radioulna bone for *Rangifer tarandus fennicus*, domestic *Rangifer tarandus tarandus*, and wild *Rangifer tarandus tarandus* (c = castrated, f = female, m = male)

**Fig. 4:**
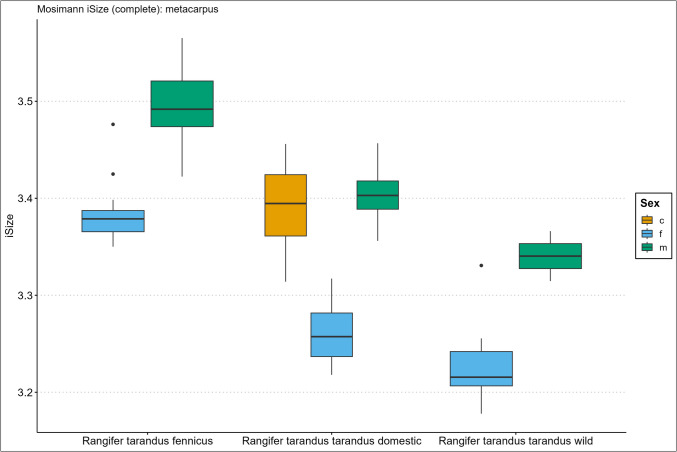
Boxplot results of the isometric size (iSize) analysis based on Mosimann’s (1970) protocol on the complete metacarpus bone for *Rangifer tarandus fennicus*, domestic *Rangifer tarandus tarandus*, and wild *Rangifer tarandus tarandus* (c = castrated, f = female, m = male)

**Fig. 5:**
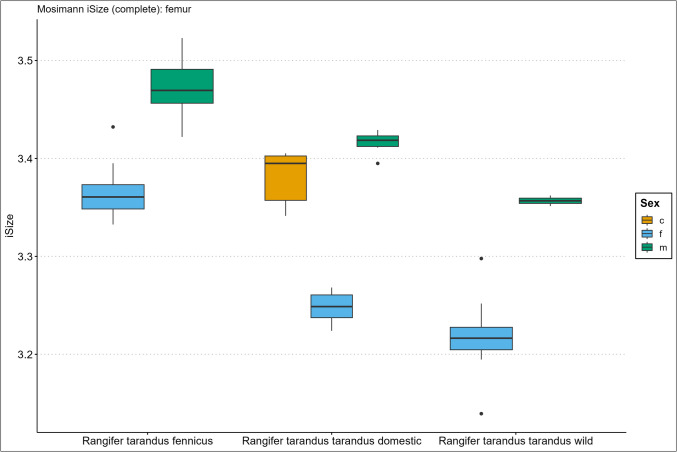
Boxplot results of the isometric size (iSize) analysis based on Mosimann’s (1970) protocol on the complete femur bone for *Rangifer tarandus fennicus*, domestic *Rangifer tarandus tarandus*, and wild *Rangifer tarandus tarandus* (c = castrated, f = female, m = male)

**Fig. 6:**
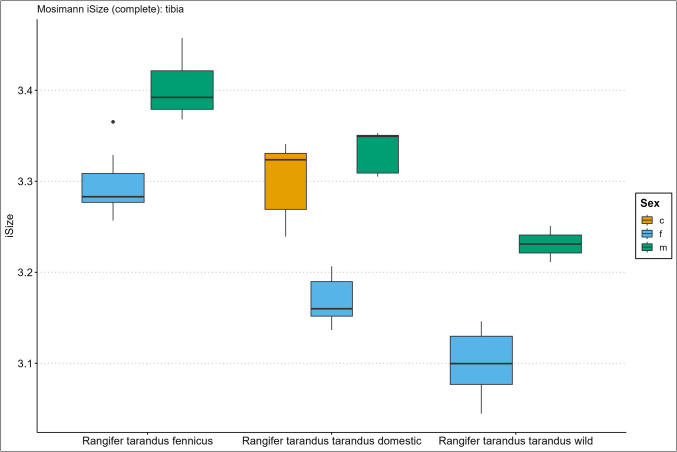
Boxplot results of the isometric size (iSize) analysis based on Mosimann’s (1970) protocol on the complete tibia bone for *Rangifer tarandus fennicus*, domestic *Rangifer tarandus tarandus*, and wild *Rangifer tarandus tarandus* (c = castrated, f = female, m = male)

**Fig. 7:**
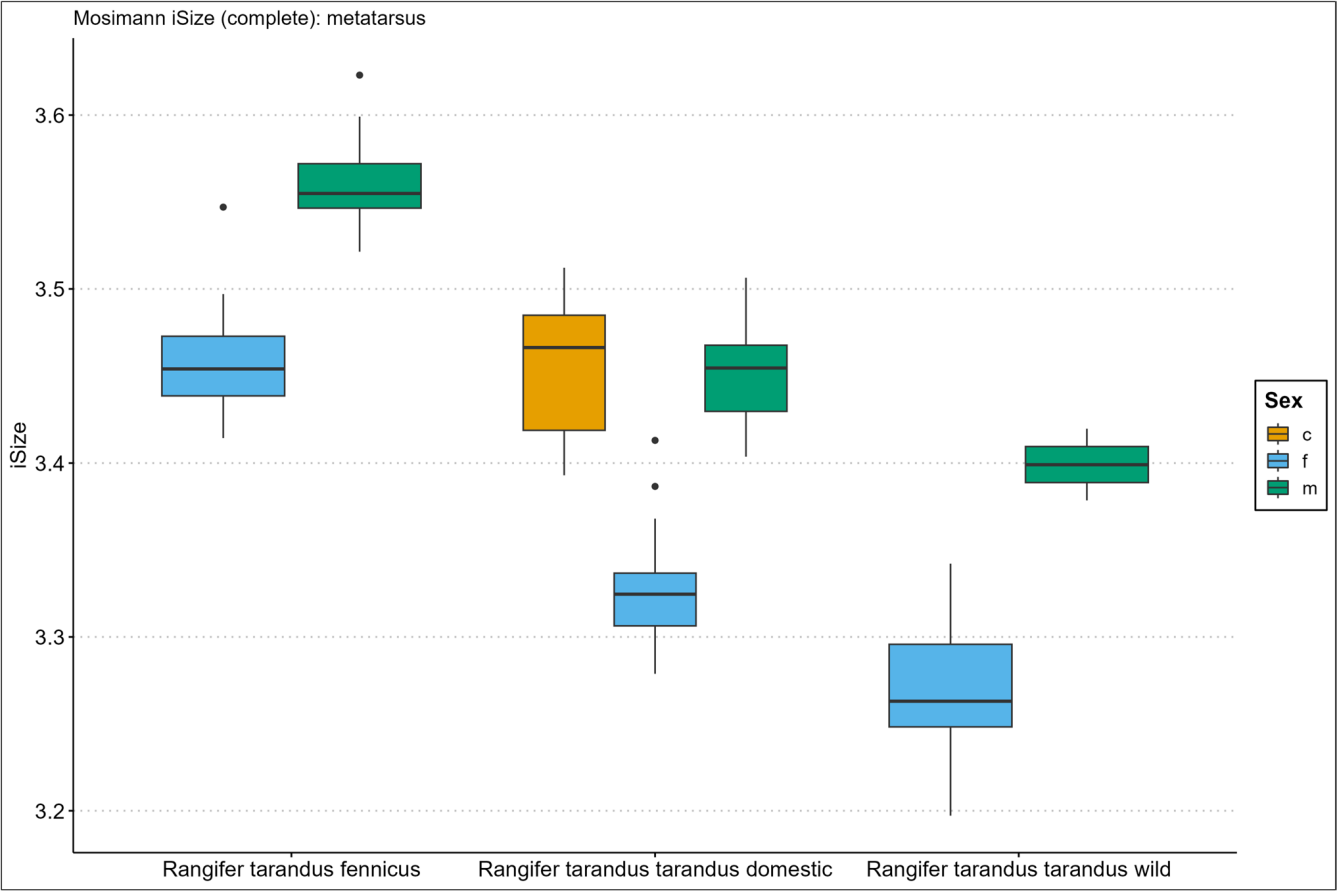
Boxplot results of the isometric size (iSize) analysis based on Mosimann’s (1970) protocol on the complete metatarsus bone for *Rangifer tarandus fennicus*, domestic *Rangifer tarandus tarandus*, and wild *Rangifer tarandus tarandus* (c = castrated, f = female, m = male)

Descriptive size statistics are presented in the Supplementary Information Spreadsheet, arranged per element, and Figs. 2, 3, 4, 5, 6, and 7 in the form of the iSize boxplot results per element. The boxplot iSize results on separate proximal, distal, and shaft parts as well as on the reduced set of variables for complete bones can be viewed in our GitHub repository.

Our new results of the iSize boxplot data in Figs. 2, 3, 4, 5, 6, and 7 confirm these previous notions regarding osteometric characteristics of and (dis)similarity between domestic male, female, and castrated reindeer (see 3.5 Mosimann’s iSize).

When we integrate this with our new data and compare this to fennicus, the general impression is that in overall domestic reindeer are more biometrically dimorphic than wild forest reindeer.

Within wild forest reindeer, the general picture emerges that this ecotype also demonstrates stark dimorphic traits between males and females in all our analysed bone elements and are easily differentiated based on size. Females are smaller in size in all dimensions and greatest separation is found in the diaphysis measurements, with females possessing slimmer diaphysis. For example, the smallest circumference of the radioulnar diaphysis (CD) has a min–max range of 6.7–8.3 and 8.7–10.6 cm, and the CD of the tibia has a min–max range of 6.6–7.8 and 7.8–9.2 cm, for females and males respectively. Least separation is found in the metapodial length dimensions with considerable metric overlap between the sexes. Humerus and radioulna also show considerable length overlap but greater separation is shown in the upper hind leg bones – femur and tibia. Breadth and depth measurements of the epiphyses show that females have smaller proximal and distal dimensions for all long bones but that the metacarpus and metatarsus again show greatest overlap in that regard. The pelvic bone shows the greatest biometric distinction for the DPS, Dam, and smallest breadth of the shaft ilium (SB). Although the greatest separation is found here, the sexes still show overlap in linear proportions for also these measurements.

Due to our limited sample size, we are not in the position to make strong statements about within sex size differences of wild mountain reindeer or size differences between the male wild mountain reindeer group and other ecotype/variety groups. The main issue of our small sample size is the narrow range and poor variance. However, we use the wild male tarandus results as a tentative indication. Our wild tarandus data indicates that this variety exhibits similar dimorphic traits as our domestic and wild forest reindeer, with females being smaller in size than males. The sexes are easily distinguished from each other based on single measurements due to the limited overlap. Greatest separation is again found in the diaphysis measurements, with females displaying slimmer long bone shafts than their male counterparts. For example, the CD of the radioulna is 5.5–6.8 and 7.5–7.7cm for females and males respectively. Length measurements show greater sexual dimorphism in wild tarand*us* than in fennicus and domestic reindeer. That is to say, not any linear overlap (and therefore males and females can be distinguished based on any length measurement).

Depth and breadth dimensions show small overlap between the male and female groups, of which most range overlap is found in the metapodial elements, as in domestic tarandus and fennicus. For example, the proximal breadth (Bp) of the humerus and femur (58.9–67, 9 and 72.4–73.2cm for females and males respectively) show great separation and thus potential suitability for osteometric differentiation.

The Supplementary Information Spreadsheet also shows the osteometric %differences and ranges of overlap between the group samples. Based on this data and on the biometric totals (the iSizes of Mossiman), of the humerus, radioulna, femur, tibia, and femur, the reindeer sexes and ecotypes/varieties can be arranged from largest to smallest in the following order: male Finnish forest reindeer, male domestic reindeer, castrated domestic reindeer, female Finnish forest reindeer, male wild mountain reindeer, female domestic reindeer, and lastly, the female wild mountain reindeer. Furthermore, castrated reindeer show greatest osteological overlap with domestic full males, wild mountain males, and Finnish forest females (except in diaphysis measurements). Metacarpus and metatarsus conform to this size evaluation except that the castrated group exhibits more overlap with female fennicus as well as with male domestic reindeer, naturally meaning that female fenn*icus* and male domestic reindeer also show greater overlapping biometric ranges for the metapodials between each other. Relatively, castrate metatarsals are a bit bigger in size relative to domestic males, while metacarpus shows broad overlapping ranges. In tibia specifically, our castrated group is osteometrically most similar to female forest reindeer.

We expect for the osteometric identification of reindeer bones from samples of unknown sex and ecotype/variety that male fennicus and female wild mountain reindeer are easiest identified. We expect most difficulty in differentiating between castrated domestic, male domestic, female forest, and male wild mountain reindeer because of their metric similarity. Our results show the relatively long leg length and larger body sizes of fennicus over tarandus, as well as the relatively small bone sizes of wild tarandus to its domestic counterpart.

### Mennerich’s indices

**Fig. 8:**
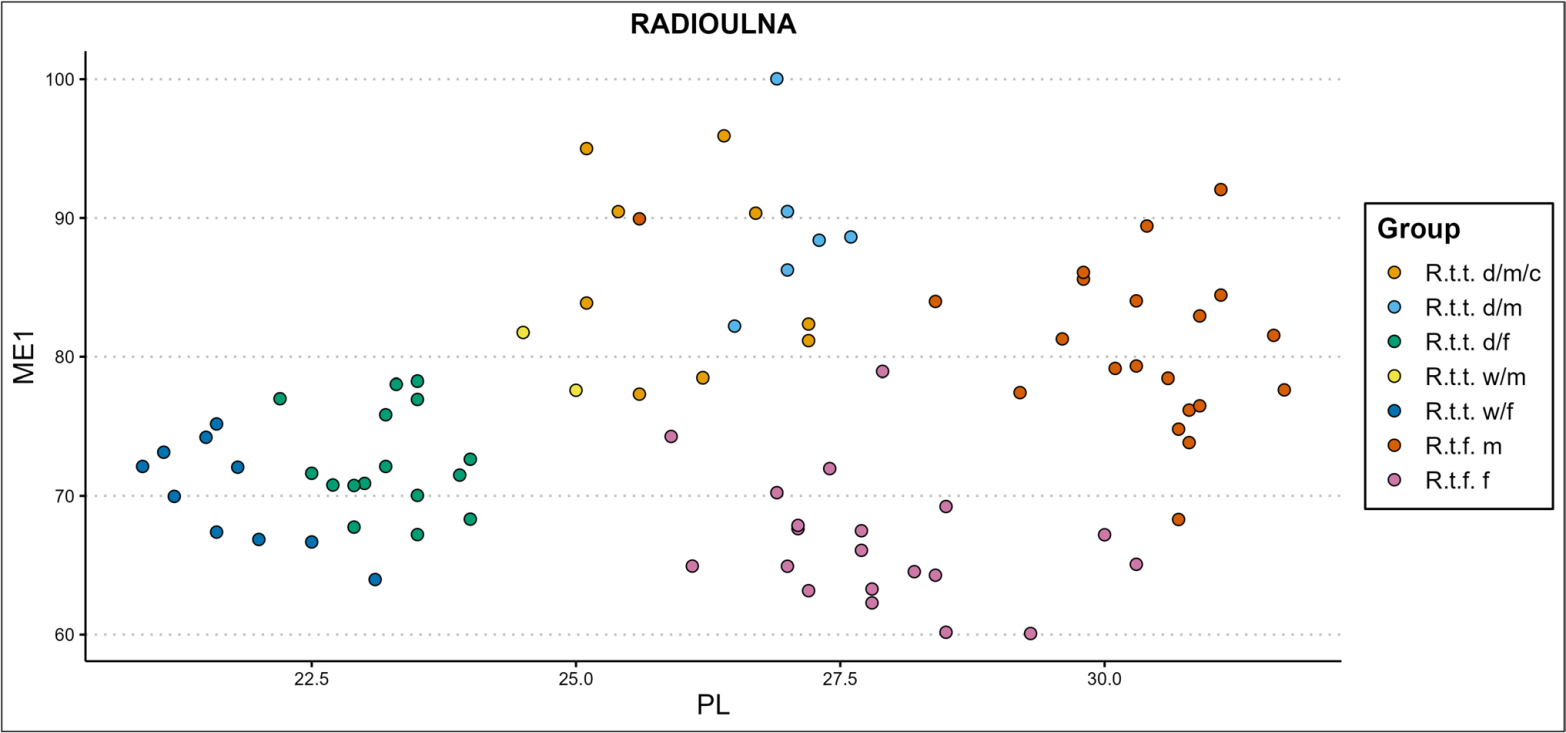
The radioulnar ME1 plotted against the physiological length (PL) of the radius, in centimetres (d = domestic, w = wild, c = castrated, f = female, m = male)

**Fig. 9:**
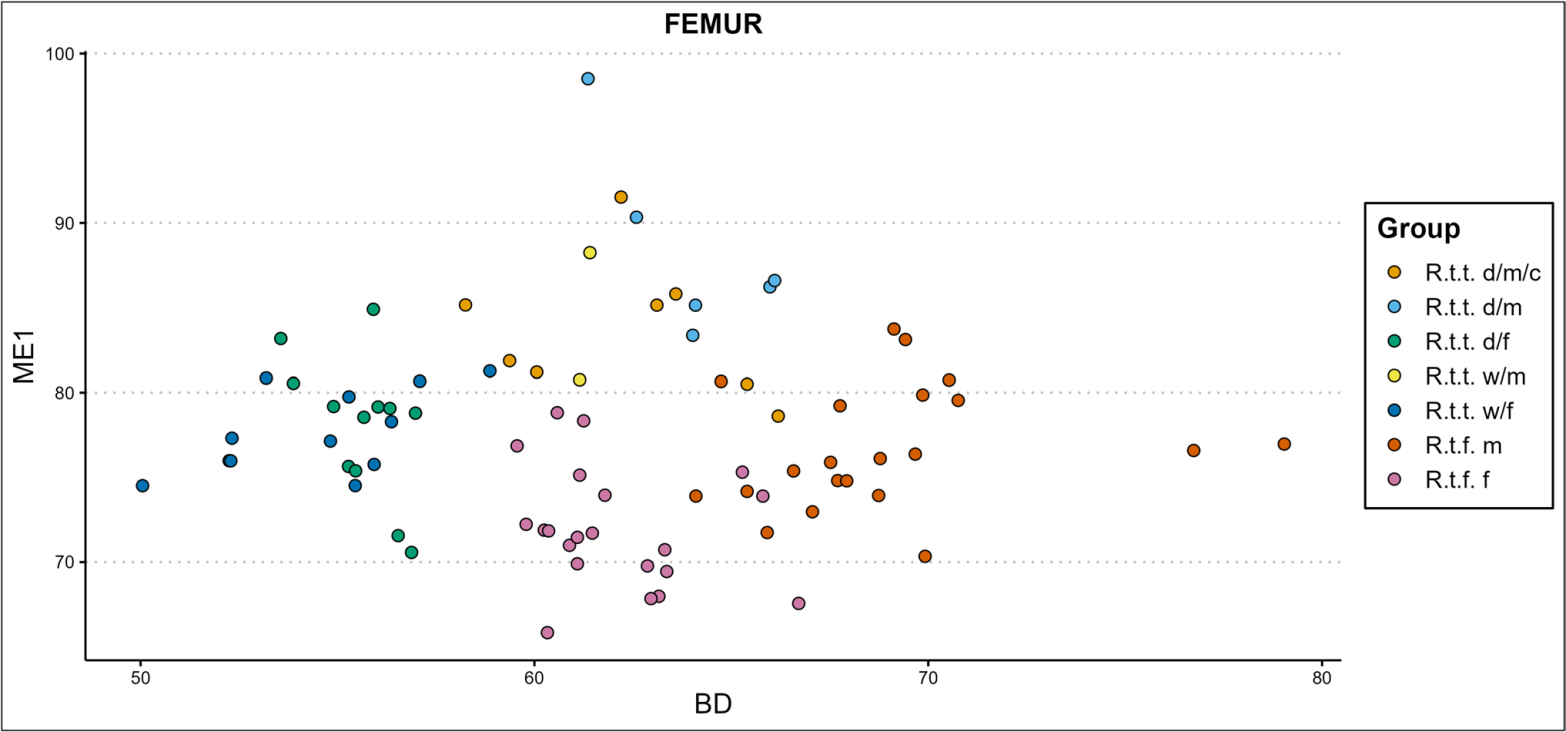
The femoral ME1 plotted against the distal breadth (Bd) of the femur, in millimetres (d = domestic, w = wild, c = castrated, f = female, m = male)

**Fig. 10:**
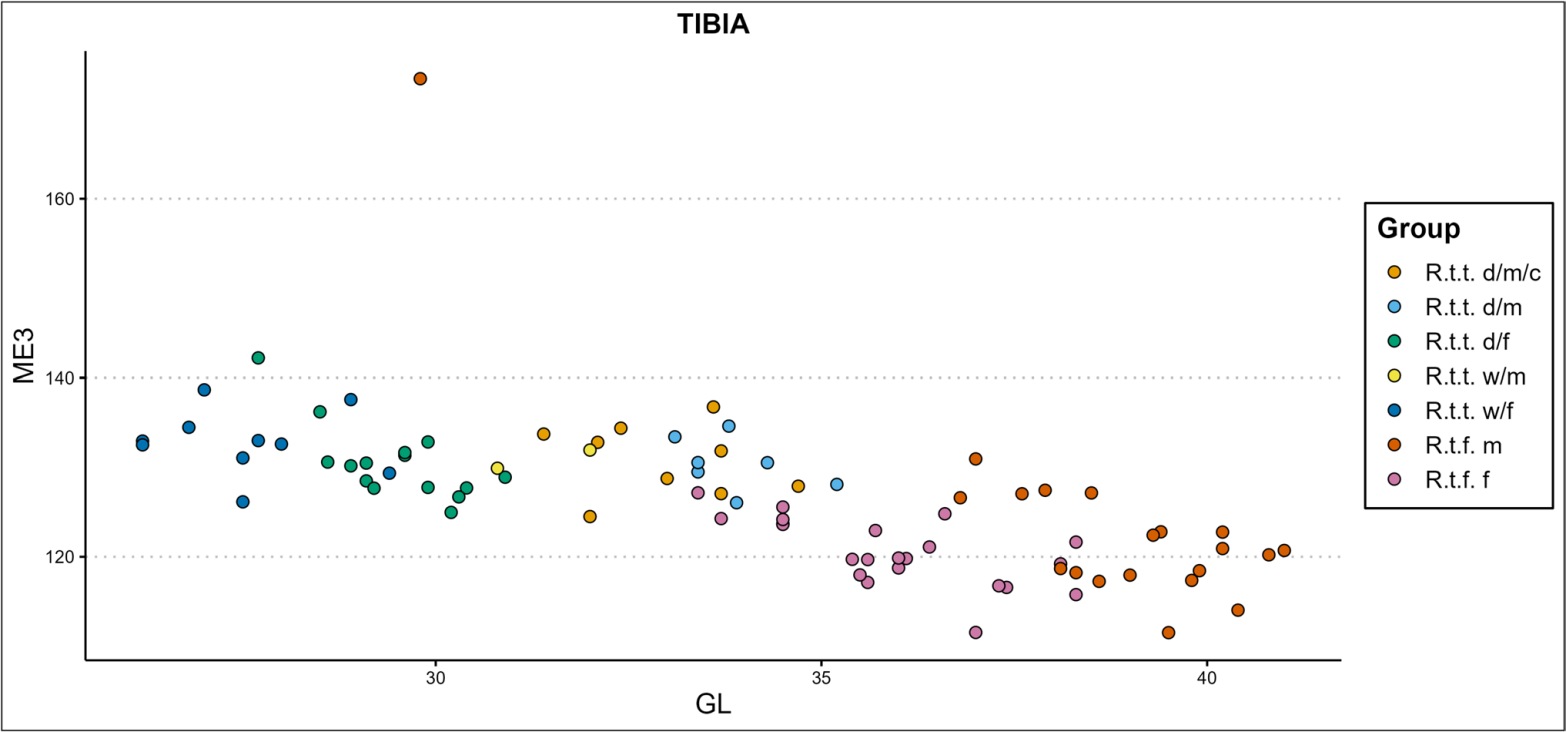
The ME3 of the tibia plotted against the element’s greatest length (GL), in centimetres (d = domestic, w = wild, c = castrated, f = female, m = male)

Mennerich’s indices (1968) show our bone’s slenderness per ecotype/variety, sex, and castration status group by combining diaphysis breadth with the greatest length (index 1 – ME1), and distal breadth of the element with the greatest length (index 3 – ME3). Some of our results are presented in Fig. 8–10., while all scatterplots of the Mennerich’s indices results plotted against osteometric variables can be found in our GitHub repository. Best results for sex, castration status, and ecotype and variety indication are attained when the size-independent calculations of bone slenderness are plotted against other variables to thereby create three-dimensional comparisons. It however depends on how slenderness is calculated for differences between groups is not equally expressed among different slenderness indicators.

Wild mountain reindeer—ME1 generally shows similar gracility between female domestic reindeer and female wild mountain reindeer, with females being slenderer than males. Ranges between the two types fully overlap, but for three female wild mountain reindeer specimens that show consistent higher gracility. Our two male wild mountain reindeer specimens overlap in gracility with the domestic castrate and full male groups, and with the more robust domestic females and female wild mountain reindeer. In radioulna and tibia, the male wild mountain reindeer show similar gracility with the most robust female tarandus groups, and the castrated domestic males. Slenderness inferred from ME3 on the humerus, femur, tibia, and metapodials shows that female wild mountain reindeer and female domestic reindeer have similar distal breadth – bone length proportions, with a subsection of domestic females possessing a slenderer morphology. Female wild mountain reindeer also show similar ME3 gracility to the domestic male reindeer groups. The small sample of male wild mountain reindeer that could be included shows slenderness likeness to domestic females, more robust male and female forest reindeer, more robust domestic females, or fully overlap with the domestic male groups, depending on the element. Radioulna presents an exception. A greater part of the female wild mountain reindeer is more robust than the domestic females, and a subsection of the more robust wild mountain females surpass all domestic males. They show instead overlap with only the more robust castrated reindeer.

Finnish forest reindeer – ME1 shows different levels of cluster separation between the slenderer female and more robust male Finnish forest reindeer. Most overlap between male and female clusters are found in the humerus, femur, and tibia, and greatest cluster separation are found in the radioulna and metapodials. Though ranges of slenderness partly overlap between female fennicus and female tarandus, female fennicus are found on the slenderest end of the ranges. The same counts for the three groups’ male counterparts: male fennicus overlap in slenderness with male wild and domestic tarandus, but only on their lower range of slenderness. Furthermore, male forest reindeer overlap in slenderness considerably with female domestic and female wild mountain reindeer in radioulna and tibia. In the femur and humerus, ranges partly overlap while a part of the male forest reindeer exhibit more gracile bone properties than both female tarandus groups. Castrated reindeer generally show likeness in gracility with the more robust or more gracile male forest reindeer, depending on the element. Slenderness inferred from ME3 on the humerus, femur, tibia, and metapodials shows that female and male forest reindeer almost fully overlap in their distal breadth to bone length ratio. The more robust male and female fennicus specimens overlap also with the slenderer specimens of the tarandus groups. For radioulna, a greater part of the female fennicus is more gracile than the male fennicus.

Based on the diaphysis breadth and length index (ME1) of the different limb bone elements, females are the slenderest from the sexes within each reindeer type, and fennicus is the most gracile from the types, while the two tarandus ecotypes show the most robust osteological qualities. Moreover, male fennicus shows in part similar gracility to female tarandus. This means that if an assemblage consists of different ecotypes/varieties and sexes, ecotype, variety, and sex can in most cases not be identified based on slenderness alone. However, if ME1 is plotted against osteometric variables such separation is possible. In the presence of also forest reindeer and wild tarandus, castrated domestic reindeer don’t show sufficient independent clustering to be separated on slenderness alone, but slenderness plotted against other variables shows better potential. In general, the ME1 gives clear clusters separating the two fennicus sexes from the two tarandus sexes, while mostly lumping the domestic and wild tarandus together, when plotted against almost any osteometric variable. For example, the ME1 of the radioulna plotted against the greatest physiological length (PL) of the radius (Fig. 8) and the ME1 of the femur plotted against the distal breadth (Bd) of the femur (Fig. 9) produce clear clustering. Gracility from the diaphysis breadth – greatest length plotted against other bone measurements thus produces suitable clustering for separation between fennicus and tarandus and their sexes but less between the two tarandus varieties. Individual bones can only be assigned to sex and ecotype/variety if falling clearly within one cluster. Using the ME1 as type and sex indication is more promising for larger datasets which might show cluster forming within the test/archaeological bone measurement assemblage.

Based on the breadth of the distal epiphysis and length index (ME3) of the different limb bone elements, male and female fennicus generally almost completely overlap in slenderness index range. The same general rule applies to the male and female domestic and female wild tarandus, though wild tarandus females are on average slightly more slender than the other tarandus groups. Forest reindeer are the most robust of the types. In the presence of one ecotype/variety, ecotype/variety and sex are not identifiable based on ME3 alone. Plotted against other osteometric variables, ME3 demonstrates less clear-cut cluster separations than ME1. Still, dependent on the bone element and to which variable ME3 is plotted against, the presence of different ecotypes/varieties and sexes can be demonstrated, though it is difficult to separate the two tarandus varieties (e.g., Fig. 10 ME3 of the tibia x Tibia GL).

Taking the ME1 and ME3 results together, it becomes apparent that for our dataset the diaphysis breadth–length index is both indicative of sex, and (but less) ecotype, while the distal breadth–bone length ratio is more ecotype dependent and sex independent. The exception to this rule is the radioulna, which also shows for the ME3 sex dependent cluster formations, as well as different cluster formations between wild and domestic tarandus females.

### Simple variable combinations

**Fig. 11:**
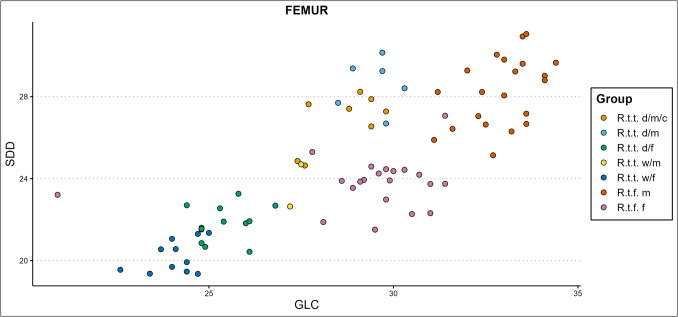
Simple variable combination scatterplot of the femoral greatest length from the caput (GLC) x smallest depth of the diaphysis (SDD) shows clear clusters for each ecotype, variety, and sex (d = domestic, w = wild, c = castrated, f = female, m = male)

**Fig. 12:**
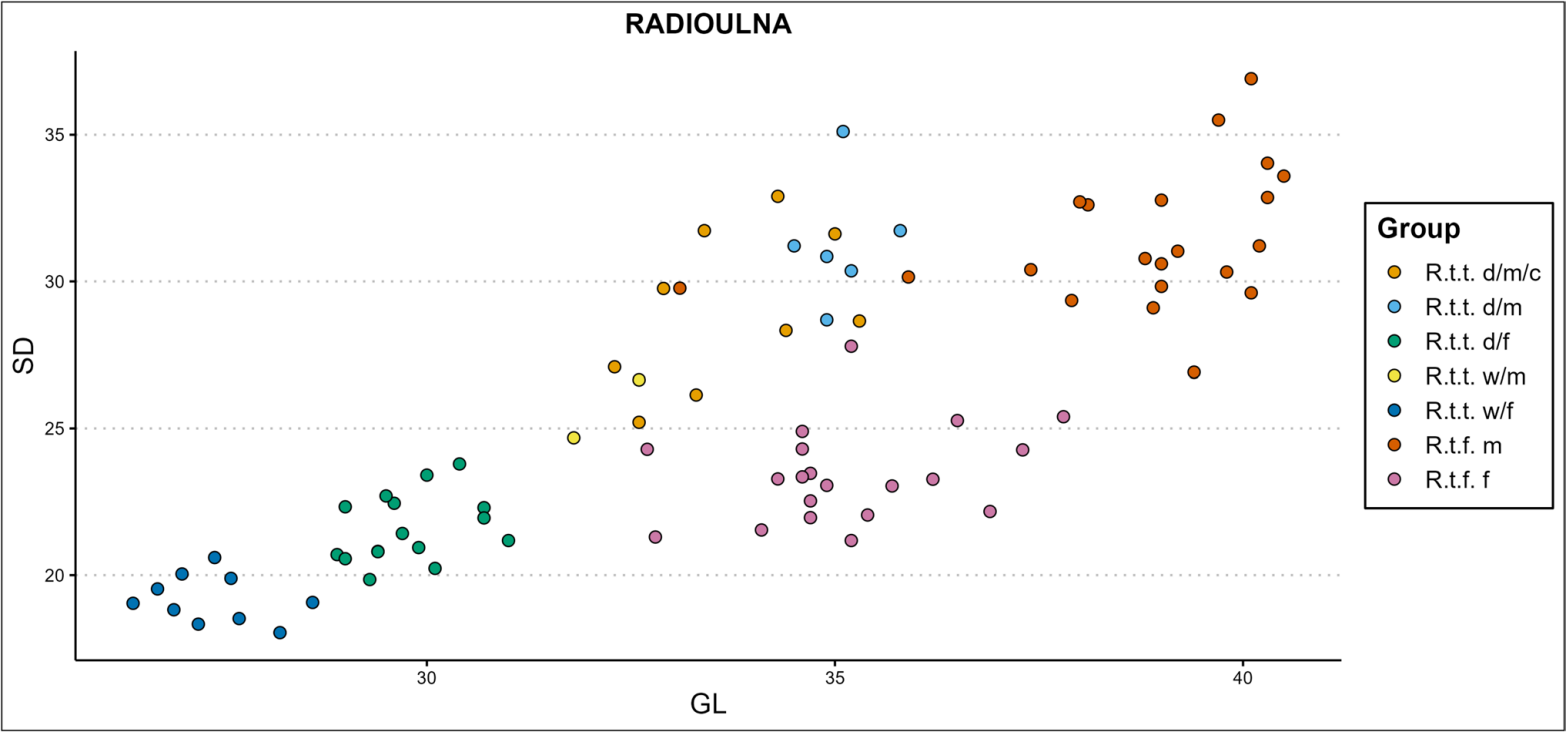
Simple variable combination scatterplot of the radioulnar greatest length (GL) x smallest breadth of the diaphysis (SD) shows clear clusters of sex, ecotype, and variety (d = domestic, w = wild, c = castrated, f = female, m = male)

**Fig. 13:**
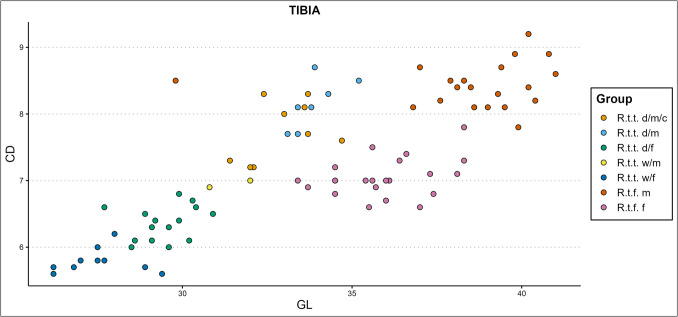
Simple variable combination scatterplot of the tibial circumference of the diaphysis (CD) x greatest length (GL) create clear and separate clusters for most groups, but combines castrated reindeer and male wild mountain reindeer (d = domestic, w = wild, c = castrated, f = female, m = male)

**Fig. 14:**
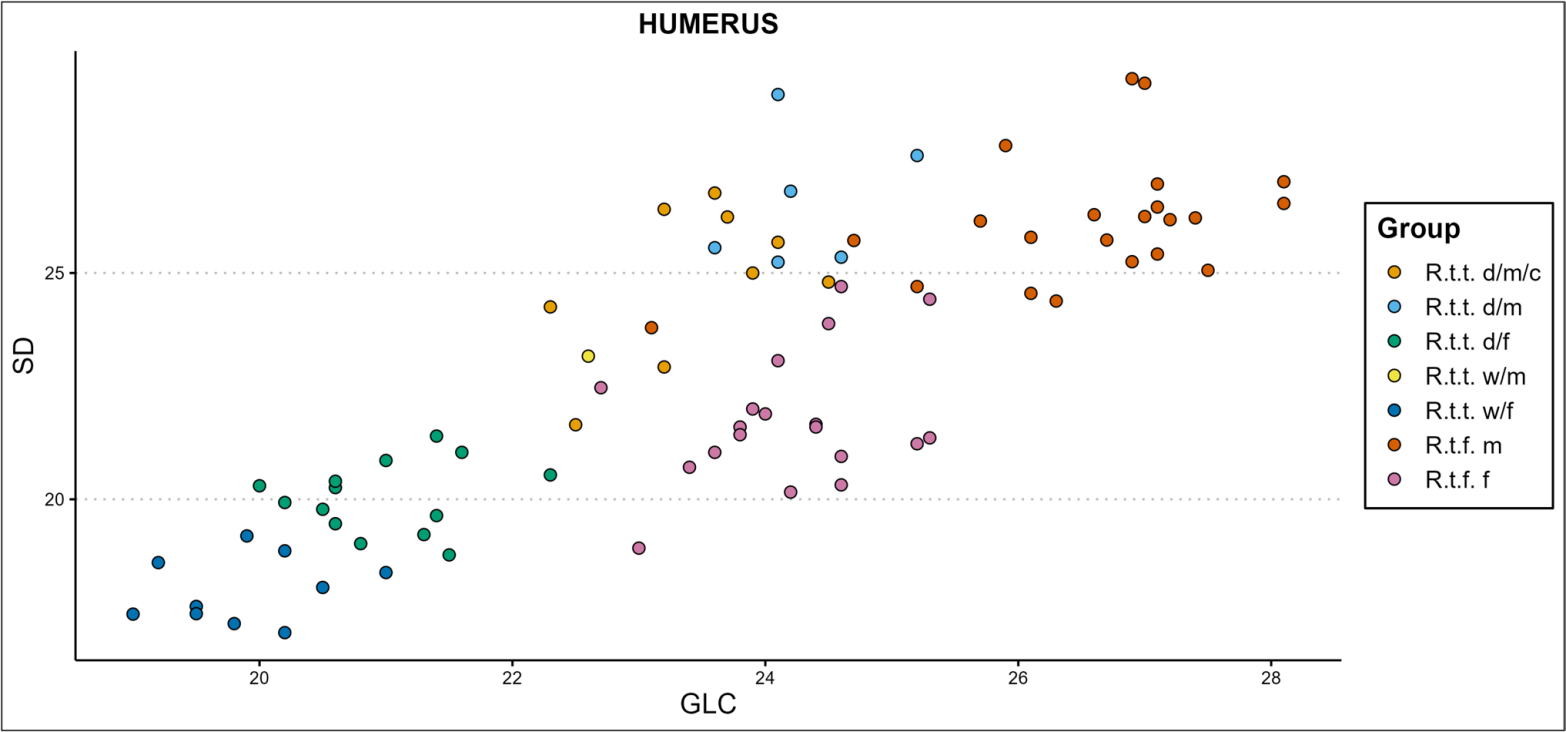
The humoral greatest length measured from the caput (GLC) x smallest breadth of the diaphysis (SD) is one of the variable combinations that show some independent clustering of the castrated reindeer group (d = domestic, w = wild, c = castrated, f = female, m = male)

**Fig. 15:**
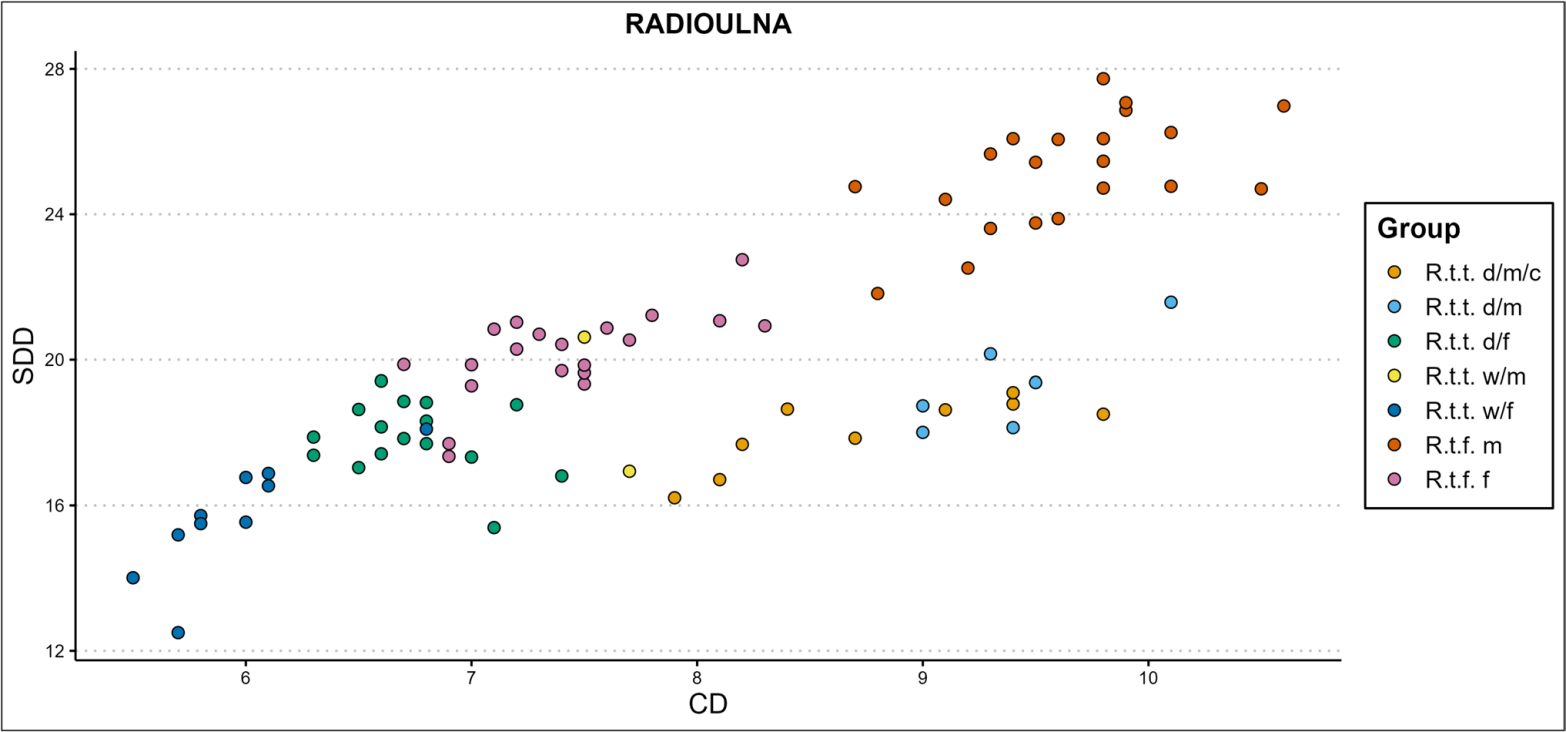
The radioulnar circumference of the diaphysis (CD) x smallest depth of the diaphysis (SDD) combines two diaphysis variables to create clear ecotype, variety, and sex dependent clustering, which also includes an elongated cluster of castrated specimens (d = domestic, w = wild, c = castrated, f = female, m = male)

Two osteometric measures from the same bone element are combined in scatter plots to attempt at sex and ecotype/variety differentiation. Some of our best results for the separation of ecotype/variety and sex (but not castration status) are shown in Figs. 11, 12, 13, 14, and 15. All scatterplots resulting from this study can be accessed via our GitHub repository. The trend of our new results for the separation of ecotype/variety and sex without regard of castration status, is that scatterplots from simple variable combinations create the best results when a measurement from the longitudinal axis is combined with a measurement from another axis, most notably a breadth measurement or a measure taken from the diaphysis. Scatterplots combining diaphysis measurements with other measurements also performed reasonably well. Diaphysis measurements show more indication to be mostly sex dependent and to a lesser extent ecotype/variety dependent (the radioulna is an exception in this regard). The overall indication of our results is that with the right variable combinations, clear clusters are formed between male forest reindeer, female forest reindeer, female domestic reindeer, female wild mountain reindeer, and a joined cluster of domestic castrated and full males. If only one ecotype/variety is present, sex indication and separation of our studied reindeer ecotypes and varieties can be reliably achieved through two-dimensional simple variable combinations, based on almost any combination of measurements within one bone element.

Domestic male reindeer (castrated and intact) appears in length very similar to female forest reindeer and is separated best from this group on the diaphysis measurements in combination with a measurement from a different bone part. Depending on the variable, this group occasionally forms an elongated cluster with female forest (most slender), castrated (in-between) and domestic intact males (most robust) regarding the diaphysis measurements. In general, the one or two male wild mountain reindeer specimens that could be included in the plots join either the female domestic, female forest, the combined castrated and intact domestic male cluster, or a small castrated male scatter, depending on the variable combination. Unfortunately, we could not obtain enough pelvis measurements from other groups besides female and male forest reindeer. Male and female forest reindeer form clear separate clusters within most pelvic measurement combinations, and the pelvis is thus, as expected, a useful element for sex separation. However, these are the only two groups that included enough specimens to draw meaningful conclusions.

Some of the variable combinations that worked well for group separation without special regard for castration are the femoral greatest length measured from the caput (GLC) x smallest breadth of the diaphysis (SD) or smallest depth of the diaphysis (GLC x SDD, Fig. 11), SD x physiological length (PL), or SDD x PL. Several of the more promising humeral variable combinations are the humoral circumference of the diaphysis (CD) x breadth of the trochlea (BT), CD x PL, GL x BT, GL x SD, height of the trochlear constriction (HTC) x PL, and the SD x BT. The metapodial simple variable combinations that work best are the metacarpal breadth of the proximal articular surface (BAp) x PL and BAp x SDD, and the metatarsal breadth of the diaphysis along the distal line of fusion (BDF) x PL, CD x depth of the lateral verticulus (DVl), and CD x PL. Some of the radioulnar scatterplots that show clear cluster forming are the GL x LO, PL x LO, GL x SD (Fig. 12), CD x SDD, PL x Bp, and SDD x LO. A selection of the tibial variable combinations that work well are the CD x PL, Dp x PL, GL x Bd, and SD x PL.

Some of the simple variable combinations that show reasonable separation of castrate clusters from other clusters are the radioulnar GL x LO, GL x SD, PL x LO, CD x SDD, PL x Bp; the tibial CD x PL, CD x GL (Fig. 13), GL x Bd, PL x BFp, PL x radioulnar breadth of the distal facet (BFd), and GL x Dp; the femoral Bp x PL, depth of the caput (DC) x SD, GLC x CD, and; the humoral CD x PL, GL x CD, and GLC x SD (Fig. 14). The best castration cluster separation is shown in combining length with other variables, most notably diaphysis measurements. Good results are also achieved through combining either diaphysis measurements or longitudinal measurements with medial–lateral or anterior–posterior measurements, meaning that useful cluster formations are found only from (near) complete bone variable combinations. One exception is the radioulnar CD x SDD (Fig. 15), which combines two diaphysis measurements. The least promising results are attained by combining medial–lateral with anterior–posterior variables.

### Mosimann’s iSize

Here we show the performance results of the iSize (Figs. 2, 3, 4, 5, 6, and 7) and iShape indices using the RDA for ecotype and variety separation using complete (Table 3) and different subsets of variables (Table 4–5). The classification models combine iSize and iShape, in which iSize consistently has the greatest predictive power (Fig. SI8-SI13 in Supplementary Information).

**Table 3:** The tested correct classification rates between the ecotypes and varieties for humerus, radioulna, metacarpus, femur, tibia, and metatarsus based on Mosimann’s iSize and iShape for complete bones in which all variables are included

Element	Variable set	Test/Train	Accuracy	Balanced accuracy	F1-measure
Humerus	All	Train	84.1	85.6	79.7
Humerus	All	Test	88.9	89.2	83.3
Radioulna	All	Train	86.4	87.8	81.1
Radioulna	All	Test	100.0	100.0	100.0
Metacarpus	All	Train	69.1	79.3	65.8
Metacarpus	All	Test	76.0	89.2	75.6
Femur	All	Train	90.6	91.7	88.7
Femur	All	Test	88.9	90.1	84.4
Tibia	All	Train	83.6	84.9	77.6
Tibia	All	Test	84.2	86.4	80.3
Metatarsus	All	Train	60.6	77.2	60.4
Metatarsus	All	Test	60.7	65.3	53.4

**Table 4:** The tested correct classification rates between the ecotypes/varieties for front limb bones humerus, radioulna, and metacarpus based on Mosimann’s iSize and iShape for distal, proximal, and shaft bone elements, as well as a small subset of variables for complete bones

Element	Variable set	Train/Test	Accuracy	Balanced accuracy	F1-measure
Humerus	Distal	Train	87.3	88.5	83.8
Humerus	Distal	Test	77.8	77.8	70.4
Humerus	Proximal	Train	77.8	79.1	70.7
Humerus	Proximal	Test	94.4	96.8	95.5
Humerus	Shaft	Train	88.9	89.2	86.1
Humerus	Shaft	Test	77.8	82.0	72.6
Humerus	Complete	Train	88.9	89.5	84.9
Humerus	Complete	Test	83.3	88.9	86.4
Radioulna	Distal	Train	62.1	69.2	58.4
Radioulna	Distal	Test	63.2	74.3	61.7
Radioulna	Proximal	Train	74.2	77.1	71.4
Radioulna	Proximal	Test	52.6	56.5	55.0
Radioulna	Shaft	Train	89.4	87.9	84.6
Radioulna	Shaft	Test	84.2	82.1	74.9
Radioulna	Complete	Train	83.3	84.7	76.8
Radioulna	Complete	Test	94.7	93.1	91.1
Metacarpus	Distal	Train	72.2	84.5	70.6
Metacarpus	Distal	Test	72.0	79.0	68.9
Metacarpus	Proximal	Train	69.1	82.4	67.8
Metacarpus	Proximal	Test	80.0	91.0	78.7
Metacarpus	Shaft	Train	72.2	80.9	68.5
Metacarpus	Shaft	Test	60.0	76.3	61.5
Metacarpus	Complete	Train	74.2	87.9	73.0
Metacarpus	Complete	Test	60.0	73.6	61.1

**Table 5:** The tested correct classification rates between the ecotypes/varieties for hind limb bones femur, tibia, and metatarsus based on Mosimann’s iSize and iShape for distal, proximal, and shaft bone elements, as well as a small subset of variables for complete bones

Element	Variable set	Train/Test	Accuracy	Balanced accuracy	F1-measure
Femur	Distal	Train	89.1	89.9	86.2
Femur	Distal	Test	94.4	96.8	95.5
Femur	Proximal	Train	89.1	89.6	85.9
Femur	Proximal	Test	94.4	96.8	95.5
Femur	Shaft	Train	87.5	89.5	84.2
Femur	Shaft	Test	88.9	92.9	87.7
Femur	Complete	Train	87.5	88.8	84.0
Femur	Complete	Test	94.4	96.8	95.5
Tibia	Distal	Train	86.6	87.8	81.5
Tibia	Distal	Test	84.2	82.9	75.5
Tibia	Proximal	Train	79.1	83.1	74.6
Tibia	Proximal	Test	73.7	75.8	63.4
Tibia	Shaft	Train	86.6	88.1	81.7
Tibia	Shaft	Test	78.9	82.2	72.8
Tibia	Complete	Train	88.1	89.1	83.6
Tibia	Complete	Test	89.5	93.2	86.1
Metatarsus	Distal	Train	61.5	77.4	60.7
Metatarsus	Distal	Test	64.3	70.8	58.5
Metatarsus	Proximal	Train	66.1	77.9	63.5
Metatarsus	Proximal	Test	53.6	74.6	55.2
Metatarsus	Shaft	Train	58.7	75.0	58.5
Metatarsus	Shaft	Test	75.0	80.4	67.7
Metatarsus	Complete	Train	60.6	74.8	59.6
Metatarsus	Complete	Test	71.4	83.2	68.2

Mosimann’s iSize box plots for the humerus, radioulna, metacarpus, femur, tibia, and metatarsal reveal distinct iSize differences within and between groups. Ecotype/variety classification rates in the complete elements model range as follows: humerus (79–84%), radioulna (81–100%), metacarpus (65–75%), femur (84–89%), tibia (77–81%), and metatarsus (53–61%). As anticipated from the iSize box plots, the metapodials show comparatively lower performance than other elements. The most useful element for ecotype/variety classification within this classification scheme is the radioulna. Our partial bone elements models (Table 4–5) perform exceptionally well for separate distal, proximal, and shaft portions of the humerus, femur, and tibia. The distal and proximal portions of the radioulna pose more difficulty together with the separate portions of the metapodials. The reduced variable selection models for complete bones also perform well, with 86–96% correct classifications for the humerus, radioulna, femur, and tibia.

Unfortunately, we found that our data set is not suited for identifying sex nor castration status. The main reason for this is that our sample sizes are too small, meaning that the test/train in 20/80 ratios subsets become too small to perform meaningful statistical analysis (see Table 2). From our previous sections on slenderness indices, descriptive statistics on size, and simple variable combinations as well as our iSize and iShape material we have learned that female fennicus, domestic males, and wild male tarandus bear stark resemblance in osteometric properties. This is a further reason why larger sample sizes are needed to train a model to differentiate between these groups.

## Discussion

### Discussion of our previous study on separation between domestic males, females, and castrates

#### Descriptive statistics on size

Between domestic males and females, our previous study (Van den Berg et al. 2023) and other studies (Puputti and Niskanen 2009; Pelletier 2020, 2021) clearly indicated that females are smaller in size in all bone elements and measurements and therefore easily separated from intact males. Especially depth and circumference show stark dimorphism. Males and females are easily distinguished based on length measurements of humerus, radioulna, femur, and tibia, if complete bones are available, as females have shorter long bones than and show no overlap with males. The pelvic bone showed the greatest biometric distinction for the greatest depth of the pubic symphysis (DPS) and depth of the medial rim of the acetabulum (Dam), which showed no metric overlap between the sexes, indicating easy within ecotype/variety separation based on these bone portions alone. Though certain overlap was present in some depth and breadth dimensions within all elements, most notably in the metapodials, overlapping ranges are generally relatively narrow.

We found that the most apparent effect of castration was that it inhibits growth, as castrated reindeer bones were usually slightly smaller in size than full males’, in all dimensions, with exceptions mostly in the metapodials (lower limb bones). In our previous study we found that greatest size differences between domestic males and castrates were found in the radioulna, humerus, and to lesser extent in the femur. That the least size differences were found in the metapodials was not surprising because they are early-fusing elements (Hufthammer 1995; Takken Beijersbergen and Hufthammer 2012), and thus have already matured when the animals are castrated between 3 and 4 years of age.

We found that castration affected the breadth, depth, and circumference variables the most, while length measurements were affected only marginally. This was in contrast with the osteological effects of castration reported in other species, for which previous studies had indicated that length became majorly affected after castration (e.g., Hobday 1914; Silberberg and Silberberg 1971; Davis 2000; Telldahl et al. 2012). We explained this to be partly because the species studied were castrated at an earlier age, whereas nowadays reindeer are usually castrated when they are close to skeletal maturity. Castration of reindeer at an earlier age is less common, but is also practiced, as it was in historical times (see Rönnow 1949; Carl Linnaeus 1732, in Graves 1995; Van den Berg et al. 2023; Van den Berg 2025, in press). It is important to note that the practice of castration at a certain age may have changed over time and may have differed between cultural or family groups.

The differences between females and castrates (Van den Berg et al. 2023), also clearly indicate that these two groups are easily distinguished from each other in size, with castrates leaning more towards male sizes than to female sizes. Only the smallest depth of the olecranon (SDO – radioulna) showed significant overlap between castrates and females in size. All in all, castrates show certain signs of femininization of their bone structure, but still exhibit considerable overlap with male bones in size. Length variables most notably show most overlap between the two male groups among dimensions.

#### Mennerich’s indices

Our previous study (Van den Berg et al. 2023) had demonstrated that domestic female reindeer have more slender bones in comparison to their male counterparts and are easily separated based on gracility (especially if plotted against another variable). The ME1 plots produced evident bimodal distributions for the two sexes. As a function of sexual hormones, castration influences the ME1 results of the humerus, radioulna, femur, and tibia. The plotted castrated reindeer appeared as an in-between slenderness group between full males and females. They were not easily separable from the male group based on our indices, but with inclusion of also females our slenderness scatterplots showed an elongated distribution between females (most slender), castrates (intermediate slenderness), and full males (most robust). The presence of not a bimodal distribution but an elongated scatter could thus indicate the presence of castrated domestic reindeer. This effect was most apparent for the radioulna and humerus. The metapodials showed a range of overlap between males and females, and thus an elongated scatter would not necessarily be an indication of the presence of castrated animals. ME3 indicated similar distal breadth – bone length proportions for female, castrated, and full male domestic reindeer and thus was found not useful in sex separation.

Not all castrated individuals showed an increase in ME1 slenderness (Van den Berg et al. 2023). Admittedly, this probably is due to the age at which castration is performed. Cupere et al. (2000), Telldahl et al. (2012) and Rannamäe et al. (2022) emphasized that only oxen castrated at an early age could be detected through slenderness, as it is widely known that osteometric properties are not influenced by castration after osteological maturity has been reached. The difference between cattle and reindeer is that cattle are often castrated early in life, while reindeer are usually castrated at near osteological maturity, which is reached at about 4, 5 years (Hufthammer 1995; Takken Beijersbergen and Hufthammer 2012). Reindeer are only castrated young in exceptional cases. Reindeer herders have communicated that if reindeer are castrated too young, they remain small, feminine, and young in both their body and in their mind (Van den Berg 2025, in press).

#### Simple variable combinations

Our previous study (Van den Berg et al. 2023) showed that simple variable combinations can be a powerful tool in detecting castration status within some bone elements. We found that within certain variable combinations castrates occupied the ‘space’ between male and female size, resulting sometimes in a trimodal distributions (radioulna, humerus) or elongated clusters (radioulna, humerus, femur, and tibia). Some combinations that worked well and which we highlighted in the previous paper were the humerus proximal depth (Dp) x proximal breadth (Bp), humerus distal breadth (Bd) x height of the trochlea (HT), radioulnar breadth of the proximal facet (BFp) x length of the olecranon (LO), radioulnar distal depth (Dd) x distal breadth (Bd), and femoral greatest length (GL) x Bd. The metapodials were the least suitable for indicating the presence of castrated reindeer bones.

### Discussion and integration

#### Classification models based on Mosimann’s iSize and iShape

Our classification model based on isometric Size and Shape may be used for ecotype/variety differentiation and has in this study shown to perform well on complete bones, as well as on separate proximal, distal, and shaft bone parts, and complete bones with reduced variable sets. The results of our model assessed through RDA suggest that ecotype/variety-based group separation is most effective if the complete radioulna is used, in which case every bone was correctly identified to ecotype/variety. We found the metapodials are least effective for these group separations. The model performed well for separate proximal, distal, and shaft bone parts of the radioulna, humerus, femur, and tibia and could thus be useful for application on fragmented bone assemblages. This is useful for archaeological application as Fennoscandian reindeer bone assemblages mostly consist of fragmented bones due to the usual procedure of marrow extraction (Harlin et al. 2019), though complete bones are found as well (Collinder 1949, p 136; Spencer 1978, p 69; Roué 2012, p 50).

Our used classification model cannot differentiate sex nor castration status, which is most likely due to our small sample sizes. We therefore suggest that for the osteometric identification of sex and castration status in reindeer, a combination of methods is used: 1) the statistical classification of ecotype/variety using Mosimann’s iSize and iShape classification models, after which 2) other osteometric methods such as simple variable combinations, Mennerich’s indices 1 and 3, or our previous LDA model (Van den Berg et al. 2023) for the identification of castrated reindeer from domestic reindeer assemblages, can be used to identify sex and/or the presence of castrates. Simple variable combinations and slenderness calculations (optionally plotted against another bone variable) can be used in isolation without our classification model. We expect that our classification model as well as other methods for group differentiation would be most reliably interpreted in conjunction with the use of additional analyses presented here. Future application of our methods in isolation and combination on archaeological assemblages will provide further inside into their usefulness.

#### Slenderness

We have shown that Mennerich’s bone slenderness versus robusticity indices based on bone length to diaphysis breadth (ME1)/distal breadth (ME3) ratio can be strong indicators of castration status, sex, and ecotype/variety. In particular, bone length to diaphysis breadth shows promising results for ecotype indication, as well as for sex and castration status indication. Bone length to distal breadth ratio was ecotype but not sex specific. Slenderness is an indication of ecotype between tarandus and fennicus but cannot differentiate between the domestic and wild tarandus varieties. Our analyses also have shown that morphological differences in slenderness between ecotypes or varieties can obscure differences between sex and castration status, and vice versa. Similar issue has been pointed out by Fock (1966) and Albarella (1997), who studied differences in bone slenderness between different sexes and breeds of cattle from metapodials.

Females consistently showed more ME1 slender bone morphologies than their male counterparts from the same ecotype or variety, while within our sample sex does not influence ME3. Previous biometric studies have also utilized Mennerich’s indices and found increased slenderness to be an indication of sex and castration in cattle (e.g., Telldahl et al 2012; Rannamäe et al. 2022). For most bone elements there is a partial overlap in ME1 slenderness between males and females, so individual bone elements can only be sexed based on ME1 slenderness if distinctly falling outside of this overlap. If the sample size is big enough, two sexes will present themselves in the form of a bimodal distribution.

Other studies have used the indices for dog breed indication and comparison (Harcourt 1974; Mazzorin and Tagliacozzo 2000; Tourunen 2008). We found that the morphological distinction between fennicus and tarandus ecotypes is expressed starkly in the difference in slenderness of the distal breadth-bone length, and minorly in the diaphysis breadth-bone length ratio. The ME3 is largely the same between sexes and is thus only ecotype indicative. Within this rule, the radioulna is an exception for the radioulnar ME3 slenderness is indicative of sex to a small degree in reindeer. In general, the ME3 and ME1 cannot make a clear distinction between the female wild and domestic tarandus varieties, though wild female tarandu*s* are slightly more slender than their domestic relatives. This endorses the morphological, and by extension genetic, as highlighted by other research, proximity of the wild and domestic tarandus varieties. However, when the indices are plotted against another variable, such as the GL of an element, clear sex and ecotype/variety dependent clusters form within the scatterplot. In application, individual specimens of unknown sex, castration status, and ecotype/variety, could be assigned to a specific group when falling clearly within one cluster.

Our very small sample of male wild mountain reindeer sometimes is fully separated from the male domestic tarandus group within the ME1 results and often groups together with small, castrated reindeer sub clusters. This makes us wonder whether these would actually be outliers if one were able to include a larger (or more reliable) sample size, since the wild tarandus females maintained largely the same ME1 slenderness as the domestic tarandus females. One point of assertion is that our included wild tarandus males could actually be sub-adult (see 4.2.4 and 4.2.5 for alternative interpretations).

In approaching collections in Fennoscandia for male wild tarandus bones to include in this study, we unfortunately came to learn that only very few institutions were in possession of male wild tarandus skeletons. The institutions that did have wild male tarandus bones usually only were in possession of mainly juvenile individuals (which showed signs of skeletal immaturity, i.e., unfused epiphyses, but which were sometimes marked down as ‘adult’). Of most individuals, age was unknown, and we included the bone specimens in our study that were fused. However, post-fusing growth, especially in younger individuals, is known to exist (Payne and Bull 1988; Davis 1996, 2000), and different bone elements fuse at different times (Hufthammer 1995; Takken Beijersbergen and Hufthammer 2012), so that the assessment of maturity between juvenile, sub-adult, and adult can not be fully reliable on the basis of only one or few bone elements of one individual. We can therefore not rule out that our male wild mountain reindeer were in fact sub-adult and therefore shows said patterns in our analyses. Indeed, such issue can not be ruled out for any osteological sample aged based on fusion of one or few elements and therefore one must always approach osteological analyses such as presented in this study with caution.

#### Simple variable combinations

Our research furthermore demonstrated that bone size visualized through simple variable combinations can be an effective means to indicate the presence of and differentiate between different sexes, ecotypes or varieties, and castrates. In general, it can be established that based on the presented size ranges, biometric totals (the iSizes of Mossiman) and the simple variable combinations that within our sample fennicus is the ecotype with the largest and longest bones, followed by domestic tarandus and lastly wild tarandus. The longer legs and larger body sizes of the fennicus ecotype has been well known and indicated in earlier studies (Nieminen and Helle 1980; Puputti and Niskanen 2008; Pelletier et al. 2020, 2021). Females are consistently smaller than males if of the same ecotype/variety and the two sexes are easily separated from each other if ecotype/variety is known, which is in line with the knowledge that reindeer are a sexually dimorphic species (e.g. Reimers et al. 1983; Weinstock 2000). The groups with greatest metric similarity are domestic castrates, domestic full males, wild mountain males, and Finnish forest females. The biometric likeness of female forest reindeer and male domestic reindeer had already been noted by other studies (Puputti and Niskanen 2008; Pelletier et al. 2020).

Metric indication and separation of sex and ecotypes/varieties through the use of simple variable combinations could be achieved in all long-bone elements, of which some of the most useful variable combinations are listed in Sect. "Biometric and statistical results" Results. Several simple variable combinations per element showed clear and separate cluster formations for male and female forest reindeer, domestic male and female tarandus, and wild female tarandus. What stands out in our simple variable combination scatterplots is the general trend of clustering between our groups. Male and female fennicus, as well as castrated reindeer show generally loose clustering patterns while full male and female tarandus groups demonstrate tighter clusters. This is in line with our calculations that male and female fennicus in general display more metric variance than the other groups, as well as castrated reindeer. Besides being more heterogenous when considered as a separate group, castration also enhances heterogeneity of the male group if the full males and castrates are considered together. This means that the heterogeneity of the male group can be used as an indication of castration if ecotype/variety is known. This type of proxy was used by Rannamäe et al. (2022), who studied the variation in male cattle metapodial measurements between different periods and found increased variance to be an indication of the rise of oxen culture. Similarly, high metric variance within a male or female group can also be used to indicate the presence of more than one ecotype or variety.

One of our main obstacles in castration cluster separation from simple variable combinations is that the best castration cluster separations are found if measurements are included that combine length measurements with other measurements. These can only be obtained from (near) complete long bones, which are a rare occurrence in the archaeological record. The results from our previous study (Van den Berg et al. 2023) indicated that separate proximal and distal parts could also be identified to castration status, if only domestic reindeer are included in the study. However, with the inclusion of other ecotypes and varieties, we find only useful cluster formations from (near) complete bone variable combinations, with indeed one exception (radioulnar CD x SDD). Two other obstacles in separating specifically castrates from the other groups is that clusters are rarely clear cut, and that male wild mountain reindeer are often (partially) grouped with the castrated subclusters. It remains unclear how this group will behave in cluster formation if more samples are included. Furthermore, the inconsistency we found in male wild tarandus clustering between elements and variables leads us to believe that these reindeer individuals might have been relatively young, as osteological maturation often advances unevenly between different bone elements and bone portions (e.g. Davis 2000). This, together with our results on slenderness within this group, lets us draw the conclusion that our small wild male tarandus sample might be an unreliable indication of size and shape of this reindeer group within our study.

#### Matters of size: nutrition and geographical origins

The relatively small bone sizes of the wild mountain reindeer, compared to domestic reindeer, is unexpected in part because Nieminen and Helle’s study on live measurements of domestic, wild mountain, and wild forest reindeer (1980) reported similar sizes between adult female wild mountain reindeer from Snøhetta, Norway, and domestic reindeer from different regions in Finland. This is not in line with our female wild mountain reindeer samples from Hardangervidda mountain plateau and Dovre/Rondane. Let us unpack this issue.

In Norway, three separate types of Norwegian wild mountain reindeer can be identified on the basis of their genetics and origin (Reimers and Colman 2006). These are original wild reindeer with minor genetic influx from nineteenth century reindeer herding (herds from Rondane, Snøhetta, and Sølnkletten), wild reindeer with influence of past reindeer herding activities in the area (animals from Hardangervida, Nordfjella, and Setesdal-Ryfylke), and feral reindeer from domestic origin (Flagstad and Røed 2003; Andersen and Hustad 2004; Røed et al. 2008, 2011; Reimers et al. 2012). This means that our samples come from the original wild reindeer and from wild reindeer admixed with domestic stock. Both herds have gone through profound genetic changes since ancient times. Rondane/Dovre reindeer have gone through major demographic fluctuation which resulted in loss and alteration of genetic variety, while Hardangervidda reindeer have suffered large-scale genetic introgression from a domestic gene pool (Røed et al. 2014). Statistical comparisons using iSize calculations between animals from these two different types tell us that the bone measurements are not statistically significantly different between the different locations within our sample (Table SI3). Within our study the different genetic compositions of the reindeer herds have thus not had measurable major size differences as a result directly within our sample.

Furthermore, Nieminen and Helle’s Snøhetta herd and our Rondane/Dovre herd essentially belong to the same genetic population. They both originate from the single Dovrefjell herd which previous to and during the nineteenth century wintered in the Rondane and Knutshø region and calved and summer pastured in the Snøhetta region to the west (Skogland and Mølmen 1980). Since then, habitat fragmentation due to intensified human use of the area has resulted in cessation of seasonal migration at the first half of the twentieth century (Skogland and Mølmen 1980; Skogland 1991) and the resulting different Snøhetta and Rondane/Knutshø subpopulations, the latter later dividing between separate Rondane and Knutshø herds (Skogland 1986). In this process the Rondane/Dovre wild reindeer population lost their best spring and calving areas and their best summer areas, residing now on the winter areas year-round (Bråtå 1995).

The Rondane/Dovre herd losing their best spring, calving, and summer areas is important for us to know because it gives us the clue that the surprisingly small size of our wild reindeer groups could be due to nutritional deficiency of the individuals in our studied sample. For reindeer, several works have illustrated that summer range conditions are most important for expression of size (Klein 1967; Movinckel and Prestbakmo 1969; Reimers 1980). Reindeer do not grow on lichens, one of their most important winter foods (Jacobsen et al. 1977, 1981), but indeed express their growth potential in summer (Klein 1967; Movinckel and Prestbakmo 1969; Reimers 1980). Different studies have presently been undertaken on the effect of nutrition on bone and teeth development, as well as body size in different mammal species. Zooarchaeological studies on sheep and goat (Davis 1996; Noddle 1974; Popkin et al. 2012) demonstrated that differences in nutrition have significant effect on bone development, with animals experiencing nutritional deficiency showing significantly poorer and delayed development and smaller bone structure.

At the same time, Reimers (unpublished data, in Reimers 1972) found that plane of nutrition has a bigger consequence in male than in female reindeer, because of male’s longer period of growth. Males don’t reach maturity until approximately 5 years of age, at which age they also fully participate in the rut (Skogland 1981). Females complete size development at approximately 3 years of age (Skogland 1983). This difference in temporal length of growing years between males and females could be an explanation of our study results as to why wild mountain males seem disproportionally smaller and more slender to male domestic reindeer than wild tarandus females to domestic females. This offers an alternative and reasonable explanation to the disparity in size proportional difference between the sexes.

However, our female wild tarandus individuals do come from different years (years 1965, 1970, and 1975) than our male wild tarandus (years 1950 and 1976), albeit from the same populations in Southern Norway. Skogland (1983) has pointed out through multiple lines of evidence that resource limitation has a major effect on reindeer size if compared to reindeer of the same stock whose nutritional needs had been met during their period of growth. More important for the issue at hand here, is that he has also shown that major size differences exist between reindeer shot in different years due to density dependent boom and crash cycles of reindeer populations and associated resource abundance or limitation. This is then also an argument for that the size difference between our wild mountain reindeer and Nieminen and Helle’s (1980) Snøhetta population could indeed stem from different harvesting years and differences in nutritional plane.

#### Matters of size: climate, domestication and breeding

There are more factors that suspectedly will have confounding influence on osteometric results. Body size changes of wild and domestic populations occur influenced by multiple independent factors. For example, Weinstock (1997) found significant size variability of Pleistocene reindeer between Northern and Southern Europe during the Late Glacial (ca. 13–10 ka B.P.). Reindeer populations in the warmer Southern regions were consistently smaller than their Northern correlates, a relation also found in other ungulates (Post et al. 1997; Martin et al. 2018). Body size reduction is also a prevalent trait observed in domestic species in the initial phases of domestication (Tchernov and Horwitz 1991; Zeder 2006; Zeder and Hesse 2000). However, the mechanism by which this is governed is still unclear and it is possible that it is an artifact of the general process of body size reduction that started at the end of the last Ice Age and has affected both wild and domestic ungulates (Zeder 2006, 2012, p 244). It remains yet uncertain how this applies exactly to reindeer.

However, domestic reindeer are indeed associated with body size changes. Analyses on archaeological bone assemblages from 12 to 17th-century Sámi offering sites for instance indicate a stark reduction in size between these past and modern domestic reindeer (Salmi et al. 2020). Likewise, in ethnographic work from the previous century in Finnish Sápmi, herders have reported that reindeer have undergone average size reductions during the herder’s lifetime: “Bulls often weighed over a hundred, but now, too, it is rare to find one over 70 [kg]. The reasons for such a sharp drop are many.” (Pitkänen et al. 1984, p 94). “Perhaps it’s to do with the food situation: reindeer there are many and the grazing gets used up. Or then again, maybe there’s been a mistake in the breeding methods used. The largest breeding bulls have been gelded to become pack and draught animals, which means the smaller bulls have taken over their breeding duties and produced smaller and smaller descendants.” (L. Leppäjärvi in Pitkänen et al. 1984, p 94).

In the literature about reindeer size in Fennoscandia we also find indications of the opposite: Skogland’s (1983) study shows that the body sizes of bucks of the wild tarandus herds of domestic origin fall within the upper ranges for wild reindeer in Norway. The exact reason for this topic is unknown but the attainment of larger reindeer sizes in domestic populations might stem from body size selection by herders. During interviews in Finland and Sápmi, present-day herders reported that smaller and weaker reindeer are always castrated or slaughtered in consideration of gene pool maintenance. The stronger and bigger bulls are kept for breeding. This also has connection to the reindeer meat industry, as bigger animals will give more meat yield per animal (Van den Berg 2025, in press).

#### Matters of size: interpretations from archaeological bone assemblages

Additionally, body mass and lifestyle differences between individuals within domestic populations lead to separate osteological stressors which affects bone shape and size (Puputti and Niskanen 2008; Niinimäki and Salmi 2016; Pelletier et al. 2020). This is then to say that the body size (changes) of reindeer and the relative proportional relationships in body size between reindeer populations from different times and different geographical origins in Fennoscandia is artifact of a complex set of mechanisms such as lifestyle, availability of resources, climate change, herder’s selection choices, among other factors. Our research furthermore demonstrates how the presence of different ecotypes and varieties, as well as populations supposedly subject to the abovementioned mechanisms, complicate result interpretation from osteological measurements.

This feature is aspect of an issue that many osteometric studies in archaeological and palaeontological method development deal with. It lies central to the question whether our found osteometric characteristics and differences between groups can be projected onto the past and thus onto archaeological reindeer bone assemblages. What follows is that the (absolute) size ranges as represented in our seven different groups might not be applicable to archaeological reindeer populations. We recommend applying our methods on archaeological bone assemblages critically and preferably on assemblages containing more individuals, and do not recommend using our methods for diagnostic purposes of single reindeer bone finds. When applied critically, we do believe that our methods are suitable for archaeological application and that relative size and slenderness can be powerful indicator to identify castration status, sex, and ecotype/variety.

We could not control for factors such as nutritional plane and body mass in our study as these characteristics of our included reindeer individuals was unknown, and lifestyle was largely unknown. Besides, taking these into account would have reduced our sample size beyond usefulness. The present study is furthermore limited by the number of skeletons included and their geographical and temporal origin. A study with a dataset that includes a wider range of reindeer skeletal specimens from different locations could clarify the variation in skeletal measurements between and within the domestic and wild reindeer populations. Such an analysis would allow for a more nuanced understanding of skeletal measurement ranges and bone size and may further complicate the differentiation between wild and domestic reindeer based on skeletal measurements. Our existing dataset does not adequately capture the full spectrum of metric diversity, which may lead to misinterpretation in comparative analyses. Future research is needed to address the knowledge gaps on osteological metric data in the context of ecological and anthropogenic influence.

## Conclusions

The identification of sex, ecotype/variety, and castration status in reindeer from ancient bone assemblages has the potential to advance our understanding of past human-reindeer relationships and interactions. This study presents osteometric analyses of 649 leg bones and pelvises of male and female wild forest reindeer, male and female wild mountain reindeer, and male (intact and castrated) and female domestic reindeer. We found slenderness, metric variability, and size to reflect ecotype (between fennicus and tarandus), variety (between wild and domestic tarandus), sex, and castration status in the reindeer individuals comprising our sample. In this paper we establish methods for distinguishing between the different group types that can be used on complete and fragmented reindeer bone finds. Our presented methods are easy to use, low-cost, and quick, and therefore easily accessible for zooarchaeological studies. In the case of sizable archaeological samples, the presence of males (intact and/or castrated) and females of domestic tarandus and wild tarandus and fennicus reindeer may be detected through the separate use or a combination of our outlined methods.

The data and analyses presented in this paper are an important progress in the knowledge of the osteometric similarities and differences between and within the reindeer populations currently extant in Fennoscandia. It furthermore advances our conception of the dimorphic properties of the species as well as that it highlights the potential prospect of developing similar approaches to identify castrated animals from bone assemblages comprised of different (wild vs. domestic) subspecies, ecotypes, or varieties to further our understanding of domestication histories of other animals. In addition, our results bring out the relevance of considering individual life histories, like castration and nutrition, in the interpretation of osteometric data. In extension, we have discussed the confounding effects of size changes within and between populations which are also applicable to other ungulate species studied by archaeologists. Considerable caution in the application of our methods should therefore be in place.

A natural direction for future research is the application of presented methods on ancient reindeer bone assemblages to establish the usefulness of the presented approaches. Especially when accompanied with contextual and molecular analysis this application can lead to the advancement of insight into past hunting and herding strategies, as well as other types of management. Our research results call for a more comprehensive exploration of the biometric characteristics of wild mountain reindeer bones, of especially males but also females, as well as of hybrids between fennicus and tarandus. The geographical proximity of the two ecotypes has led to a long history of interbreeding and shared morphological features (Nieminen and Helle 1980; Hakala 1997; Hakala et al. 1996; Puputti and Niskanen 2009; Pelletier et al. 2021), and present populations still occasionally interbreed (Nieminen and Helle 1980; Ministry of Agriculture and Forestry 2007; Milla Niemi 2023, personal communication). New studies on the size, shape, and allometry of these hybrid individuals would allow for a better understanding of osteometric variability in Fennoscandia and a refinement of zooarchaeological research on ancient reindeer bone assemblages.

## Supplementary Information

Below is the link to the electronic supplementary material.

Supplementary Information Database (XLSX 144 KB)

Supplementary Information Spreadsheet (XLSX 67 KB)

Supplementary Information (DOCX 46 KB)

Fig. SI1 Fig. SI1

High resolution image (TIFF 103111 KB)

Fig. SI2 Fig. SI2

High resolution image (TIFF 104408 KB)

Fig. SI3 Fig. SI3

High resolution image (TIFF 101623 KB)

Fig. SI4 Fig. SI4

High resolution image (TIFF 103462 KB)

Fig. SI5 Fig. SI5

High resolution image (TIFF 102522 KB)

Fig. SI6 Fig. SI6

High resolution image (TIFF 102577 KB)

Fig. SI7 Fig. SI7

High resolution image (TIFF 95573 KB)

Fig. SI8 Fig. SI8

High resolution image (TIFF 17579 KB)

Fig. SI9 Fig. SI9

High resolution image (TIFF 17579 KB)

Fig. SI10 Fig. SI10

High resolution image (TIFF 17579 KB)

Fig. SI11 Fig. SI11

High resolution image (TIFF 17579 KB)

Fig. SI12 Fig. SI12

High resolution image (TIFF 17579 KB)

Fig. SI13 Fig. SI13

High resolution image (TIFF 17579 KB)

## Data Availability

Availability of data and material: Code for statistical analysis is available upon request. Osteometric data is available in the Supplementary Information Database accompanying this paper. All plot results of simple variable combinations and Mennerich’s indices 1 and 3, as well as all results of the iSizes boxplots are available in our GitHub repository (https://github.com/mathildevandenberg/Wild-And-Domestic-Reindeer-Fennoscandia-Osteometrics/tree/main).

## Code availability

Code is available upon request.

## References

[CR1] Albarella U (1997) Shape variation of cattle metapodials: age, sex or breed? Some examples from medieval and postmedieval sites. Anthropozoologica 25(26):37–47

[CR2] Andersen R, Hustad H (2004) Villrein Samfunn. En veiledning til bevaring og bruk av Europas siste villreinfjell. Norsk institutt for naturforskning. NINA Temahefte 27

[CR3] Anderson DG, Harrault L, Milek KB, Forbes BC, Kuoppamaa M, Plekhanov AV (2019) Animal domestication in the high Arctic: Hunting and holding reindeer on the I͡Amal peninsula, northwest Siberia. J Anthropol Archaeol 55:101079. 10.1016/j.jaa.2019.101079

[CR4] Banfield AWF (1961) A revision of the reindeer and caribou, genus Rangifer. National Museum of Canada, Bulletin No. 177. Biological Series, p 66

[CR5] Bjørklund I (2013) Domestication, reindeer husbandry and the development of pastoralism. Acta Borealia 30(2):174–189. 10.1080/08003831.2013.847676

[CR6] Bjørnstad G, Flagstad Ø, Hufthammer AK, Røed KH (2012) Ancient DNA reveals a major genetic change during the transition from hunting to economy reindeer husbandry in northern Scandinavia. J Archaeol Sci 39(1):102–108

[CR7] Boessneck J, Müller HH, Teichert M (1964) Osteologische Unterscheidungsmerkmale zwischen Schaf (Ovis aries Linné) und Ziege (Capra hircus Linné). In: Kühn-Archiv: Arbeiten aus der Landwirtschaftlichen Fakultät der Martin-Luther-Universität Halle-Wittenberg (Vol. 78). Akademie-Verlag

[CR8] Bråtå HO (1995) Wild Reindeer and Planning in the Rondane Region. Reinventing the commons. Norwegian Institute for Urban and Regional Research (NIBR)

[CR9] Collinder B (1949) The Lapps. Princeton University Press

[CR10] Davis S (1996) Measurements of a group of adult female Shetland sheep skeletons from a single flock: a baseline for zooarchaeologists. J Archaeol Sci 23:593–612. 10.1006/jasc.1996.0056

[CR11] Davis S (2000) The effect of castration and age on the development of the Shetland sheep skeleton and a metric comparison between bones of males, females and castrates. J Archaeol Sci 27:373–390. 10.1006/jasc.1999.0452

[CR12] De Cupere B, Lentacker A, Van Neer W, Waelkens M, Verslype L (2000) Osteological evidence for the draught exploitation of cattle: first applications of a new methodology. Int J Osteoarchaeol 10(4):254–267

[CR13] Degteva A, Oskal A, Mathiesen SD, Burgess P, Aslaksen I, Johnsen KI, Magga A-M, van Rooij W (2017) Indigenous peoples’ perspectives. In: Arctic Monitoring and Assessment Programme (AMAP) (eds) Adaptation actions for a changing Arctic. Perspectives from the barents area, pp 167–194

[CR14] Von den Driesch A (1976) A guide to the measurement of animal bones from archaeological sites: as developed by the Institut für Palaeoanatomie, Domestikationsforschung und Geschichte der Tiermedizin of the University of Munich. Peabody Museum Bulletin 1. Peabody Museum of Archaeology and Ethnology, Harvard University. Peabody Museum Press

[CR15] Flagstad Ø, Røed KH (2003) Refugial origins of reindeer (Rangifer tarandus L.) inferred from mitochondrial DNA sequences. Evolution 57(3):658–670 12703955 10.1111/j.0014-3820.2003.tb01557.x

[CR16] Fock J (1966) Metrische Untersuchungen an Metapodien einiger europiiischer Rinderrassen. Dissertation, University of München

[CR17] Gordon BC (1990) World Rangifer communal hunting. In: Davis LB, Reeves BOK (eds) Hunters of the Recent Past. Unwin Hyman, London, pp 277–303

[CR18] Graves P (1995) Carl Linnaeus. The Lapland journey. Lockharton Press

[CR19] Guintard C (1996) Étude ostéométrique des métapodes de bovins: la race Charolaise. Dissertation, Paris National Museum of Natural History

[CR20] Guintard C, Lallemand M (2003) Osteometric study of metapodial bones in sheep (Ovis aries, L 1758). Ann Anat Anat Anz 185(6):573–583. 10.1016/S0940-9602(03)80131-0 10.1016/S0940-9602(03)80131-0 14704004

[CR21] Hakala A (1997) Origin and prehistory of the Fennoscandian reindeer with reference to the taxonomy and background in glacial Europe. Reports of the Early in the North Project. Hels Pap Archaeol 10:59–80

[CR22] Hakala A, Heikura K, Markovsky V, Bljudnik L, Pulliainen E, Danilov P (1996) On the taxonomy and geographical variation of the European reindeer with special reference to the wild forest reindeer, Rangifer tarandus fennicus Lönnberg 1909. Aquilo Ser Zool 29:3–23

[CR23] Hansen LI, Olsen B (2014) Hunters in Transition: An Outline of Early Sámi History. Brill

[CR24] Harcourt RA (1974) The dog in prehistoric and early historic Britain. J Archaeol Sci 1:151–175

[CR25] Harlin EK, Mannermaa K, Ukkonen P (2019) Animal bones from medieval and early modern Saami settlements in Finnish Lapland. In: Mannermaa K, Manninen MA, Pesonen P, Seppänen L (eds) Helsinki harvest: proceedings of the 11th Nordic Conference on the Application of Scientific Methods in Archaeology. Helsinki: The Archaeological Society of Finland, pp 149–177

[CR26] Heikura K, Pulliainen E, Danilov PI, Erkinaro E, Markovsky VA, Bljudnik L, Sulkava S, Lindgren E (1985) Wild forest reindeer, Rangifer tarandus fennicus L¨onnb., its historical and recent occurrence and distribution in Finland and the Karelian ASSR (USSR) with special reference to the development and movements of the Kuhmo (Finland) – Kamennojezero (USSR) subpopulation. Aquilo Ser Zool 23:22–45

[CR27] Helskog K, Indrelid S (2011) Humans and reindeer. Quatern Int 238:1–3. 10.1016/j.quaint.2011.03.018

[CR28] Hobday FTG (1914) Castration, including cryptorchids and caponing, and ovariotomy, 2nd edn. W and AK Johnston, Edinburgh and London

[CR29] Holand Ø, Horstkotte T, Kumpula J, Moen J (2022) Reindeer pastoralism in Fennoscandia. In: Horstkotte T, Holand Ø, Kumpula J, Moen J (eds) Reindeer husbandry and global environmental change. Taylor & Francis, pp 7–47

[CR30] Hufthammer AK (1995) Age determination of reindeer (Rangifer tarandus L.). Archaeozoologia VII(2):33–42

[CR31] Huntington H, Fox S (2005) The changing Arctic: indigenous perspectives. In: Symon C, Arris L, Heal B (eds) Arctic climate impact assessment. Cambridge University Press, pp 61–98

[CR32] Hvitfeldt E, Kuhn M (2023) Discrim: model wrappers for discriminant analysis. R package version 1.0.1. https://cran.r-project.org/web/packages/discrim/index.html. Accessed 16 June 2024

[CR33] Ingold T (1986) Reindeer economies: and the origins of pastoralism. Anthropol Today 2(4):5–10

[CR34] Jacobsen E, Bjarghov RS, Skjenneberg S (1977) Nutritional effects on weight gain and winter survival of reindeer calves. Sci Rep Agric Univ Norway 56(8):1–12

[CR35] Jacobsen E, Hove K, Bjarghov RS, Skjenneberg S (1981) Supplementary feeding of female reindeer on lichen diet during the last part of pregnancy. Acta Agriculturae Scandinavica 31:81–86

[CR36] Kennedy J, Baris C, Hoyland A, Selby PL, Freemont AJ, Braidman IP (1999) Immunofluorescent localization of estrogen receptor α in growth plates of rabbits, but not in rats, at sexual maturity. Bone 24:9–16 9916778 10.1016/s8756-3282(98)00148-3

[CR37] Klein DR (1967) Interactions of Rangifer tarandus (reindeer and caribou) with its habitat in Alaska. Finnish Game Res 30:289–293

[CR38] Kofinas G, Osherenko G, Klein D, Forbes B (2000) Research planning in the face of change: the human role in reindeer/caribou systems. Polar Res 19(1):3–21

[CR39] Kortesalmi JJ (2008) Poronhoidon synty ja kehitys Suomessa, Suomalaisen Kirjallisuuden Seura, Helsinki

[CR40] Krupnik I (1993) Arctic Adaptations. Whalers and Reindeer Herders of Northern Eurasia. University Press of New England, London

[CR41] Kuhn M, Wickham H, Tidymodels contributors (2020) Tidymodels: a collection of packages for modeling and machine learning using tidyverse principles. https://www.tidymodels.org. Accessed 7 Oct 2023

[CR42] Losey RJ, Nomokonova T, Arzyutov DV, Gusev AV, Plekhanov AV, Fedorova NV, Anderson DG (2020) Domestication as enskilment: harnessing reindeer in Arctic Siberia. J Archaeol Method Theory 28:197–231

[CR43] Martin JM, Mead JI, Barboza PS (2018) Bison body size and climate change. Ecol Evol 8(9):4564–4574 29760897 10.1002/ece3.4019 PMC5938452

[CR44] Mazzorin JG, Tagliacozzo A (2000) Morphological and osteological changes in the dog from the Neolithic to the Roman period in Italy. In: Crockford SJ (ed), Dogs Through Time: An Archaeological Perspective, pp 141–161. BAR International Series 889

[CR45] Mennerich G (1968) Römerzeitliche Tierknochen aus drei Fundorten des Niederrheingebietes. Doctoral Dissertation, Institut für Paläoanatomie, domestikationsforschung und Geschichte der Tiermedizin der Universität München, München

[CR46] Ministry of Agriculture and Forestry (2007) Management plan for the wild forest reindeer population in Finland. Vammalan Kirjapaino Oy

[CR47] Montonen M (1974) Suomen peura. WSOY, Porvoo

[CR48] Mosimann JE (1970) Size allometry: size and shape variables with characterizations of the lognormal and generalized gamma distributions. J Am Stat Assoc 65(330):930–945

[CR49] Movinckel H, Prestbakmo H (1969) Variasjon i slaktevekt hos rein i en del sommerbeitedistrikt i Finnmark og Troms. Sci Rep Agric Coll Nor 58:1–26

[CR50] Nieminen M (1977) Poron alkuperä. Suomen Luonto 36: 93-97

[CR51] Nieminen M (1980) The evolution and taxonomy of the genus Rangifer in northern Europe. Proceedings Second International Reindeer and Caribou Symposium. Røros, Norway 17–21.09.1979

[CR52] Nieminen M (1982a) Metsäpeura – suomenpeura. In: Veijonen R (ed) Metsäpeuran paluu. Kainuun Sanomat Kirjapaino Oy, pp 4–11

[CR53] Nieminen M (1982b) Metsäpeuran menneisyys Suomenselällä. In: Veijonen R (ed) Metsäpeuran paluu. Kainuun Sanomat Kirjapaino Oy, pp 19–21.

[CR54] Nieminen M, Helle T (1980) Variations in body measurements of wild and semi-domestic reindeer (Rangifer tarandus) in Fennoscandia. Ann Zool Fenn 17:275–283

[CR55] Niinimäki S, Salmi A-K (2016) Entheseal changes in free-ranging versus zoo reindeer – observing activity status of reindeer. Int J Osteoarchaeol 26:314–323

[CR56] Niinimäki S, Härkönen L, Puolakka H-L, Van den Berg M, Salmi A-K (2021) Cross-sectional properties of reindeer long bones and metapodials allow identification of activity patterns. Archaeol Anthropol Sci 13:146

[CR57] Noddle B (1974) Ages of epiphyseal closure in feral and domestic goats and ages of dental eruption. J Archaeol Sci 1(2):195–204

[CR58] Paine R (1994) Herds of the tundra: a portrait of Saami reindeer pastoralism. Smithsonian Institution Press

[CR59] Paliskuntain yhdistys (2024) Paliskunnat. https://paliskunnat.fi/py/paliskunnat/. Accessed 06 Feb 2024

[CR60] Payne S, Bull G (1988) Component of variations in measurements of pig bones and teeth, and the use of measurements to distinguish wild from domestic pigs. Archaeozoologia 2:27–66

[CR61] Pelletier M, Kotiaho A, Niinimäki S, Salmi A-K (2020) Identifying early stages of reindeer domestication in the archaeological record: A 3D morphological investigation on forelimb bones of modern populations from Fennoscandia. Archaeol Anthropol Sci 12:169 32704330 10.1007/s12520-020-01123-0 PMC7366605

[CR62] Pelletier M, Kotiaho A, Niinimäki S, Salmi A-K (2021) Impact of selection and domestication on hindlimb bones of modern reindeer populations: Archaeological implications for early reindeer management by Sámi in Fennoscandia. Hist Biol 34(5):802–820

[CR63] Pitkänen MA, Pitkänen I, Hayhurst R (1984) Poromiehet: the Lapps and their reindeer. Weilin ja Göös, Espoo

[CR64] Popkin PR, Baker P, Worley F, Payne S, Hammon A (2012) The sheep project (1): determining skeletal growth, timing of epiphyseal fusion and morphometric variation in unimproved Shetland sheep of known age, sex, castration status and nutrition. J Archaeol Sci 39(6):1775–1792

[CR65] Post E, Stenseth NC, Langvatn R, Fromentin JM (1997) Global climate change and phenotypic variation among red deer cohorts. Proc R Soc Lond B Biol Sci 264(1386):1317–1324 10.1098/rspb.1997.0182 PMC1688584 9332016

[CR66] Pulliainen E, Leinonen A (1990) Petra. Karjalan peura. Tammi, Helsinki

[CR67] Puputti A-K, Niskanen M (2008) The estimation of body weight of the reindeer (Rangifer tarandus L.) from skeletal measurements: preliminary analyses and application to archaeological material from 17th- and 18th-century northern Finland. Environ Archaeol 13(2):153–164. 10.1179/174963108X343272

[CR68] Puputti A-K, Niskanen M (2009) Identification of semi-domesticated reindeer (Rangifer tarandus tarandus, Linnaeus 1758) and wild forest reindeer (Rt fennicus, Lönnberg 1909) from postcranial skeletal measurements. Mamm Biol 74:49–58

[CR69] R Core Team (2023) R: A Language and Environment of Statistical Computing. Foundation for Statistical Computing, Vienna, Austria. https://www.R-project.org/. Accessed 9 Sept 2023

[CR70] Rannamäe E, Saarma U, Bläuer A (2022) Cultural influences on the castration age of cattle in the northern Baltic Sea region during the medieval and post-medieval periods. J Archaeol Sci 137:105517

[CR71] Reimers E (1972) Growth in domestic and wild reindeer in Norway. J Wildl Manage 612–619

[CR72] Reimers E (1980) Activity pattern: The major determinant for growth and fattening in Rangifer? In: Reimers E, Gaare E, Skjenneberg S (eds), Reindeer/Caribou Symposium II Norway 1979, pp 466-474. Direktoratet for Vilt og Ferskvannsfisk

[CR73] Reimers E (2007) Villrein i Norge; Populasjonsøkologi, forvaltning og jakt. Rangifer Rep 12:35–45

[CR74] Reimers E, Colman JE (2006) Reindeer and caribou (Rangifer tarandus) response towards human activities. Rangifer 26(2):55–71

[CR75] Reimers E, Klein DR, Sorumgard R (1983) Calving time, growth rate, and body size of Norwegian reindeer on different ranges. Arct Alp Res 15(1):107–118. 10.2307/1550986

[CR76] Reimers E, Røed KH, Colman JE (2012) Persistence of vigilance and flight response behaviour in wild reindeer with varying domestic ancestry. J Evol Biol 25:1543–1554 22587024 10.1111/j.1420-9101.2012.02538.x

[CR77] Røed KH, Flagstad Ø, Nieminen M, Holand Ø, Dwyer MJ, Røv N, Vilà C (2008) Genetic analyses reveal independent domestication origins of Eurasian reindeer. Proc R Soc Lond B Biol Sci 275:1849–1855. 10.1098/rspb.2008.0332 10.1098/rspb.2008.0332 PMC2593925 18460427

[CR78] Røed KH, Flagstad Ø, Bjørnstad G, Hufthammer AK (2011) Elucidating the ancestry of domestic reindeer from ancient DNA approaches. Quatern Int 238:83–88. 10.1016/j.quaint.2010.07.031

[CR79] Røed KH, Bjørnstad G, Flagstad Ø, Haanes H, Hufthammer AK, Jordhøy P, Rosvold J (2014) Ancient DNA reveals prehistoric habitat fragmentation and recent domestic introgression into native wild reindeer. Conserv Genet 15(5):1137–1149

[CR80] Røed KH, Bjørklund I, Olsen BJ (2018) From wild to domestic reindeer – genetic evidence of a non-native origin of reindeer pastoralism in northern Fennoscandia. J Archaeol Sci Rep 19:279–286. 10.1016/j.jasrep.2018.02.048

[CR81] Røed KH, Kvie KS, Bårdsen B-J, Laaksonen S, Lohi H, Kumpula J, Aronsson K-Å, Åhman B, Våge J, Holand Ø (2021) Historical and social- cultural processes as drivers for genetic structure in Nordic domestic reindeer. Ecol Evol 11:8910–8922

[CR82] Røed KH, Kvie KS, Bårdsen BJ (2022) Genetic structure and origin of semi-domesticated reindeer. In: Horstkotte T, Holand O, Kumpula J, Moen J (eds) Reindeer Husbandry and Global Environmental Change: Pastoralism in Fennoscandia, pp 48-60. Routledge. 10.4324/9781003118565-4

[CR83] Rönnow C (1949) Om kasterring hos de kenskötande folken med särskild hänsyn till rennomadismen I Sverige. Folk Liv XII-XIII:141–162

[CR84] Roué M (2012) En Samisk renskötares kulturlandskap: minnet, sinnena och de etiska principerna. In: Raasakka N, Sivonen S (eds) Nordliga landskap: “Tillämpning av den europeiska landskapskonventionen i kommunerna på Nordkalotten”-Conference 7.-9.9. 2011, Enare, Finland. Närings-, trafik- och miljöcentralen I Lappland, Rapporter 48. Lapin ELY-keskus, Kopijyvä Oy, pp 46–51

[CR85] Salmi A-K (2023) The archaeology of reindeer domestication and herding practices in Northern Fennoscandia. J Archaeol Res 31(4):617–660

[CR86] Salmi A-K, Fjellström M, Äikäs T, Spangen M, Núñez M, Lidén K (2020) Zooarchaeological and stable isotope evidence of Sámi reindeer offerings. J Archaeol Sci Rep 29:102129. 10.1016/j.jasrep.2019.102129

[CR87] Salmi A-K, van den Berg M, Niinimäki S, Pelletier M (2021) Earliest archaeological evidence for domesticated reindeer economy among the Sámi of Northeastern Fennoscandia AD 1300 onwards. J Anthropol Archaeol 62:101303

[CR88] Sametinget (2024) Karta över Sveriges samebyar. https://www.sametinget.se/8382. Accessed 06 Feb 2024

[CR89] Sampson P, Siegel A (1985) The Measure of “Size” Independent of “Shape” for Multivariate Lognormal Populations. J Am Stat Assoc 80(392):910–914

[CR90] Schild U (1962) Metrische Untersuchungen an Metararpal- und Metatarsalknochen gesunder Rinder, als Grundlage für die pathologisch-anatomische Beurteilung der Hauptmittelfussknochen. Doctoral dissertation, University of Zürich

[CR91] Siivonen L (1975) New results on the history and taxonomy of the mountain, forest and domestic reindeer in Northern Europe. Biological Papers of the University of Alaska Special Report 1: 342-354

[CR92] Silberberg M, Silberberg R (1971) Steroid hormones and bone. In: Bourne GH (ed), The biochemistry and physiology of bone (Vol III, 2nd ed, pp 401–484). Academic Press

[CR93] Silver IA (1963) The ageing of domestic animals. In: Science in archaeology: A comprehensive survey of progress and research (pp 250–268). Thames and Hudson

[CR94] Skjenneberg S, Slagsvold L (1979) Reindeer husbandry and its ecological principles. US Department of the Interior, Bureau of Indian Affairs

[CR95] Skogland T (1981) Comparative social organization. In: Bliss LC, Cragg JB, Heal DW, Moore JJ (eds), Tundra ecosystems: A comparative analysis. International Biological Programme 25, pp 452–483. Cambridge University Press

[CR96] Skogland T (1983) The effects of density dependent resource limitation on size of wild reindeer. Oecologia 60:156–168 28310482 10.1007/BF00379517

[CR97] Skogland T (1986) Density dependent food limitation and maximal production in wild reindeer herds. J Wildl Manag 50(2):314–319

[CR98] Skogland T (1991) What are the effects of predators on large ungulate populations? Oikos 61:401–411

[CR99] Skogland T, Mølmen Ø (1980) Prehistoric and present habitat distribution of wild mountain reindeer at Dovrefjell. In: Reimers E, Gaare E, Skjenneberg S (eds) Reindeer/Caribou Symposium II Norway 1979. Direktoratet for Vilt-og Ferskvannsfisk, Trondheim, pp 130–141

[CR100] Soppela P, van den Berg M, Kynkäänniemi SM, Wallén H (2022) Castration as part of reindeer herd management. In: Salmi A (ed) Domestication in Action: Past and Present Human-Reindeer Interaction in Northern Fennoscandia. Springer International Publishing, pp 65–94

[CR101] Spencer A (1978) This chancing world, the Lapps. Crane, Russak and Company, New York

[CR102] Storli I (1993) Sámi Viking age pastoralism—or the fur trade paradigm reconsidered. Nor Archaeol Rev 26(1):1–20. 10.1080/00293652.1993.9965550

[CR103] Svestad A (2018) Entering other realms: Sámi burials in natural rock cavities and caves in northern Fenno-Scandinavia between 900 BC and AD 1700. In: Caves and Ritual in Medieval Europe, AD 500, 1500, pp 13–31. Oxbow Books

[CR104] Takken Beijersbergen LM, Hufthammer AK (2012) Age determination of reindeer (Rangifer tarandus) based on postcranial elements. Groningen Archaeological Studies, 21: 11-20

[CR105] Tchernov E, Horwitz LK (1991) Body size diminution under domestication: unconscious selection in primeval domesticates. J Anthropol Archaeol 10(1):54–75

[CR106] Telldahl Y, Svensson EM, Götherström A, Storå J (2012) Osteometric and molecular sexing of cattle metapodia. J Archaeol Sci 39(1):121–127. 10.1016/j.jas.2011.09.009

[CR107] Tourunen A (2008) Animals in an Urban Context. A Zooarchaeological study of the Medieval and Post-Medieval town of Turku. Academic Dissertation. Turun Yliopisto, Turku

[CR108] van den Berg M, Wallen H, Salmi A-K (2023) The osteometric identification of castrated reindeer (Rangifer tarandus) and the significance of castration in tracing human-animal relationships in the North. Archaeol Anthropol Sci 15(1):3 36514485 10.1007/s12520-022-01696-y PMC9734228

[CR109] Vigne JD (2015) Early domestication and farming: what should we know or do for a better understanding? Anthropozoologica 50(2):123–150

[CR110] Norsk Villreinsenter (2024) Villreinområder i Norge. https://villrein.no/villreinomrader/. Accessed 06 Feb 2024

[CR111] Van den Berg M (2025) Reindeer (Rangifer tarandus) castration in Fennoscandia: domestication theory, archaeological methods, and interpretive perspectives. J Anthropol Archaeol (in press)

[CR112] Vitebsky P (2005) The reindeer people: living with animals and spirits in Siberia. Houghton Mifflin Harcourt

[CR113] Vorren Ø (1973) Some Trends of the Transition from Hunting to Nomadic Economy in Finnmark. In: Berg G (ed), Circumpolar Problems. Habitat, Economy, and Social Relations in the Arctic. A Symposium for Anthropological Research in the North, September 1969, pp 185–194. Oxford: Pergamon Press

[CR114] Weinstock J (1997) The relationship between body size and environment: the case of late pleistocene reindeer (Rangifer tarandus). Archaeofauna 6:123–135

[CR115] Weinstock J (2000) Osteometry as a source of refined demographic information: sex-ratios of reindeer, hunting strategies, and herd control in the late glacial site of Stellmoor, Northern Germany. J Archaeol Sci 27(12):1187–1195. 10.1006/jasc.1999.0542

[CR116] Zeder MA (2006) Archaeological approaches to documenting animal domestication. In: Zeder MA, Bradley DG, Emshwiller E, Smith BD (eds) Documenting domestication: new genetic and archaeological paradigms. University of California Press, pp 171–180

[CR117] Zeder MA, Hesse B (2000) The initial domestication of goats (Capra hircus) in the Zagros Mountains 10,000 years ago. Science 287:2254–2257 10731145 10.1126/science.287.5461.2254

[CR118] Zeder MA (2012) Pathways to animal domestication. Biodiversity in agriculture: domestication, evolution, and sustainability, 10: 227-259

