# Supplementary material for: Osteometric distinctions between domestic reindeer (Rangifer tarandus tarandus), wild mountain reindeer (R.t.t.), wild forest reindeer (R.t. fennicus), and the identification of castrated reindeer bones: Biometric explorations and archaeological methods
Source: Archaeol Anthropol Sci. 2025 Apr 1;17(4):94. doi: 10.1007/s12520-025-02198-3 (PMC11961526; doi:10.1007/s12520-025-02198-3)
Supplement: Supplementary file 3 — Supplementary Information (DOCX 46 KB) [file 12520_2025_2198_MOESM3_ESM.docx]

**Supplementary Information**

Article title: Osteometric distinctions between domestic reindeer (*Rangifer tarandus tarandus*), wild mountain reindeer (*R.t.t*.), wild forest reindeer (*R.t. fennicus*), and the identification of castrated reindeer bones: Biometric explorations and archaeological methods

Journal name: Journal of Archaeological and Anthropological Sciences

Authors: Mathilde van den Berg¹, Henri Wallen²

¹[Mathilde.vandenberg@oulu.fi](mailto:Mathilde.vandenberg@oulu.fi) (Corresponding author) Archaeology, History, Culture and Communication Studies, Faculty of Humanities, University of Oulu, Oulu, Finland

Orcid: <https://orcid.org/0000-0002-8087-7552>

²Henri.wallen@ulapland.fi Arctic Centre, University of Lapland, Rovaniemi, Finland.

Orcid: <https://orcid.org/0000-0002-8584-8398>

**Table SI1** Measurement definitions (Mm=measurement), with used measuring device, metric, original reference to the measurement, and original name (after Van den Berg et al. 2023, Table 2, reproduced with permission).

| **Element** | **Mm** | **Definition** | **Metric** | **Device** | **Reference** | **Original name** |
| --- | --- | --- | --- | --- | --- | --- |
| Humerus | GL | Greatest length. The long axis must lie parallel to the supporting surface. Measured from the intermedial tubercle's most proximal projection to the distal end's most distal projection. | cm | Measuring box | Von den Driesch, 1976 |  |
| Humerus | GLC | Greatest length measured from the humeral caput, measured from the proximal flat surface of the caput to the most distal end of the humerus, parallel to the longitudinal axis. | cm | Big calipers | Von den Driesch, 1977 |  |
| Humerus | GLI | Greatest length lateral. Measured from the most proximal projection of the greater (lateral) tubercle to the most distal point of the middle ridge of the trochlea, in parallel with the longitudinal axis. | cm | Big calipers | Von den Driesch, 1978 |  |
| Humerus | Bp | Greatest breadth of the proximal end. Measured from the most medial projection of the minor (medial) tubercle to the most lateral projection of the greater (lateral) tubercle. | cm | Measuring box | Von den Driesch, 1979 |  |
| Humerus | SD | Smallest breadth of the diaphysis. Smallest mediolateral diameter of the diaphysis. | mm | Small calipers | Von den Driesch, 1980 |  |
| Humerus | CD | Smallest circumference of the diaphysis. Measured perpendicular to the longitudinal axis. | cm | Tape measure | This study |  |
| Humerus | Bd | Greatest breadth of the distal end. Greatest breadth from the most medial projection of the medial trochlea, beyond the direct articular surface, to the most lateral projection of the lateral trochlea, not including the lateral tubercle. | cm | Measuring box | Von den Driesch, 1976 |  |
| Humerus | BT | Greatest breadth of the trochlea. Measured from the most lateral point of the lateral trochlea to the most medial point of the medial trochlea, at the point of the most cranial projection, measured not at a right angle to the longitudinal axis of the humerus but to the imagined mediolateral axis of the trochlea. | mm | Small calipers | Von den Driesch, 1976 |  |
| Humerus | HT | Height of the trochlea. The height of the medial trochlear condyle is measured not at a right angle to the longitudinal axis of the humerus but to the imagined mediolateral axis of the trochlea. | mm | Small calipers | Puputti & Niskanen, 2008 | HUM TH |
| Humerus | DC | Anterior-posterior depth of the caput. Measured from the most cranial point of the flat articular surface of the humeral caput to the most caudal point of the caudal-distal rim of the caput. | mm | Small calipers | Puputti & Niskanen, 2008 | HUM HHAP |
| Humerus | HTC | Height trochlear constriction. Vertical diameter of the central trochlear constriction, as parallel to the longitudinal axis of the humerus. | mm | Small calipers | Davis, 1996 |  |
| Humerus | Dp | Greatest depth of the proximal end. Anterior-posterior depth from the most caudal projection of the humeral caput to the most cranial projection of the most distal bulge of the greater (lateral) tubercle, measured perpendicular to the longitudinal axis of the humerus. | cm | Measuring box | Von den Driesch, 1976 |  |
| Humerus | Dd | Greatest depth of the distal end. Anterior-posterior depth from the most caudal projection of the medial epicondyle to the most cranial point of the medial trochlea. Dd is defined differently by Weinstock 2000a. | mm | Small calipers | Van den Berg et al. 2023 |  |
| Humerus | PL | Physiological length. Measured parallel to the longitudinal axis of the humerus from the most distal projection of the flat humeral articular facet to the central constriction of the trochlea. | cm | Big calipers | Van den Berg et al. 2023 |  |
| Radioulna | GL | Greatest length of the radioulna. Measured from the most proximal projection of the tubercle of the olecranon to the most distal projection of the styloid process. Measured parallel to the longitudinal axis. | cm | Measuring box | Von den Driesch, 1976 |  |
| Radioulna | PL | Physiological length of the radius. The longitudinal axis measured from the middle of the medial ridge of the proximal radial articular surface to the distal ridge between the articular surfaces of the carpal bones. | cm | Curved calipers | Niinimäki et al., 2021 | LI |
| Radioulna | Bp | Breadth of the proximal end of the radius. Measured from the most lateral process to the most medial process of the proximal radius, i.e., including muscle attachments. | mm | Small calipers | Von den Driesch, 1977 |  |
| Radioulna | BFp | Greatest breadth of the proximal articular facet of the radius. Measured in the same plane as the Bp. | mm | Small calipers | Von den Driesch, 1978 |  |
| Radioulna | CD | Smallest circumference of the diaphysis of the radioulna. Measured perpendicular to the longitudinal axis. | cm | Tape measure | Von den Driesch, 1979 |  |
| Radioulna | Bd | Greatest breadth of the distal end of the radioulna. Measured from the most medial process of the distal radius to the most lateral point of the ulnar styloid process. | mm | Small calipers | Van den Berg et al. 2023 |  |
| Radioulna | SD | Smallest breadth of the radial diaphysis. Smallest mediolateral diameter, excluding the ulna. | mm | Small calipers | Von den Driesch, 1979 |  |
| Radioulna | SDD | Smallest depth of the radial diaphysis. Usually measured dolsar-palmar at the height of the antebrachial interosseous space. | mm | Small calipers | Von den Driesch, 1979 |  |
| Radioulna | Dd | Depth of the distal end. Measured from the most dorsal projections of the radial medial and lateral tendon attachments to the most palmar projection of the distal radial, above the radial articular facets. | mm | Small calipers | Weinstock, 2000a |  |
| Radioulna | Dp | Depth of the proximal end. Depth of the medial articular facet. | mm | Small calipers | Puputti & Niskanen, 2009 | RAD PRAP |
| Radioulna | LO | Length of the olecranon. Measured from the most proximal projection of the tubercle olecranon to the most distal notch of the processus anconeus (or most dorsal notch of the trochlear incisura), measured in parallel to the olecranon's "own" imaginary axis. | mm | Small calipers | Von den Driesch, 1976 |  |
| Radioulna | SDO | Smallest depth of the olecranon. Measured from the most dented point on the dorsal ridge to the palmar ridge of the olecranon. | mm | Small calipers | Von den Driesch, 1976 |  |
| Metacarpus | GL | Greatest length. Measured in the longitudinal axis from the most proximal projection of the articular surface to the most distal projection of the verticuli of the trochlei. | cm | Measuring box | Von den Driesch, 1976 |  |
| Metacarpus | Bp | Breadth proximal. Maximum medial-lateral diameter of the proximal end, including the muscle attachments. | mm | Small calipers | Von den Driesch, 1976 |  |
| Metacarpus | Dp | Depth proximal. Maximum dorsal-palmar diameter of the proximal end, including the muscle attachments. | mm | Small calipers | Von den Driesch, 1976 |  |
| Metacarpus | SD | Smallest breadth of the diaphysis. Smallest mediolateral diameter of the diaphysis. | mm | Small calipers | Schild, 1962; Von den Driesch, 1976 | |
| Metacarpus | CD | Smallest circumference of the diaphysis. Measured perpendicular to the longitudinal axis. | cm | Tape measure | Von den Driesch, 1976 |  |
| Metacarpus | Bd | Breadth distal. The maximum medial-lateral diameter of the distal end is measured from the most lateral projections of the medial and lateral epicondyles. Perpendicular to the longitudinal axis. | mm | Small calipers | Von den Driesch, 1976 |  |
| Metacarpus | BTm | Breadth of the medial trochlea. Greatest medial-lateral diameter of the medial trochlea, perpendicular to the longitudinal axis. | mm | Small calipers | Telldahl et al., 2012 | BFdm |
| Metacarpus | BTl | Breadth of the lateral trochlea. Greatest medial-lateral diameter of the lateral trochlea, perpendicular to the longitudinal axis. | mm | Small calipers | Telldahl et al., 2013 | BFdl |
| Metacarpus | DVm | Depth of the medial verticulus. Greatest dorsal-palmar diameter of the medial verticulus. | mm | Small calipers | Davis 1996; SGWP in Popkin et al., 2012 | DVM |
| Metacarpus | DVl | Depth of the lateral verticulus. Greatest dorsal-palmar diameter of the lateral verticulus. | mm | Small calipers | Davis 1996; SGWP in Popkin et al., 2012 | DVL |
| Metacarpus | BAp | Breadth of the proximal articular surface. Greatest medial-lateral diameter of the proximal articular facet, perpendicular to the longitudinal axis. | mm | Small calipers | SGWP in Popkin et al., 2012 | BFP |
| Metacarpus | BDF | Breadth of the diaphysis along the distal line of fusion. Greatest medial-lateral diameter of the fusion site of the distal end. | mm | Small calipers | Popkin et al., 2012 | BdFus |
| Metacarpus | BA | Breadth between the articular crests. Measured between the most distal points of the crests. | mm | Small calipers | Telldahl et al., 2012 | Bcr |
| Metacarpus | GCD | Greatest circumference of the diaphysis. | cm | Tape measure | Van den Berg et al. 2023 |  |
| Metacarpus | GDD | Greatest depth of the diaphysis. Measured perpendicular to the longitudinal axis. | mm | Small calipers | Van den Berg et al. 2023 |  |
| Metacarpus | SDD | Smallest depth of the diaphysis. Smallest dorsal-palmar diameter of the diaphysis. | mm | Small calipers | Van den Berg et al. 2023 |  |
| Metacarpus | PL | Physiological length. Measured from the most distal point of the proximal medial articular surface to the most distal projection of the medial epicondyle of the medial trochlea. | cm | Curved calipers | Van den Berg et al. 2023 |  |
| Metacarpus | DFp | Depth of the proximal articular facet. Measured from the medial facet, not perpendicular to the longitudinal axis but as shown in Fig. 5e. | mm | Small calipers | Van den Berg et al. 2023 |  |
| Femur | GL | Greatest length. Measured from the most proximal projection of the major trochanter to the most distal projection of the trochlear ridge(s) and/or condyle(s) parallel to the longitudinal axis of the femur. The bone must not lie flat on the measuring board but with its proximal end slightly raised. | cm | Measuring box | Von den Driesch, 1976 |  |
| Femur | GLC | Greatest length measured from the most proximal point of femoral caput to the most distal projection of the trochlear ridge(s) and/or condyle(s), parallel to the longitudinal axis of the femur. | cm | Measuring box | Von den Driesch, 1976 |  |
| Femur | Bp | Breadth of the proximal end. Measured from the most lateral projection of the major trochanter to the most medial point of the femoral caput. | mm | Small calipers | Von den Driesch, 1976 |  |
| Femur | DC | Depth of the femoral caput. Greatest diameter of the femoral caput. | mm | Small calipers | Von den Driesch, 1976 |  |
| Femur | SD | Smallest breadth of the diaphysis. Smallest mediolateral diameter of the diaphysis. | mm | Small calipers | Von den Driesch, 1976 |  |
| Femur | SDD | Smallest depth of the diaphysis. Smallest cranial-caudal diameter of the diaphysis. | mm | Small calipers | This study |  |
| Femur | CD | Smallest circumference of the diaphysis. Measured perpendicular to the longitudinal axis. | cm | Tape measure | Von den Driesch, 1976 |  |
| Femur | Bd | Breadth of the distal end. Measured from the most lateral projection of the lateral epicondyle to the most medial projection of the medial condyle or epicondyle, depending on which is broader, perpendicularly to the longitudinal axis of the femur. | mm | Small calipers | Von den Driesch, 1976 |  |
| Femur | Dd | Depth of the distal end. Greatest cranial-caudal diameter measured from the most cranial projection of the medial trochlear ridge to the most caudal projection of the medial condyle, perpendicular to the long axis. | cm | Measuring box | Weinstock, 1997 |  |
| Femur | BT | Breadth of the trochlea. Measured horizontally between the trochlea's medial and lateral ridges, at the trochlea's most proximal end. BT is Defined differently by Weinstock 2000a. | mm | Small calipers | Van den Berg et al. 2023 |  |
| Femur | PL | Physiological length. Measured between the most proximal point of the femoral caput to the most distal articular surface of the medial condyle, parallel to the longitudinal axis. | cm | Big calipers | Niinimäki et al., 2021 | LI |
| Tibia | GL | Greatest length. Measured between the most proximal point(s) of the intercondylar tubercle(s) and the most distal projection of the medial malleolus, parallel to the longitudinal axis. | cm | Measuring box | Von den Driesch, 1976 |  |
| Tibia | Ll | Length lateral. Measured between the most lateral and proximal part of the tibular plateau and the most distal projection of the lateral malleolar process, parallel to the longitudinal axis. | cm | Big calipers | Von den Driesch, 1976 |  |
| Tibia | SD | Smallest breadth of the diaphysis. Smallest mediolateral diameter of the diaphysis. | mm | Small calipers | Von den Driesch, 1976 |  |
| Tibia | SDD | Smallest depth of the diaphysis. Smallest dorsal-plantar diameter of the diaphysis. | mm | Small calipers | This study |  |
| Tibia | CD | Smallest circumference of the diaphysis. Measured perpendicular to the longitudinal axis. | cm | Tape measure | Von den Driesch, 1976 |  |
| Tibia | Bd | Breadth distal. Measured between the most medial projection of the medial malleolus and most lateral projection of the lateral malleolus, perpendicular to the dorsal-plantar axis of the direction of the distal articular grooves. | mm | Small calipers | Von den Driesch, 1976 |  |
| Tibia | Dd | Depth distal. Measured between the most dorsal projection of the distal tibia and the most plantar projection of the distal tibia, parallel to the dorsal-plantar axis of the direction of the distal articular grooves. | mm | Small calipers | Von den Driesch, 1976 |  |
| Tibia | Dp | Depth proximal. Measured between the proximal-most dorsal point of the tibial tuberosity and the most plantar projections of the medial and/or lateral tibial plateau(s) perpendicular to the longitudinal axis of the tibia. | cm | Measuring box | Puputti & Niskanen, 2009 | TIB PTAP |
| Tibia | PL | Physiological length. Greatest length between the central intercondylar area of the proximal end to the mid-ridge of the distal articular facet, parallel to the longitudinal axis. | cm | Curved calipers | Niinimäki et al., 2021 | LI |
| Tibia | BFp | Breadth of the proximal articular facet. Measured between the most medial point of the medial articular surface to the most lateral point of the lateral articular surface, perpendicular to the longitudinal axis of the tibia. | mm | Small calipers | Niinimäki et al., 2021 |  |
| Tibia | BFd | Breadth of the distal articular facet. The breadth of the articular facet, which articulates with the talus; the malleolar articular facet, is not included in the measurement. Measured perpendicular to the dorsal-plantar axis of the direction of the distal articular grooves. | mm | Small calipers | Van den Berg et al. 2023 |  |
| Metatarsus | GL | Greatest length. Measured in the longitudinal axis from the most proximal projection of the articular surface to the most distal projection of the verticuli of the trochlei. | cm | Measuring box | Von den Driesch, 1976 |  |
| Metatarsus | Bp | Breadth proximal. Maximum medial-lateral diameter of the proximal end, including the muscle attachments. | mm | Small calipers | Von den Driesch, 1976 |  |
| Metatarsus | Dp | Depth proximal. Maximum dorsal-plantar diameter of the proximal end, including the muscle attachments. | mm | Small calipers | Von den Driesch, 1976 |  |
| Metatarsus | SD | Smallest breadth of the diaphysis. Smallest mediolateral diameter of the diaphysis. | mm | Small calipers | Schild, 1962; Von den Driesch, 1976 |  |
| Metatarsus | CD | Smallest circumference of the diaphysis. Measured perpendicular to the longitudinal axis. | cm | Tape measure | Von den Driesch, 1976 |  |
| Metatarsus | Bd | Breadth distal. Maximum medial-lateral diameter of the distal end measured from the most lateral projections of the medial and lateral epicondyles. Perpendicular to the longitudinal axis. | mm | Small calipers | Von den Driesch, 1976 |  |
| Metatarsus | BTm | Breadth of the medial trochlea. Greatest medial-lateral diameter of the medial trochlea, perpendicular to the longitudinal axis. | mm | Small calipers | Telldahl et al., 2012 | BFdm |
| Metatarsus | BTl | Breadth of the lateral trochlea. Greatest medial-lateral diameter of the lateral trochlea, perpendicular to the longitudinal axis. | mm | Small calipers | Telldahl et al., 2013 | BFdl |
| Metatarsus | DVm | Depth of the medial verticulus. Greatest dorsal-plantar diameter of the medial verticulus. | mm | Small calipers | Davis, 1996; SGWP in Popkin et al., 2012 | DVM |
| Metatarsus | DVl | Depth of the lateral verticulus. Greatest dorsal-plantar diameter of the lateral verticulus. | mm | Small calipers | Davis, 1996; SGWP in Popkin et al., 2012 | DVL |
| Metatarsus | BAp | Breadth of the proximal articular surface. Greatest medial-lateral diameter of the proximal articular facet, perpendicular to the longitudinal axis. | mm | Small calipers | SGWP in Popkin et al., 2012 | BFP |
| Metatarsus | BDF | Breadth of the diaphysis along the distal line of fusion. Greatest medial-lateral diameter of the fusion site of the distal end. | mm | Small calipers | Popkin et al., 2012 | BdFus |
| Metatarsus | BA | Breadth between the articular crests. Measured between the most distal points of the crests. | mm | Small calipers | Telldahl et al., 2012 | Bcr |
| Metatarsus | GCD | Greatest circumference of the diaphysis. | cm | Tape measure | Van den Berg et al. 2023 |  |
| Metatarsus | GDD | Greatest depth of the diaphysis. | mm | Small calipers | Van den Berg et al. 2023 |  |
| Metatarsus | SDD | Smallest depth of the diaphysis. Smallest dorsal-plantar diameter of the diaphysis. | mm | Small calipers | Van den Berg et al. 2023 |  |
| Metatarsus | PL | Physiological length. Measured from the most distal point of the proximal medial articular surface to the most distal projection of the medial epicondyle of the medial trochlea. | cm | Curved calipers | Van den Berg et al. 2023 |  |
| Metatarsus | DFp | Depth of the proximal articular facet. Measured from the medial facet, not perpendicular to the longitudinal axis but as shown in Fig. 8e. | mm | Small calipers | Van den Berg et al. 2023 |  |
| Pelvis | GL | Greatest length of one half. Measured from the most cranial projection of the iliac crest to the most caudal projection of the tuber ischiadicum. Important that the epiphyseal parts of the tuber coxae and the tuber ischiadicum have fused. | cm | Measuring box | Von den Driesch, 1976 |  |
| Pelvis | LA | Length of the acetabulum, including the lip. Across the facies lunata, measured in the directions of the ischium and os ilium, including the lip, and in the direction of the os ilium, the measurement is extended to the dent of the lateral musculus rectus femoris attachment site. | mm | Small calipers | Von den Driesch, 1976 |  |
| Pelvis | LAR | Length of the acetabulum on the rim. Across the facies lunata, measured in the directions of the ischium and os ilium, measured on the inside of the rim of the acetabular articular surface with the femoral caput. | mm | Small calipers | Von den Driesch, 1976 |  |
| Pelvis | LS | Length of the symphysis. Only when the two pelvic halves have not fused. | mm | Small calipers | Von den Driesch, 1976 |  |
| Pelvis | SH | Smallest height of the shaft ilium. The maximum dorsal-ventral diameter on the most constricted site of the shaft ilium. | mm | Small calipers | Von den Driesch, 1976 |  |
| Pelvis | SB | Smallest breadth of the shaft ilium. The minimum medial-lateral diameter on the most constricted site of the shaft ilium. | mm | Small calipers | Von den Driesch, 1976 |  |
| Pelvis | SC | Smallest circumference of the shaft ilium. | cm | Tape measure | Von den Driesch, 1976 |  |
| Pelvis | LFo | Inner length of the foramen obturatum. Measured across from the most cranial point close to the acetabulum to the most caudal towards the ischium. | mm | Small calipers | Von den Driesch, 1976 |  |
| Pelvis | GBTc | Greatest breadth across the tubera coxarum. Only when the two pelvic halves have fused. | cm | Measuring box | Von den Driesch, 1976 |  |
| Pelvis | GBA | Greatest breadth across the acetabula. Measured from the most lateral projection. Only when the two pelvic halves have fused. | cm | Measuring box | Von den Driesch, 1976 |  |
| Pelvis | GBTi | Greatest breadth across the tubera ischiadica. Only when the two pelvic halves have fused. | cm | Measuring box | Von den Driesch, 1976 |  |
| Pelvis | SBl | Smallest breadth across the bodies of the ischia. Only when the two pelvic halves have fused. | mm | Small calipers | Von den Driesch, 1976 |  |
| Pelvis | DPmin | Minimum diameter of the pubis shaft. | mm | Small calipers | Davis, 1996 | SHPu |
| Pelvis | DAm | Depth of the medial rim of the acetabulum. Measured as the ventral-medial border of the acetabulum. It is essential to take into account that the rim is pronounced in some individuals while vague in others; do not measure towards the "bulge" resulting from the negative projection of the acetabulum. | mm | Small calipers | Davis, 1996 | MRDA |
| Pelvis | DPS | Greatest depth of the pubic symphysis. Along the dorsal-ventral axis. | mm | Small calipers | Van den Berg et al. 2023 |  |

**Fig. SI1** Measurements taken from the humerus (see Table 2 for full definitions). The drawings show a right-side humerus in the (**a**) caudal, (**b**) medial, (**c**) cranial, (**d**) lateral, (**e**) proximal, and (**f**) distal view (Van den Berg et al. 2023, Fig. 3, reproduced with permission).

**Fig. SI2** Measurements taken from the radioulna (see Table 2 for full definitions). The drawings show a left-side radioulna in the (**a**) dorsal, (**b**) medial, (**c**) palmar, (**d**) lateral, (**e**) proximal, and (**f**) distal view (Van den Berg et al. 2023, Fig. 4, reproduced with permission).

**Fig. SI3** Measurements taken from the metacarpus (see Table 2 for full definitions). The drawings show a left-side metacarpus in the (**a**) dorsal, (**b**) medial, (**c**) palmar, (**d**) lateral, (**e**) proximal, and (**f**) distal view (Van den Berg et al. 2023, Fig. 5, reproduced with permission).

**Fig. SI4** Measurements taken from the femur (see Table 2 for full definitions). The drawings show a right-side femur in (**a**) cranial, (**b**) medial, (**c**) caudal, (**d**) lateral, (**e**) proximal, and (**f**) distal view (Van den Berg et al. 2023, Fig. 6, reproduced with permission).

**Fig. SI5** Measurements taken from the tibia (see Table 2 for full definitions). The drawings show a right-side tibia in the (**a**) dorsal, (**b**) medial, (**c**) plantar, (**d**) lateral, (**e**) proximal, and (**f**) distal view (Van den Berg et al. 2023, Fig. 7, reproduced with permission).

**Fig. SI6** Measurements taken from the metatarsus (see Table 2 for full definitions). The drawings show a left-side metatarsus in the (**a**) dorsal, (**b**) medial, (**c**) plantar, (**d**) lateral, (**e**) proximal, and (**f**) distal view (Van den Berg et al. 2023, Fig. 8, reproduced with permission).

**Fig. SI7** Measurements taken from the pelvis (see Table 2 for full definitions). The drawings show a fused pelvis in the (**a**) ventral, (**b**) dorsal, (**c**) right lateral, and (**d**) left cranial view (Van den Berg et al. 2023, Fig. 9, reproduced with permission).

**SI1.1 Intra-observer measurement error**

We (Van den Berg et al. 2023) found that most measurements fell within the acceptable limit of the chosen 2.5% difference and that only few measurements fell between a 2.5 and 3.75% difference: the circumference of the humeral diaphysis with an error of 2.75%; the breadth of the proximal metacarpal articular facet with an error of 2.95%; the metatarsal breadth between the articular crests with an error of 3.0%; the proximal breadth of the metatarsus with an error of 3.5%; the greatest circumference of the metatarsal diaphysis with an error of 3.0%; the smallest breadth of the metatarsal diaphysis with an error of 2.75%; the greatest breadth of the pelvic acetabula with an error of 2.75%; the minimum diameter of the pelvic pubic shaft with an error of 3.5% and; the greatest depth of the pelvic pubic symphysis with an error of 3.6%. A total of 4 measurements exceeded our chosen threshold by a larger margin: the distal femoral depth with an error of 5.3%; the greatest depth of the metatarsal diaphysis with an error of 3.95%; the pelvic depth of the medial rim of the acetabulum with an error of 4.85% and; the smallest breadth across the pelvic bodies of the ischia with an error of 4.05%.

**Table SI2** The selection of variables for variable reduction for whole bones and the selection of variables for variable reduction for proximal, distal, and shaft bone parts for the limb bones for the classification model based on Mosimann’s (1970) isometric Size and Shape

| **Element** | **Measurement** | **Proximal/distal** | **Shaft variable selection** | **Complete bone variable selection** |
| --- | --- | --- | --- | --- |
| Humerus | GL | No | No | No |
| Humerus | GLC | No | No | Yes |
| Humerus | GLl | No | No | No |
| Humerus | Bp | Proximal | No | Yes |
| Humerus | SD | No | Yes | Yes |
| Humerus | CD | No | Yes | No |
| Humerus | Bd | Distal | No | No |
| Humerus | BT | Distal | No | Yes |
| Humerus | HT | Distal | No | Yes |
| Humerus | DC | Proximal | No | Yes |
| Humerus | HTC | Distal | No | No |
| Humerus | Dp | Proximal | No | Yes |
| Humerus | Dd | Distal | No | Yes |
| Humerus | PL | No | No | No |
| Femur | GL | No | No | No |
| Femur | GLC | No | No | Yes |
| Femur | Bp | Proximal | No | Yes |
| Femur | DC | Proximal | No | Yes |
| Femur | SD | No | Yes | Yes |
| Femur | SDD | No | Yes | No |
| Femur | CD | No | Yes | No |
| Femur | Bd | Distal | No | Yes |
| Femur | Dd | Distal | No | Yes |
| Femur | BT | Distal | No | Yes |
| Femur | PL | No | No | No |
| Metacarpus | GL | No | No | Yes |
| Metacarpus | Bp | Proximal | No | Yes |
| Metacarpus | Dp | Proximal | No | Yes |
| Metacarpus | SD | No | Yes | Yes |
| Metacarpus | CD | No | Yes | No |
| Metacarpus | Bd | Distal | No | Yes |
| Metacarpus | BTm | Distal | No | No |
| Metacarpus | BTl | Distal | No | No |
| Metacarpus | DVm | Distal | No | No |
| Metacarpus | DVl | Distal | No | No |
| Metacarpus | BAp | Proximal | No | No |
| Metacarpus | BDF | Distal | No | Yes |
| Metacarpus | BA | Distal | No | Yes |
| Metacarpus | GCD | No | No | No |
| Metacarpus | GDD | No | No | No |
| Metacarpus | SDD | Distal | No | No |
| Metacarpus | PL | No | No | No |
| Metacarpus | DFp | Proximal | No | No |
| Metatarsus | GL | No | No | Yes |
| Metatarsus | Bp | Proximal | No | Yes |
| Metatarsus | Dp | Proximal | No | Yes |
| Metatarsus | SD | No | Yes | Yes |
| Metatarsus | CD | No | Yes | No |
| Metatarsus | Bd | Distal | No | Yes |
| Metatarsus | BTm | Distal | No | No |
| Metatarsus | BTl | Distal | No | No |
| Metatarsus | DVm | Distal | No | No |
| Metatarsus | DVl | Distal | No | No |
| Metatarsus | BAp | Proximal | No | No |
| Metatarsus | BDF | Distal | No | Yes |
| Metatarsus | BA | Distal | No | Yes |
| Metatarsus | GCD | No | No | No |
| Metatarsus | GDD | No | No | No |
| Metatarsus | SDD | Distal | No | No |
| Metatarsus | PL | No | No | No |
| Metatarsus | DFp | Proximal | No | No |
| Radioulna | GL | No | No | No |
| Radioulna | PL | No | No | Yes |
| Radioulna | Bp | Proximal | No | Yes |
| Radioulna | BFp | Proximal | No | No |
| Radioulna | CD | No | No | No |
| Radioulna | Bd | Distal | No | Yes |
| Radioulna | SD | No | Yes | Yes |
| Radioulna | SDD | No | Yes | No |
| Radioulna | Dd | Distal | No | Yes |
| Radioulna | Dp | Proximal | No | Yes |
| Radioulna | LO | Proximal | No | Yes |
| Radioulna | SDO | Proximal | No | Yes |
| Tibia | GL | No | No | No |
| Tibia | Ll | No | No | Yes |
| Tibia | SD | No | Yes | Yes |
| Tibia | SDD | No | Yes | No |
| Tibia | CD | No | No | No |
| Tibia | Bd | Distal | No | No |
| Tibia | Dd | Distal | No | Yes |
| Tibia | Dp | Proximal | No | Yes |
| Tibia | PL | No | No | No |
| Tibia | BFp | Proximal | No | Yes |
| Tibia | BFd | Distal | No | Yes |

**Fig. SI8** The variable importance results of isometric size (iSize) and shape (iShape) analysis based on Mosimann’s (1970) protocol for the classification model of the humerus for *Rangifer tarandus fennicus*, domestic *Rangifer tarandus tarandus*, and wild *Rangifer tarandus tarandus*

**Fig. SI9** The variable importance results of isometric size (iSize) and shape (iShape) analysis based on Mosimann’s (1970) protocol for the classification model of the radioulna for *Rangifer tarandus fennicus*, domestic *Rangifer tarandus tarandus*, and wild *Rangifer tarandus tarandus*

**Fig. SI10** The variable importance results of isometric size (iSize) and shape (iShape) analysis based on Mosimann’s (1970) protocol for the classification model of the metacarpus for *Rangifer tarandus fennicus*, domestic *Rangifer tarandus tarandus*, and wild *Rangifer tarandus tarandus*

**Fig. SI11** The variable importance results of isometric size (iSize) and shape (iShape) analysis based on Mosimann’s (1970) protocol for the classification model of the femur for *Rangifer tarandus fennicus*, domestic *Rangifer tarandus tarandus*, and wild *Rangifer tarandus tarandus*

**Fig. SI12** The variable importance results of isometric size (iSize) and shape (iShape) analysis based on Mosimann’s (1970) protocol for the classification model of the tibia for *Rangifer tarandus fennicus*, domestic *Rangifer tarandus tarandus*, and wild *Rangifer tarandus tarandus*

**Fig. SI13** The variable importance results of isometric size (iSize) and shape (iShape) analysis based on Mosimann’s (1970) protocol for the classification model of the metatarsus for *Rangifer tarandus fennicus*, domestic *Rangifer tarandus tarandus*, and wild *Rangifer tarandus tarandus*

**Table SI3** The statistical comparison using iSize per element between samples from the original wild reindeer (Oppland/Dovre: Oppland and Oppland, Dovre) and from wild reindeer admixed with domestic stock (Hardangervidda: Buskerud and Hordaland) used in this study, showing no statistically significant difference between the iSizes of the different types of reindeer

| **Element** | **Contrast** | **Estimate** | **Conf.low** | **Conf.high** |
| --- | --- | --- | --- | --- |
| Humerus | mean(Hardangervidda, N = 10) - mean(Oppland/Dovre, N = 5) | -0.0284 | -0.0854 | 0.0093 |
| Radioulna | mean(Hardangervidda, N = 11) - mean(Oppland/Dovre, N = 5) | -0.0101 | -0.0844 | 0.0255 |
| Metacarpus | mean(Hardangervidda, N = 12) - mean(Oppland/Dovre, N = 5) | -0.0750 | -0.1409 | 0.0001 |
| Femur | mean(Hardangervidda, N = 12) - mean(Oppland/Dovre, N = 5) | -0.0112 | -0.0803 | 0.0190 |
| Tibia | mean(Hardangervidda, N = 11) - mean(Oppland/Dovre, N = 6) | -0.0644 | -0.1336 | 0.0029 |
| Metatarsus | mean(Hardangervidda, N = 13) - mean(Oppland/Dovre, N = 6) | -0.0522 | -0.1194 | 0.0053 |
| Pelvis | mean(Hardangervidda, N = 5) - mean(Oppland/Dovre, N = 2) | -0.0450 | -0.2360 | 0.0552 |
