# Supplementary figures and images for: Osteometric distinctions between domestic reindeer (Rangifer tarandus tarandus), wild mountain reindeer (R.t.t.), wild forest reindeer (R.t. fennicus), and the identification of castrated reindeer bones: Biometric explorations and archaeological methods
Source: Archaeol Anthropol Sci. 2025 Apr 1;17(4):94. doi: 10.1007/s12520-025-02198-3 (PMC11961526; doi:10.1007/s12520-025-02198-3)

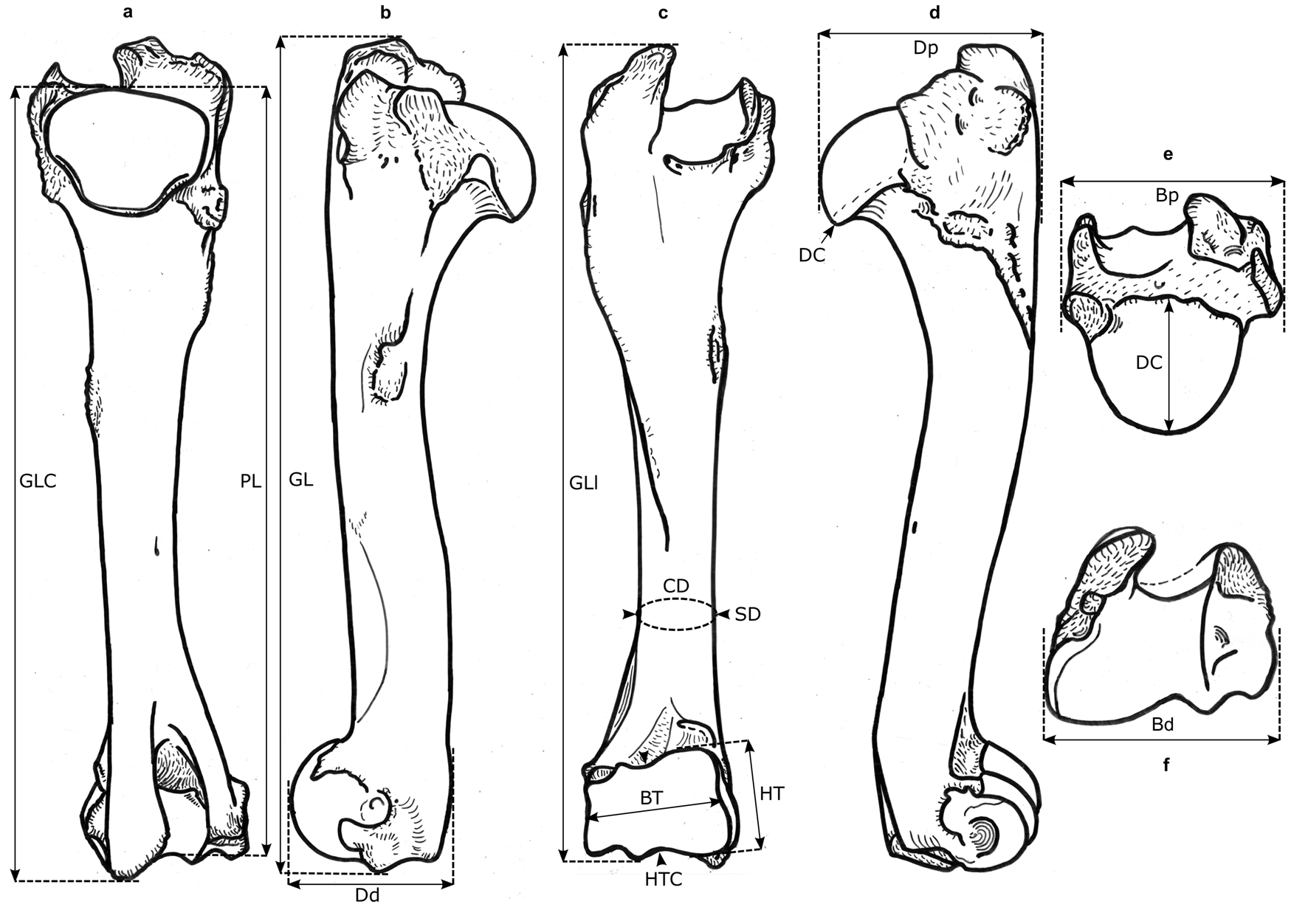

Supplement: Supplementary file 4 — Fig. SI1 [file 12520_2025_2198_Fig16_ESM.png]

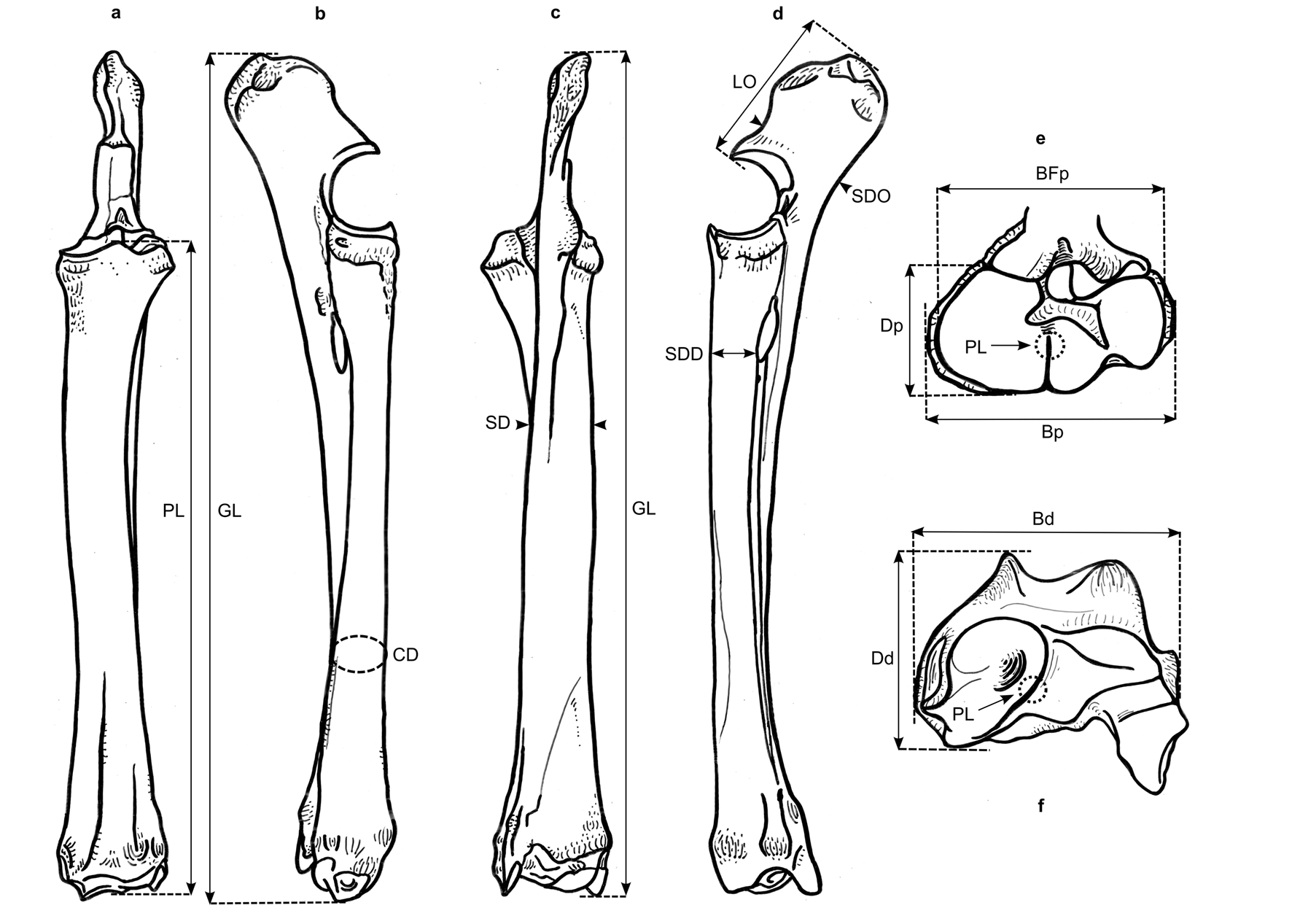

Supplement: Supplementary file 6 — Fig. SI2 [file 12520_2025_2198_Fig17_ESM.png]

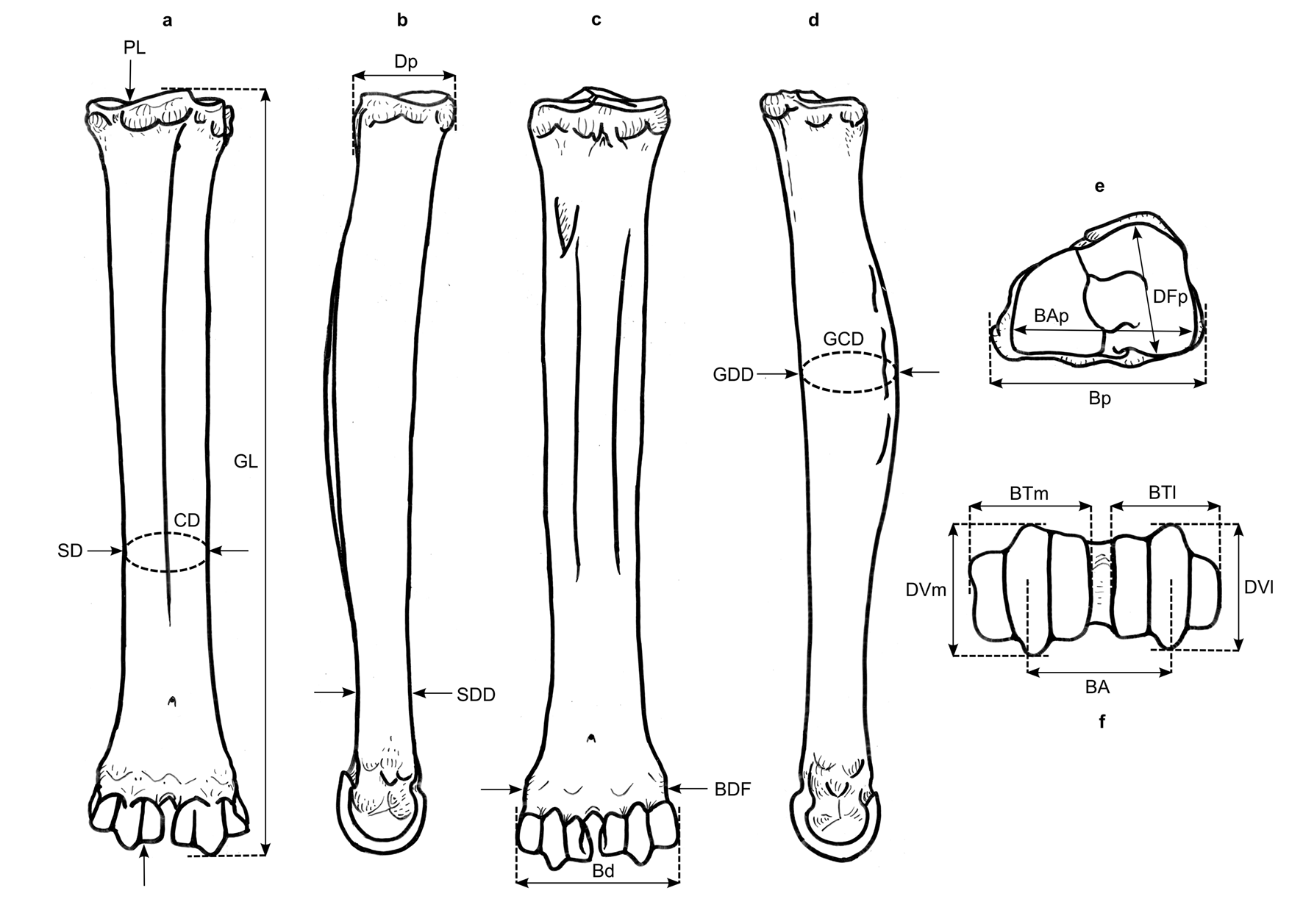

Supplement: Supplementary file 8 — Fig. SI3 [file 12520_2025_2198_Fig18_ESM.png]

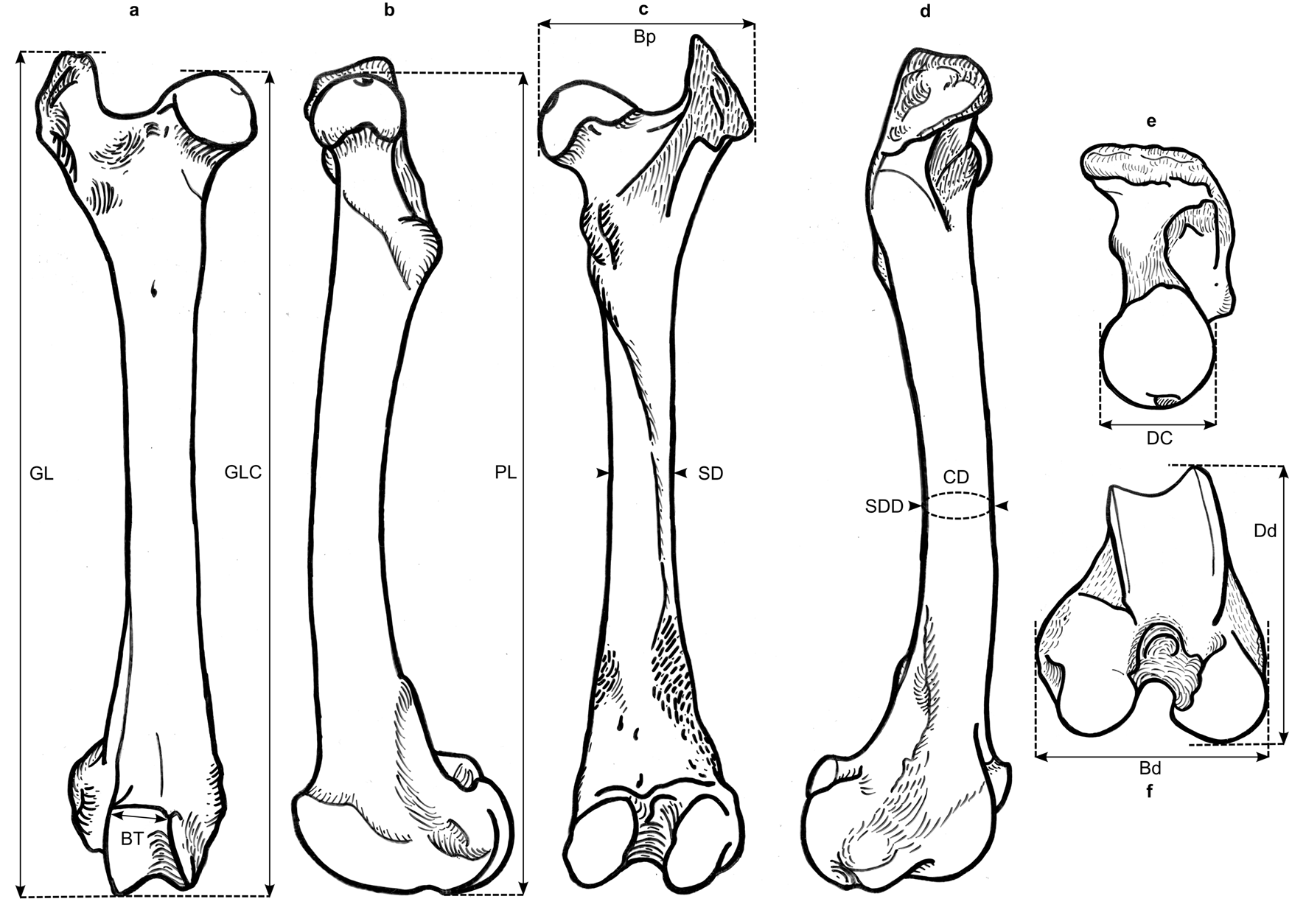

Supplement: Supplementary file 10 — Fig. SI4 [file 12520_2025_2198_Fig19_ESM.png]

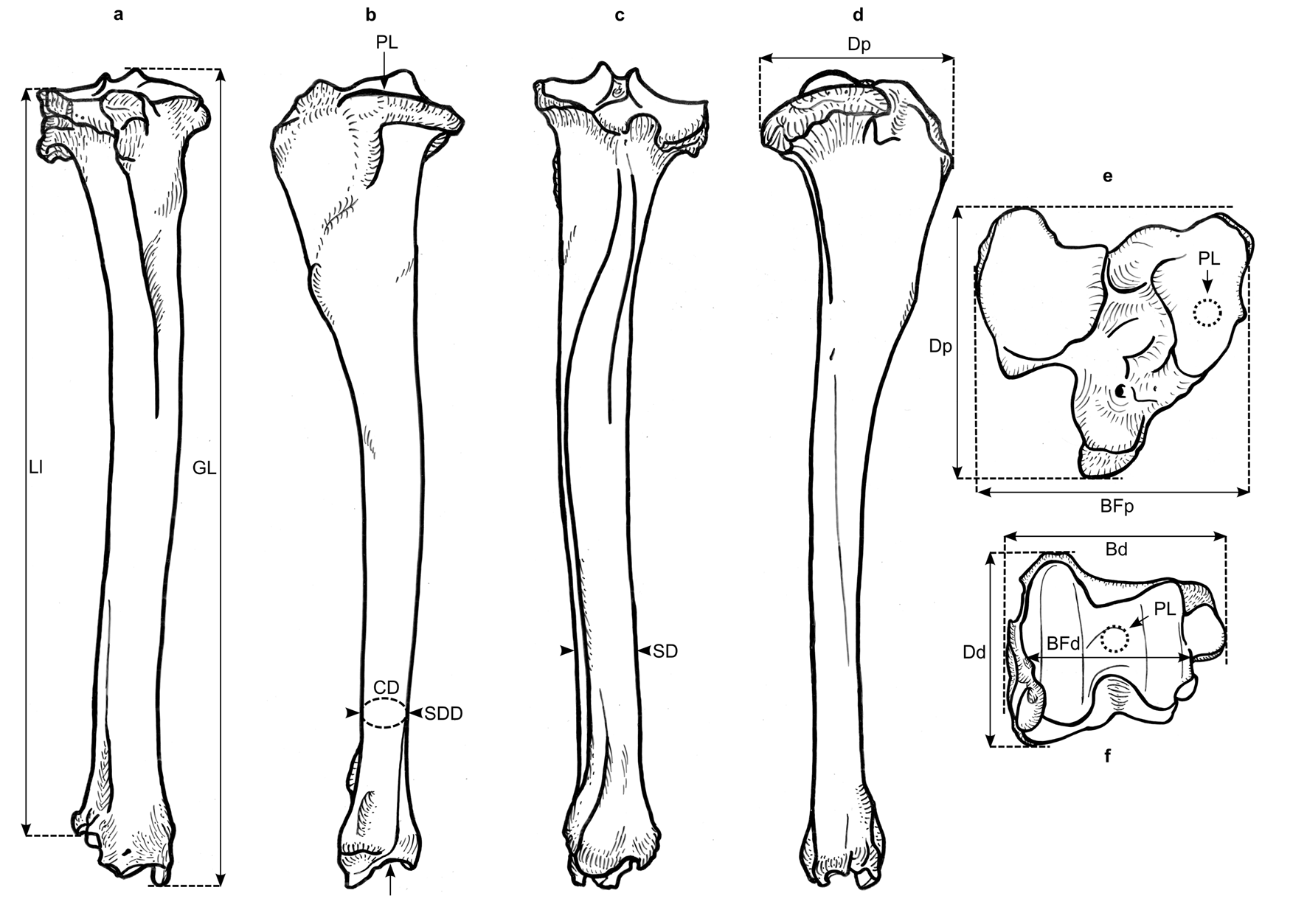

Supplement: Supplementary file 12 — Fig. SI5 [file 12520_2025_2198_Fig20_ESM.png]

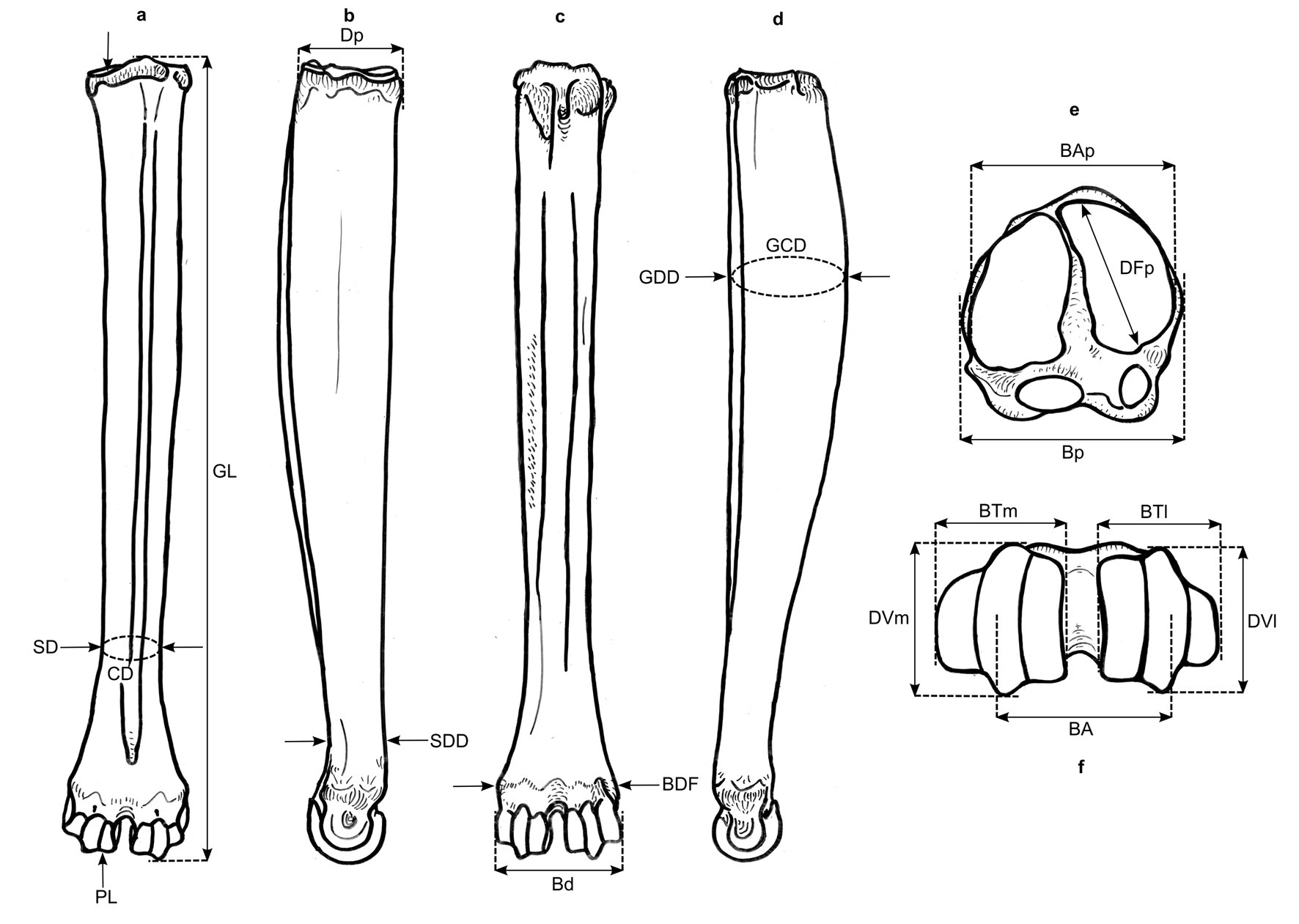

Supplement: Supplementary file 14 — Fig. SI6 [file 12520_2025_2198_Fig21_ESM.png]

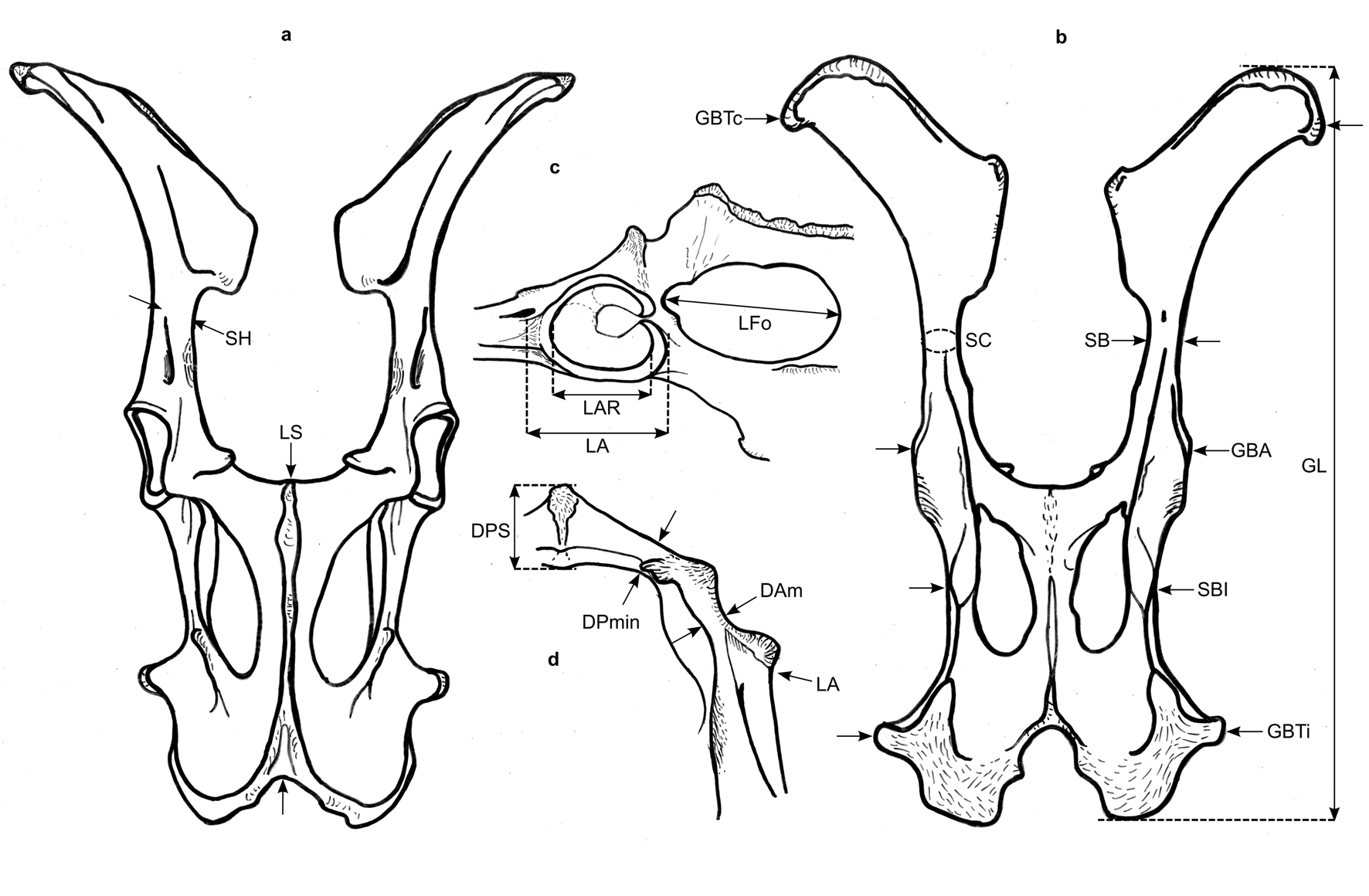

Supplement: Supplementary file 16 — Fig. SI7 [file 12520_2025_2198_Fig22_ESM.png]

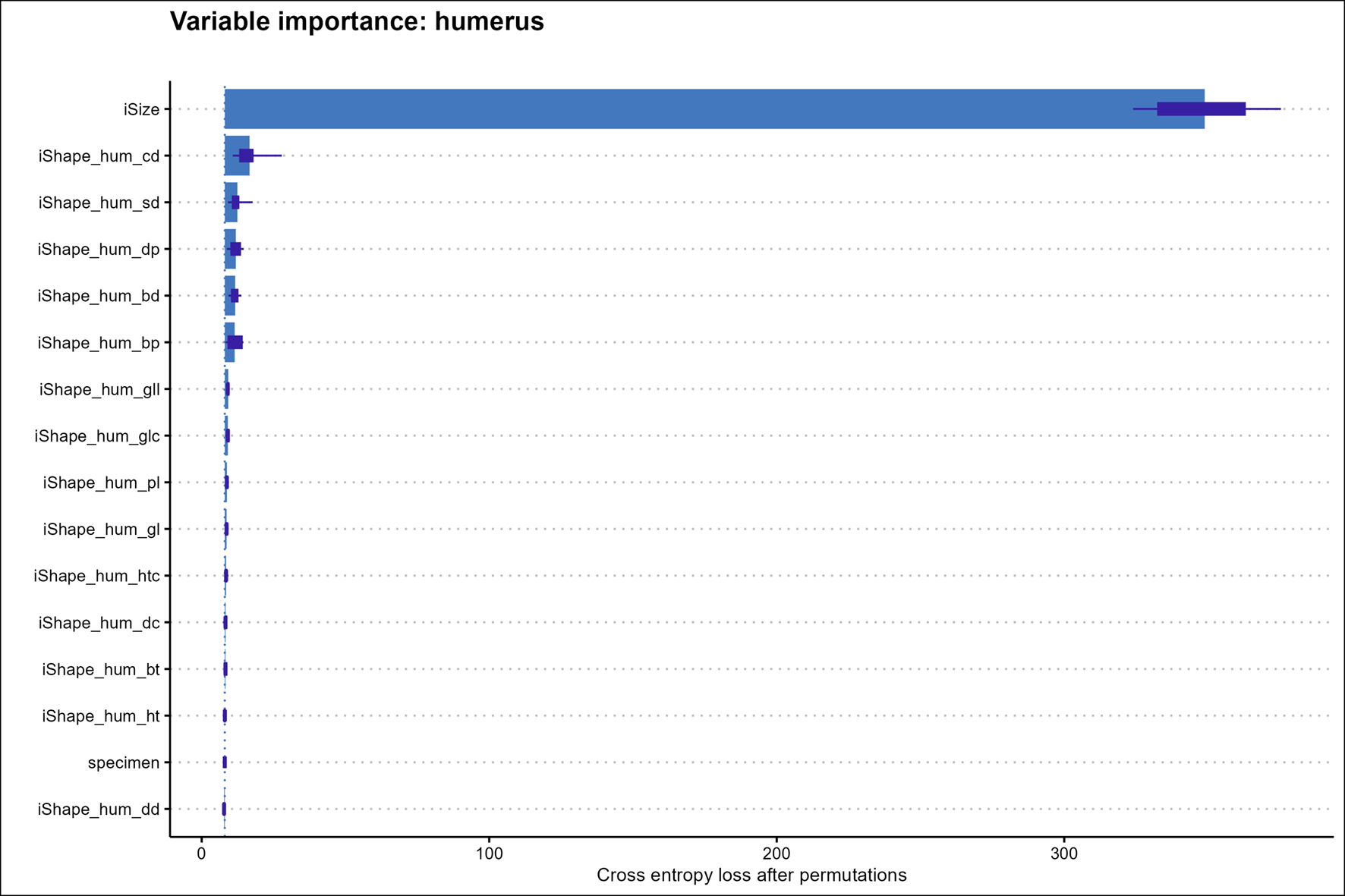

Supplement: Supplementary file 18 — Fig. SI8 [file 12520_2025_2198_Fig23_ESM.png]

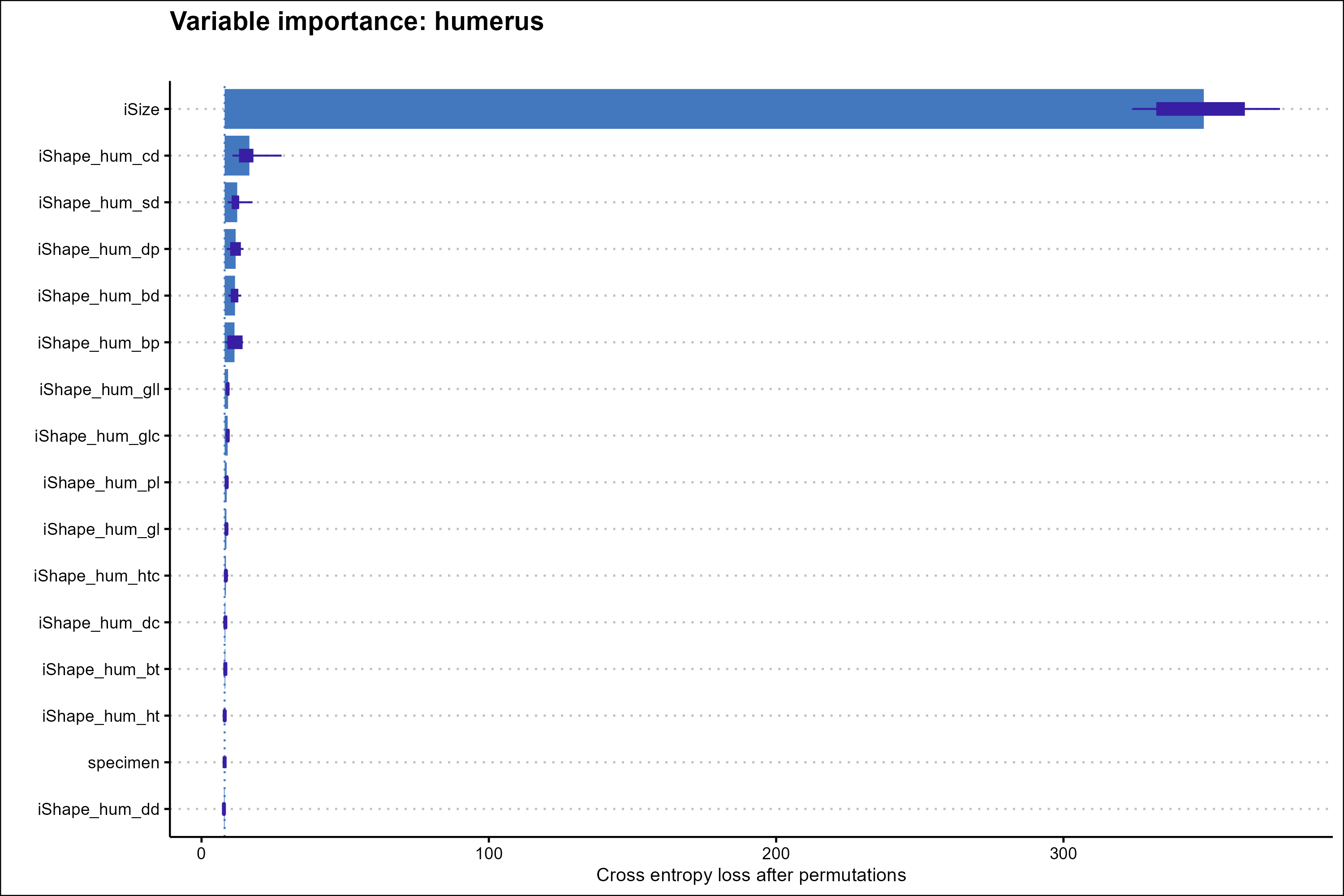

Supplement: Supplementary file 19 — High resolution image (TIFF 17579 KB) [file 12520_2025_2198_MOESM11_ESM.tiff]

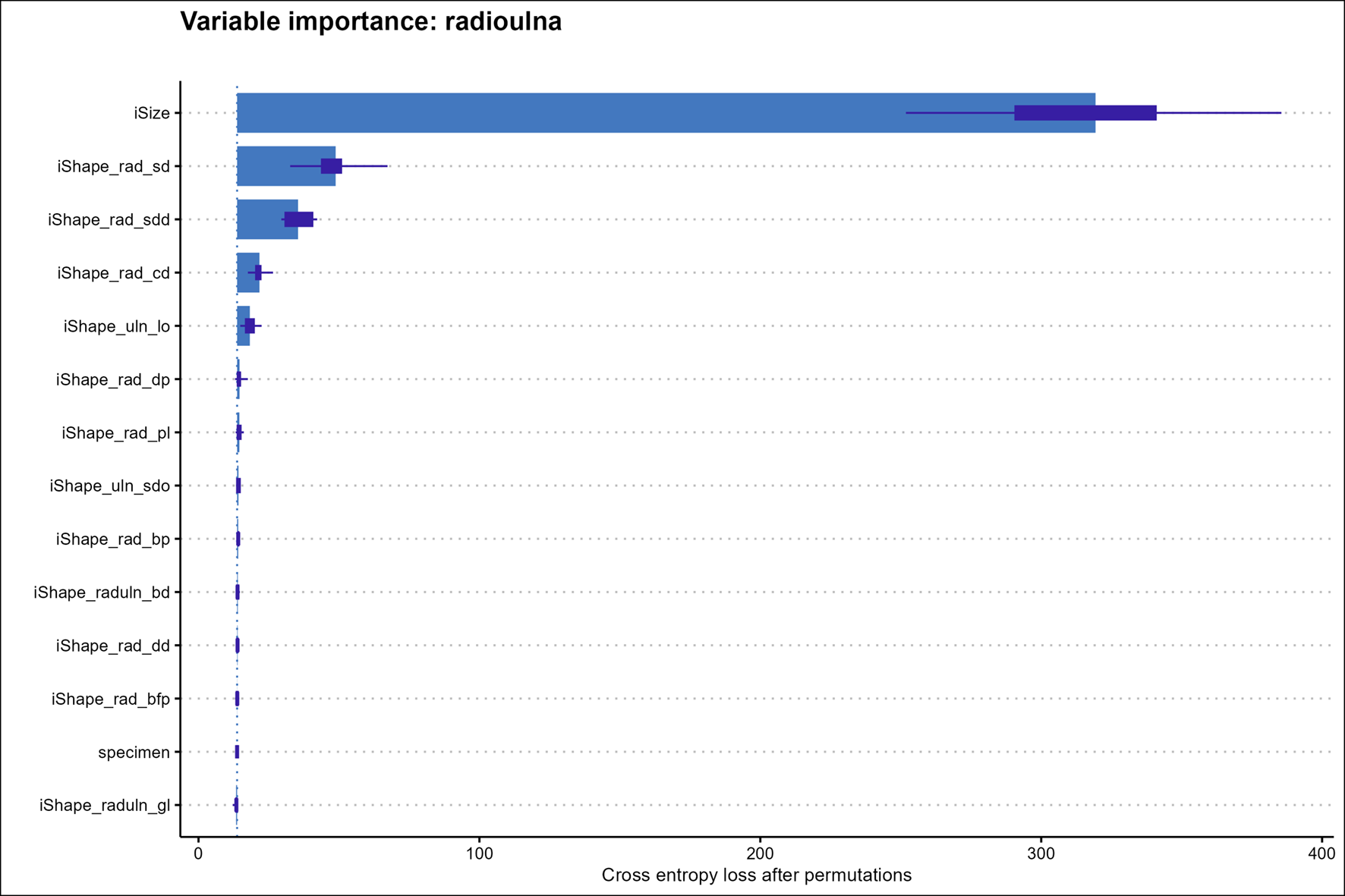

Supplement: Supplementary file 20 — Fig. SI9 [file 12520_2025_2198_Fig24_ESM.png]

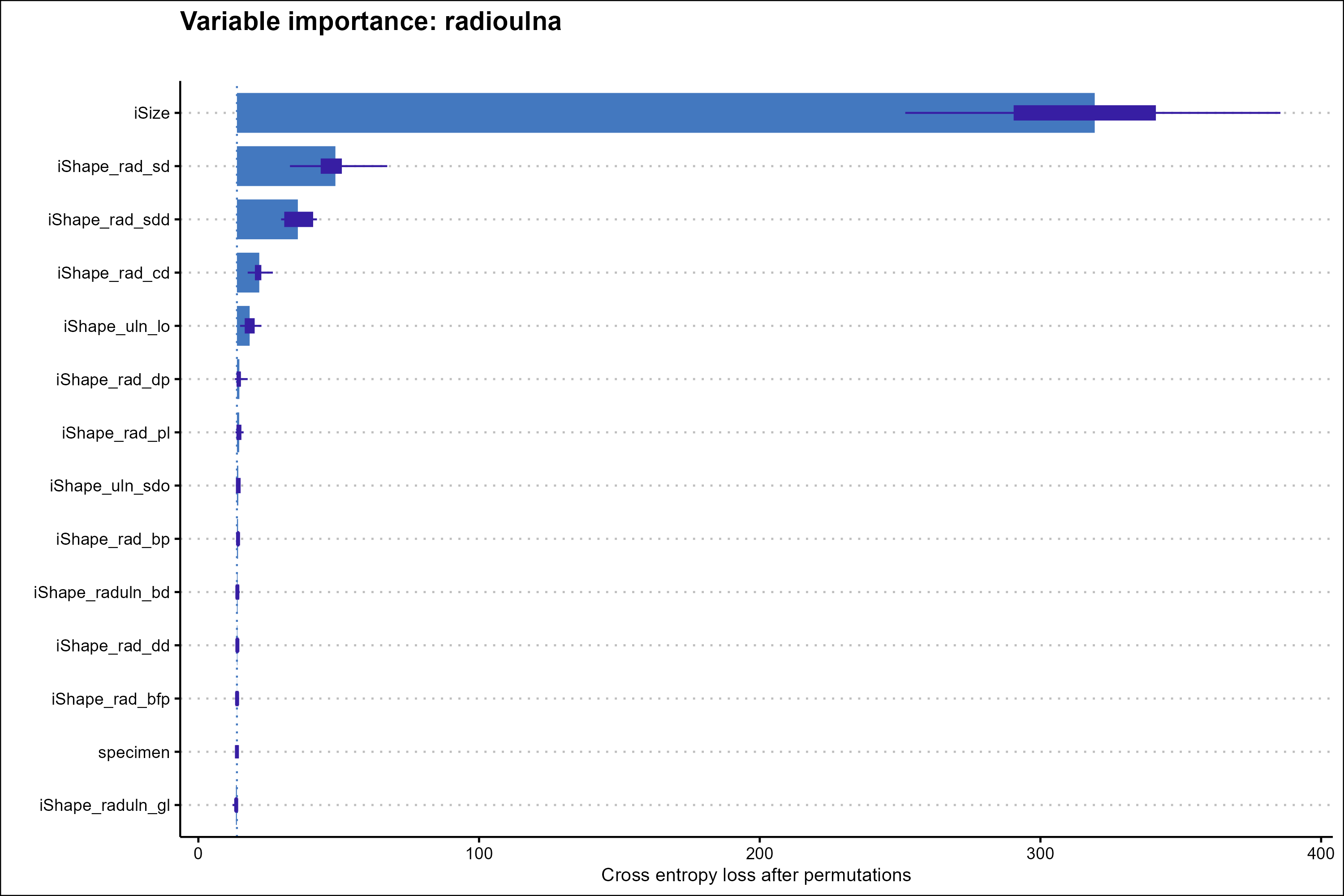

Supplement: Supplementary file 21 — High resolution image (TIFF 17579 KB) [file 12520_2025_2198_MOESM12_ESM.tiff]

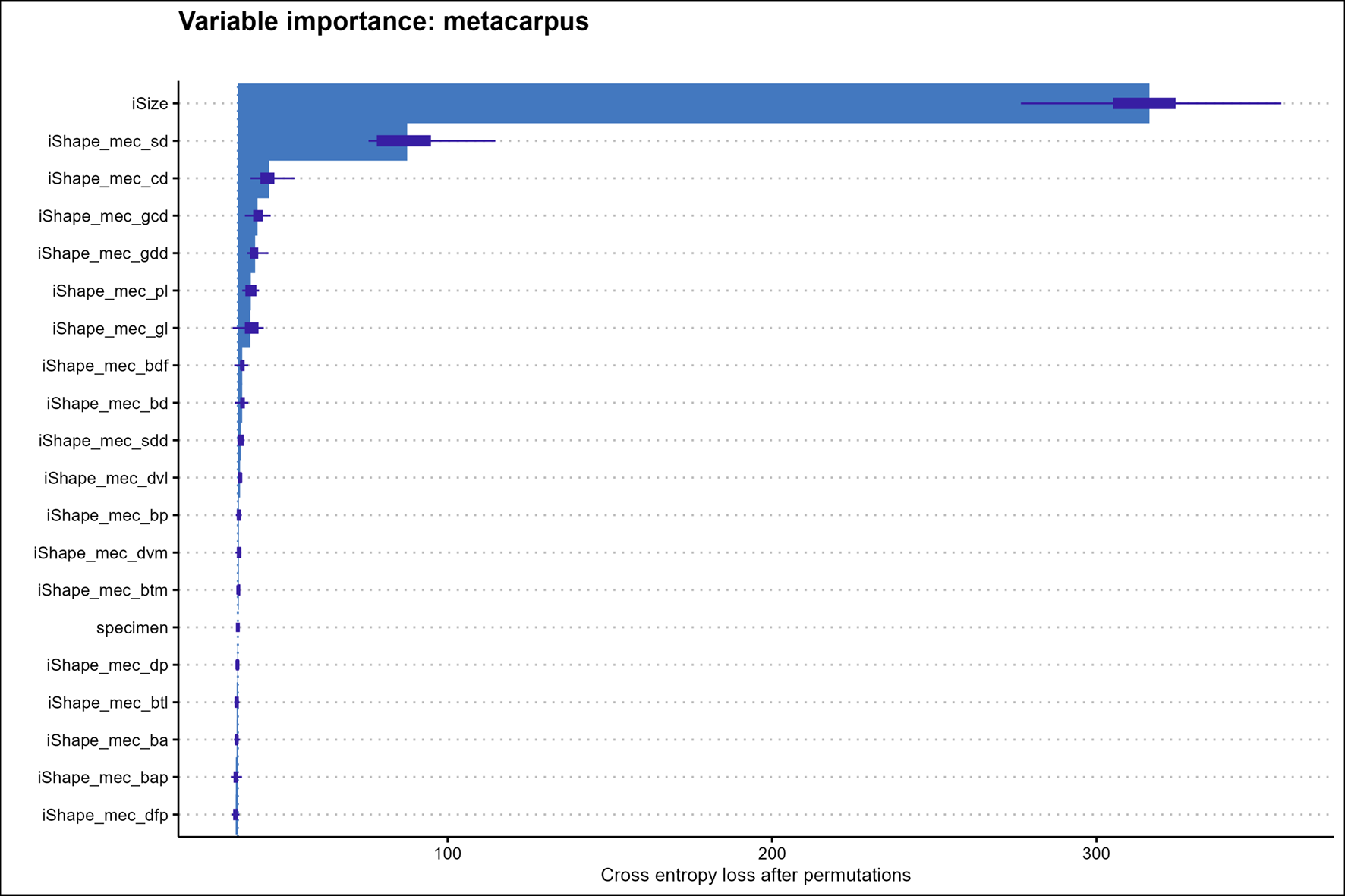

Supplement: Supplementary file 22 — Fig. SI10 [file 12520_2025_2198_Fig25_ESM.png]

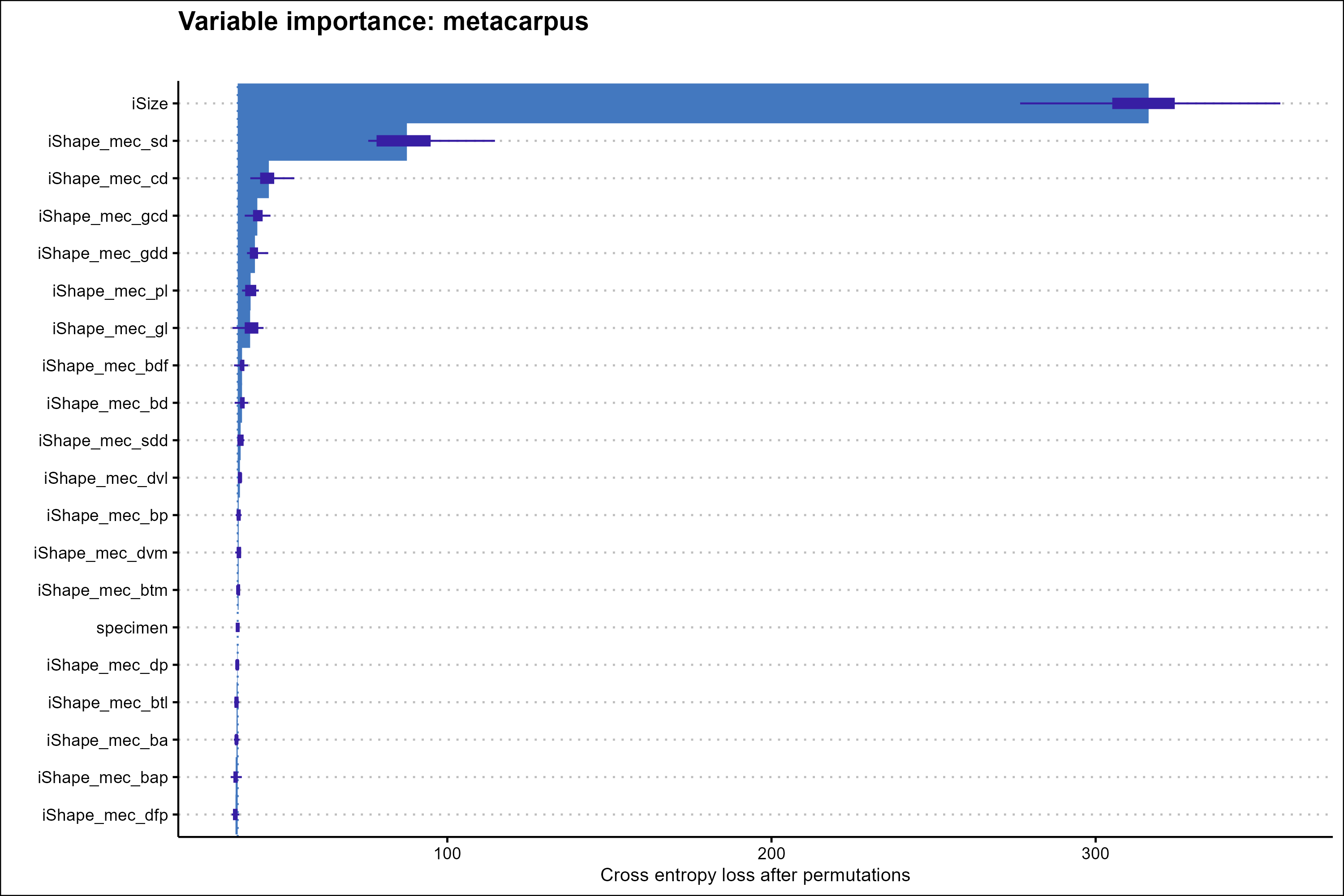

Supplement: Supplementary file 23 — High resolution image (TIFF 17579 KB) [file 12520_2025_2198_MOESM13_ESM.tiff]

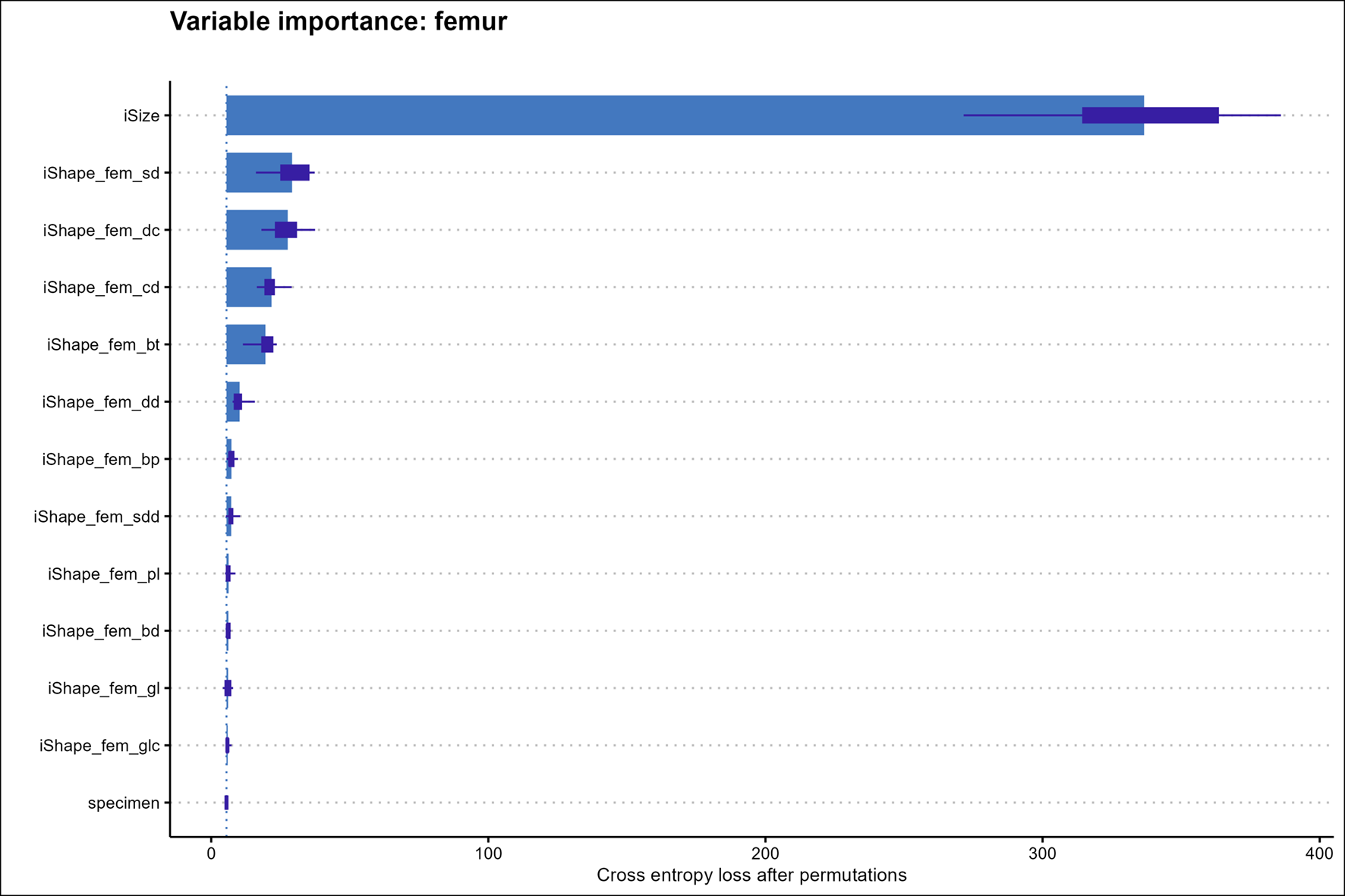

Supplement: Supplementary file 24 — Fig. SI11 [file 12520_2025_2198_Fig26_ESM.png]

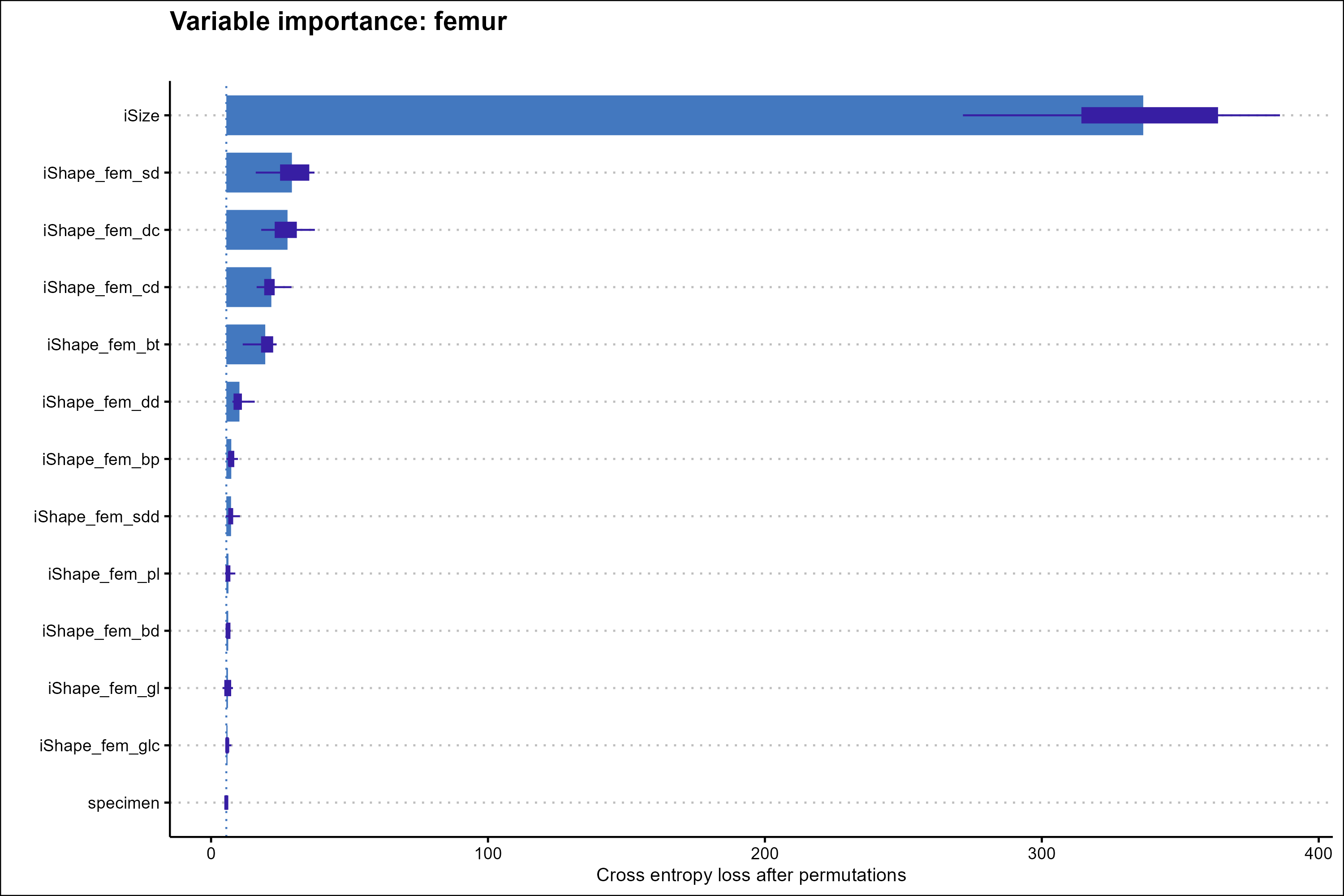

Supplement: Supplementary file 25 — High resolution image (TIFF 17579 KB) [file 12520_2025_2198_MOESM14_ESM.tiff]

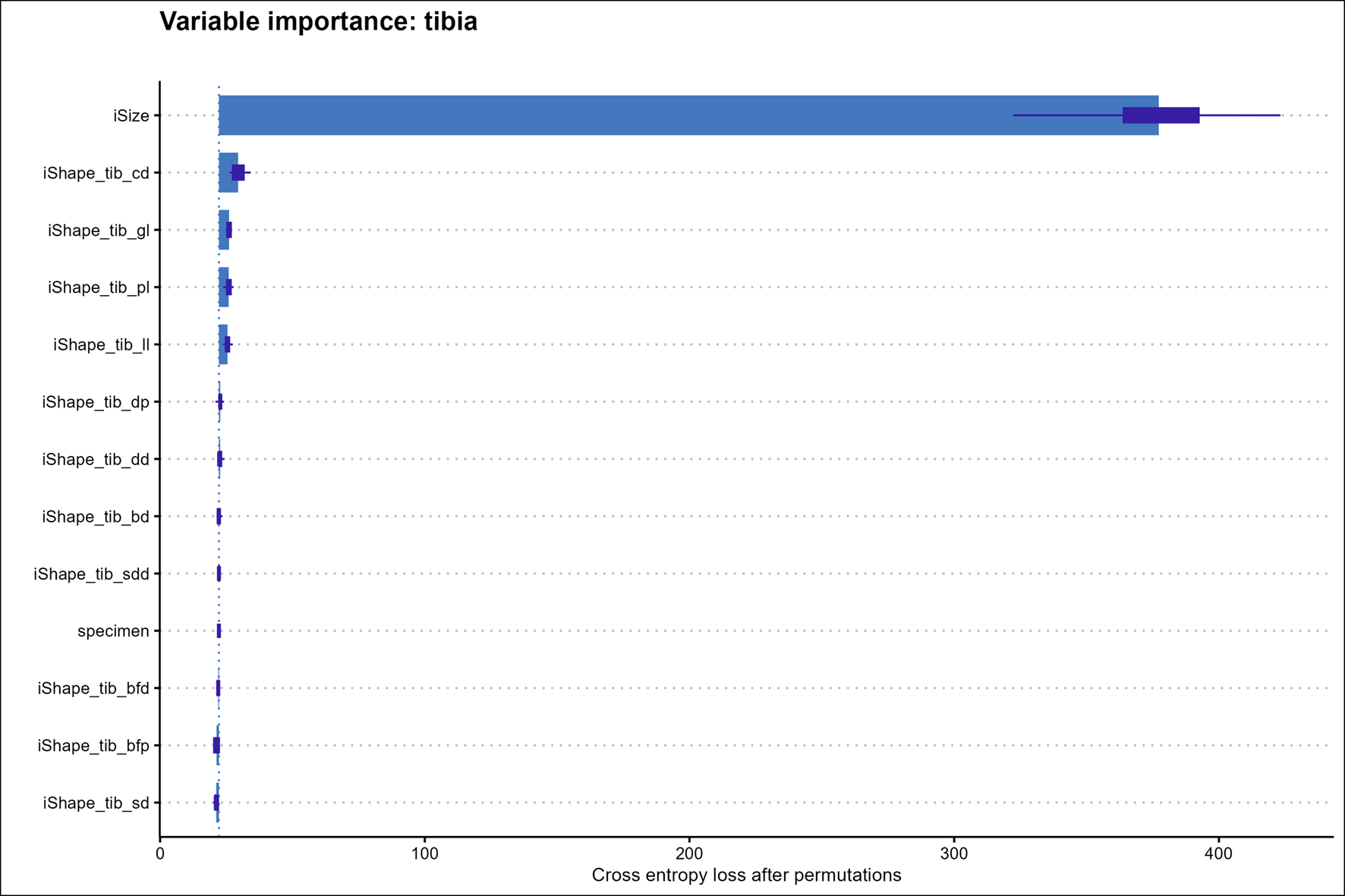

Supplement: Supplementary file 26 — Fig. SI12 [file 12520_2025_2198_Fig27_ESM.png]

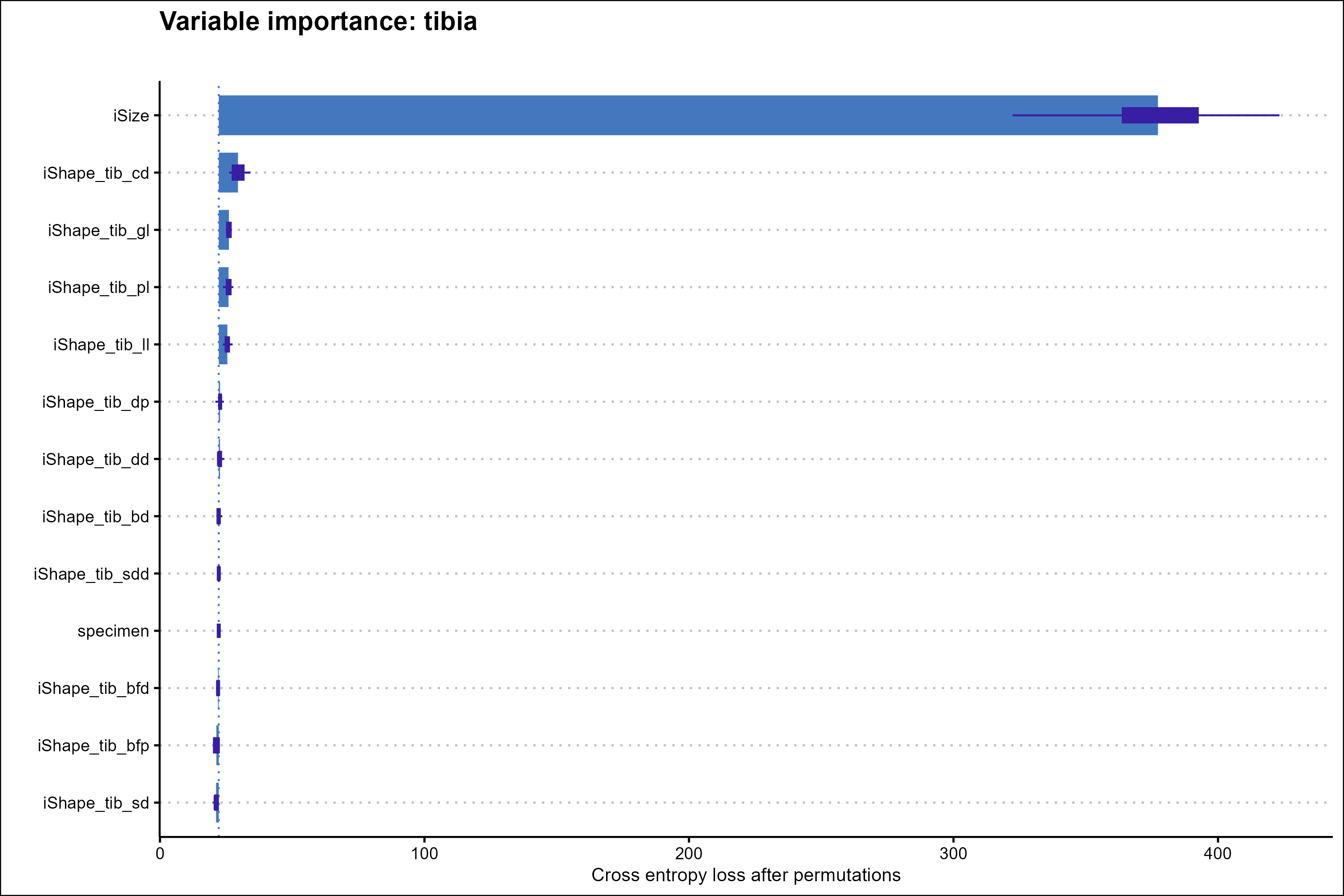

Supplement: Supplementary file 27 — High resolution image (TIFF 17579 KB) [file 12520_2025_2198_MOESM15_ESM.tiff]

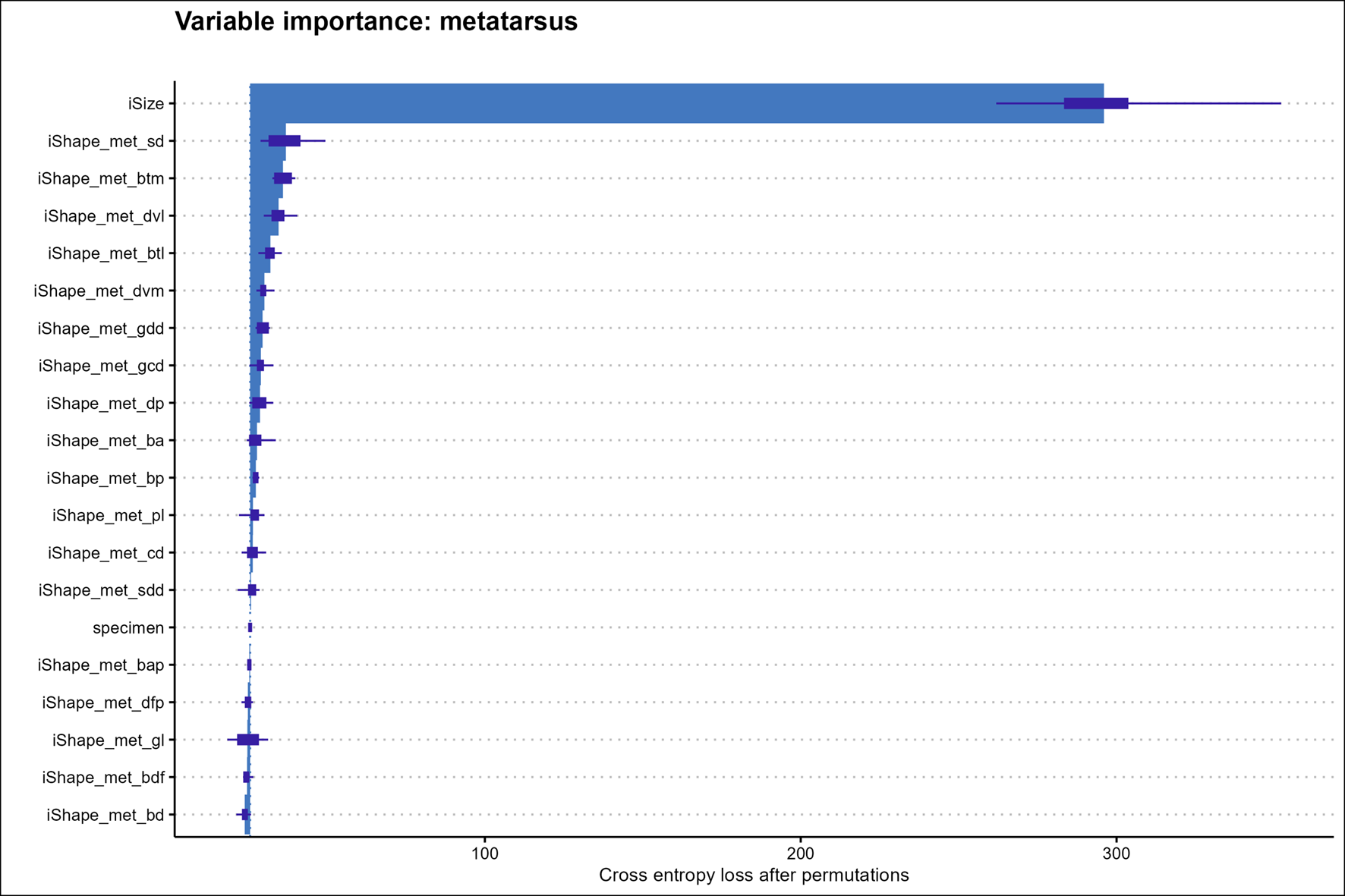

Supplement: Supplementary file 28 — Fig. SI13 [file 12520_2025_2198_Fig28_ESM.png]

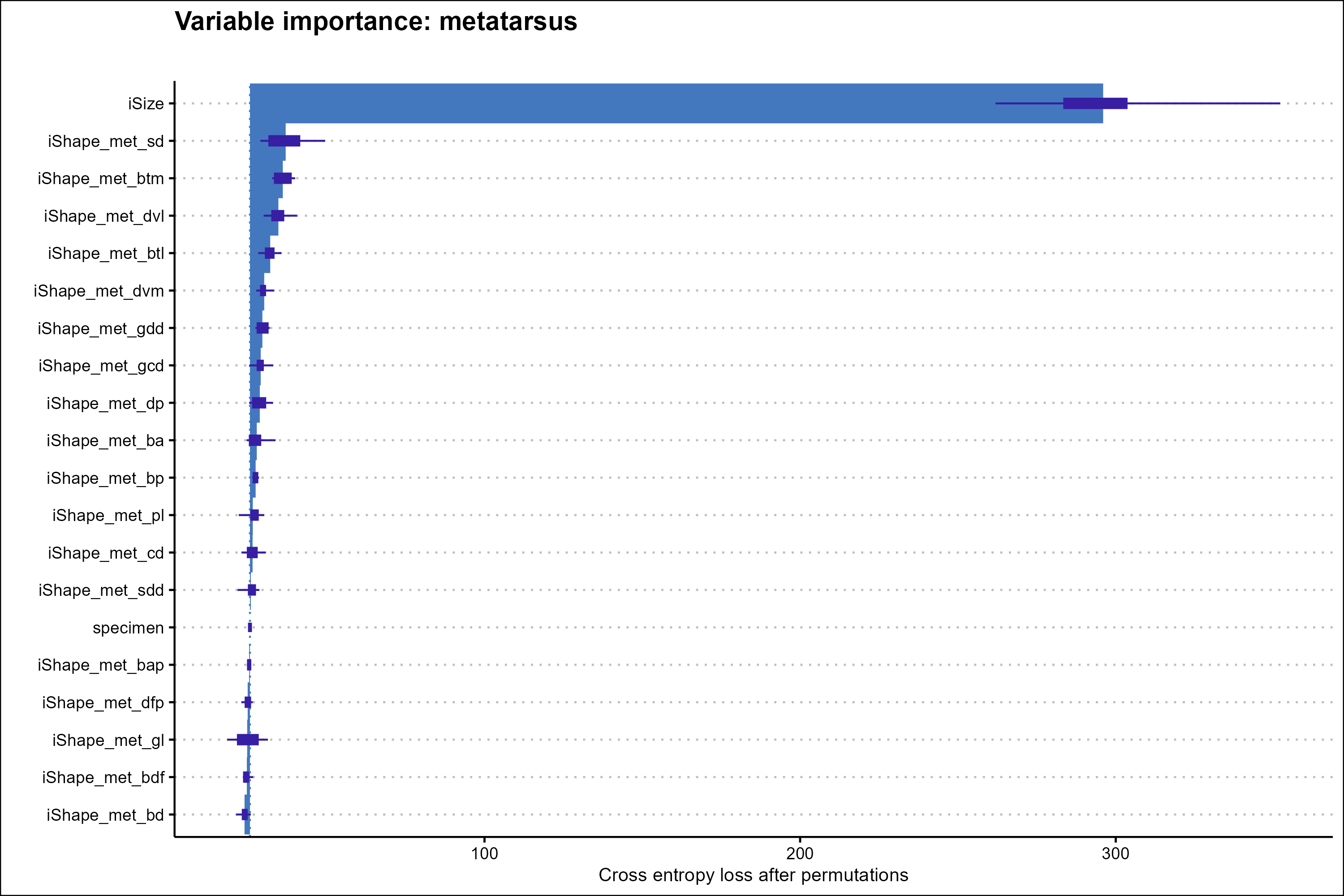

Supplement: Supplementary file 29 — High resolution image (TIFF 17579 KB) [file 12520_2025_2198_MOESM16_ESM.tiff]
